# Efficacy of an Educational Material on Second Primary Cancer Screening Practice for Cancer Survivors: A Randomized Controlled Trial

**DOI:** 10.1371/journal.pone.0033238

**Published:** 2012-03-29

**Authors:** Dong Wook Shin, Juhee Cho, Young Woo Kim, Jae Hwan Oh, Seok Won Kim, Ki-Wook Chung, Woo-Yong Lee, Jeong Eon Lee, Eliseo Guallar, Won-Chul Lee

**Affiliations:** 1 Department of Family Medicine & Health Promotion Center, Seoul National University Hospital, Seoul, Korea; 2 Cancer Survivorship Clinic, Cancer Hospital, Seoul National University Hospital, Seoul, Korea; 3 Cancer Education Center, Samsung Comprehensive Cancer Center, Samsung Medical Center, Sungkyunkwan University School of Medicine, Seoul, Korea; 4 Samsung Advanced Institute for Health Sciences and Technology, Sungkyunkwan University, Seoul, Korea; 5 Departments of Health, Behavior, and Society and Epidemiology, Johns Hopkins Bloomberg School of Public Health, Baltimore, Maryland, United States of America; 6 Cancer Hospital & Research Institute, National Cancer Center, Goyang, Korea; 7 Department of Surgery, Samsung Medical Center Sungkyunkwan University School of Medicine, Seoul, Korea; 8 Department of Medicine and Welch Center for Prevention, Epidemiology, and Clinical Research, Johns Hopkins Medical Institutions, Baltimore, Maryland, United States of America; 9 Department of Preventive Medicine, The Catholic University of Korea, Seoul, Korea; Academic Medical Center, Netherlands

## Abstract

**Background:**

Cancer surivors have limited knowledge about second primary cancer (SPC) screening and suboptimal rates of completion of screening practices for SPC. Our objective was to test the efficacy of an educational material on the knowledge, attitudes, and screening practices for SPC among cancer survivors.

**Methods:**

Randomized, controlled trial among 326 cancer survivors from 6 oncology care outpatient clinics in Korea. Patients were randomized to an intervention or an attention control group. The intervention was a photo-novel, culturally tailored to increase knowledge about SPC screening. Knowledge and attitudes regarding SPC screening were assessed two weeks after the intervention, and screening practices were assessed after one year.

**Results:**

At two weeks post-intervention, the average knowledge score was significantly higher in the intervention compared to the control group (0.81 vs. 0.75, P<0.01), with no significant difference in their attitude scores (2.64 vs. 2.57, P = 0.18). After 1 year of follow-up, the completion rate of all appropriate cancer screening was 47.2% in both intervention and control groups.

**Conclusion:**

While the educatinal material was effective for increasing knowledge of SPC screening, it did not promote cancer screening practice among cancer survivors. More effective interventions are needed to increase SPC screening rates in this population.

**Trial Registration:**

ClinicalTrial.gov NCT00948337

## Introduction

The high incidence of second primary cancers (SPC) [1], [2] and the impact of a SPC on survival [3] underscore the importance of cancer screening in the increasing number of cancer survivors. [4], [5] Studies in the U.S. [6], [7], [8] and Korea [9] found that cancer survivors were more likely to undergo cancer screening compared to people without cancer, but the screening rates in cancer survivors were still suboptimal [10]. There is thus considerable interest in identifying effective interventions to increase screening practices among cancer survivors. [9]

In a previous qualitative study, [11] we found that cancer survivors frequently had not heard about SPC and could not differentiate SPC from recurrence or metastasis of the primary tumor. Cancer survivors were not aware of an increased risk of SPC and had difficulty in distinguishing second cancer screening from routine surveillance tests after cancer treatment. However, they generally had positive attitudes towards cancer screening and said that they would have undergone screening for SPC if they had known about it. Overall, lack of knowledge seemed to be the critical barrier for SPC screening in this population, and we hypothesized simple intervention to cover such knowledge gap would be effective.

Therefore, we designed a printed educational material that incorporated cancer survivors' perspectives to increase their knowledge of SPC screening. This paper reports a multicenter randomized controlled trial that we conducted to assess the efficacy of an educational material on SPC screening practice among cancer survivors. As a secondary objective, we examined the impact of the intervention on short-term knowledge and attitudes about SPC.

## Methods

The protocol for this trial and supporting CONSORT checklist are available as supporting information; see Checklist S1 and Protocol S1.

### Study population

The trial was conducted in six specialized oncology care outpatient clinics located at two cancer centers in the Seoul metropolitan area, Korea. We included survivors of stomach, colorectal, breast and thyroid cancers, based on the high incidence and high 5-year survival rates of these cancers in Korea (**Table S1**). [12] Subjects were eligible to participate if they were 40 to 79 years of age, had a histologically confirmed diagnosis of cancer (stage I to III), had completed treatment for the primary cancer at least one year ago at the time of recruitment, had no evidence of recurrence, metastasis, or SPC, and had no evidence of hereditary cancer. We excluded cancer survivors with poor general performance status (Eastern Cooperative Oncology Group [ECOG] ≥3) or who were unable to read Korean.

**Figure 1:**
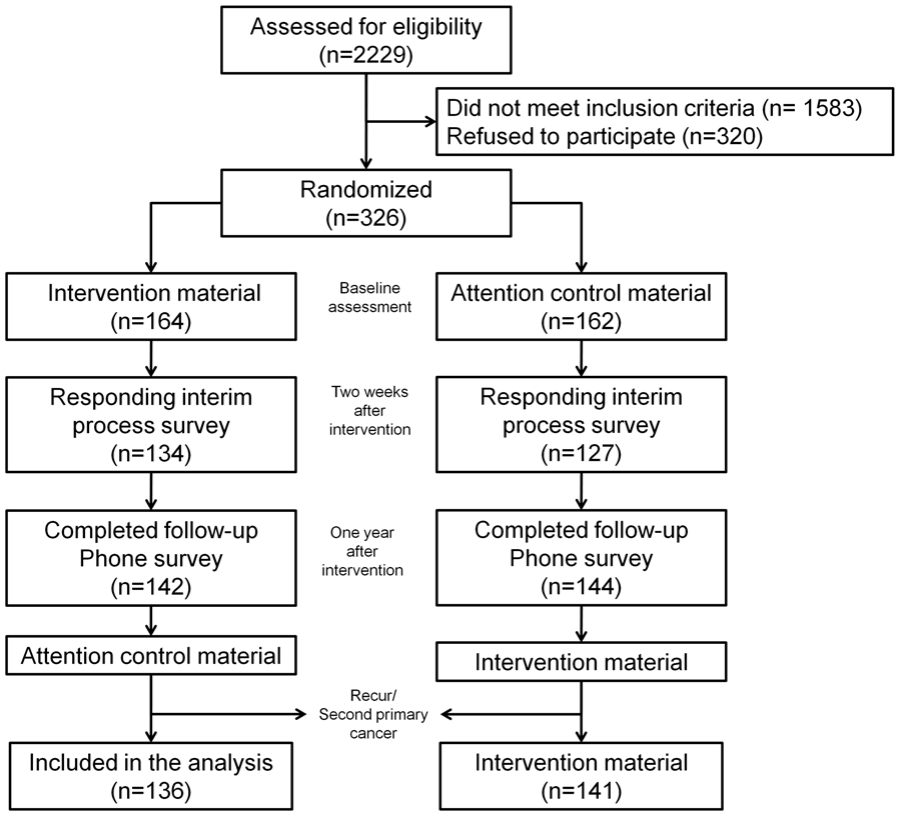
Flowchart of study participants.

We used electronic medical records to identify survivors who met the eligibility criteria and were scheduled to visit the participating outpatient clinics in 2009. Among 646 eligible cancer survivors who were approached during their regular follow-up visits, 326 (50.3%) agreed to participate in the study. Study participants were individually randomized to an intervention or an attention control group (**Figure 1**). Randomization tables were generated using a randomization program stratified by oncologist with blocks of size four. The randomized assignments were not revealed to study coordinators until after recruitment was completed.

### Ethics

Individual participants in this study gave written informed consent. The study protocol was approved by the Institutional Review Board of each participating center. All participants signed a written informed consent.

### Conceptual model and study interventions

This study was primarily based on the health belief model and social cognitive theory. [13] The health belief model addresses an individual's perceptions of the threat posed by a health problem, the benefits of avoiding the threat, and factors influencing the decision to act. [13] Social cognitive theory assumes a reciprocal interaction between cognitive factors (e.g. knowledge and attitudes), environmental factors (e.g. provider recommendation), and behaviors. Patient characteristics, such as primary diagnosis or educational level, were considered as potential modifiers of the effect of the intervention.

The intervention primarily targeted the knowledge deficits of cancer survivors regarding SPC screening, but also provided encouraging messages and information on screening participation to address attitudes and self-efficacy. We adopted a photo-novel format for the intervention because of the low understanding of SPC among the study population [11] and because this format fit our social cognitive model framework and our purpose to increase interest among cancer survivors. [13]

The development process of the photo-novel followed the National Cancer Institute guidelines for designing printed educational materials for screening [13]. The printed material was 21 pages in length and featured colorful graphics and easy-to-read text. It was designed to enable 100% comprehension at the 10^th^ grade level and used amateur actors instead of real cancer survivors. The material included a 17-page photo-novel with the story of a female breast cancer survivor who attended a support group meeting and heard from a friend that another survivor was recently diagnosed with colorectal cancer. The main character was confused about the nature of the new cancer and thought that it could be a breast cancer metastasis, but the friend clarified that it was a newly developed colorectal cancer. The main character then began to worry about the occurrence of a new cancer and consulted her physician to find out how to address this problem. The physician explained the definition of SPC and recommended routine screening for other common cancers. The main character then got routine cancer screening tests and was reassured by the physician. The printed materials also included brief summaries of the following topics: 1) the distinction between SPC and recurrence or metastasis; 2) the risk of developing cancer in survivors compared to the general population; 3) the early detection of SPC; 4) the prevention of SPC; and 5) information on the Korean National Cancer Screening Program. [14]

Patients in the attention control group received an educational material on the use of health supplement products. The material was almost the same design, format, and graphics as the intervention materials, but differed in the contents. A female breast cancer survivor who attended a support group meeting and heard from a friend about a health supplement which was good for cancer survivors. The main character was confused about the safety and effectiveness of the supplement. She thought that it was o.k. to take as it was made of natural herb, but a friend of her recommend her to reconsider it. Then the main character consulted her physician to find out whether to take the supplement or not. The physician explained the definition of health supplements and recommended health diet instead of taking supplements. In addition, he explained the patient how to choose health supplement such as ingredients, side effects, and safety. The printed materials also included brief summaries about how to take health supplement.

### Study procedures

All participants completed a self-administered pre-intervention survey and received the corresponding intervention materials. A pre-intervention test evaluated their knowledge, attitudes, and practice regarding SPC screening. Details of the baseline measurement are available elsewhere. [15]

Two weeks after receiving the intervention materials, we conducted phone interviews with study participants to evaluate knowledge and attitudes regarding SPC screening. After up to three attempts to contact participants, we reached 134 (81.7%) survivors in the intervention group and 127 (78.4%) survivors in the attention control group.

One year after the initial contact, we performed a telephone interview to assess SPC screening practice. We could reach 142 (86.6%) survivors in the intervention group and 144 (89.25) survivors in the attention control group. After the final interview, we mailed the intervention material on SPC screening to the attention control group, and the control material on health supplement use to the intervention group (**Figure 1**).

### Study outcomes and measures

The primary outcome measure of the study was completion of all appropriate screening within 2 years for cancers other than the survivor's primary cancer. [15] Specific criteria were defined considering the National Cancer Screening Program in Korea, [14] the cancer screening guidelines in Korea, [16], [17], [18], [19] epidemiological evidence from cancer survivors [2], [20] and from Asian populations, [12], [21], [22] and current cancer screening practices in Korea [23] (**Table S2**). Screening tests aimed at detecting the specific primary cancer for each cancer survivor were excluded from each calculation. [8], [15]

Questions on screening practices addressed whether individuals had ever had exams for breast cancer (mammogram or breast sonography), stomach cancer (endoscopy or upper gastrointestinal series), cervical cancer (Papanicolaou test) or colorectal cancer (fecal occult blood test, sigmoidoscopy, colonoscopy or barium enema). As patients had difficulties with the description of different screening tests, [24] the questions included relevant explanations and were simplified so that patients did not have to identify the specific type of screening test (e.g., we asked “Have you ever had a stomach screening test? Stomach cancer screening tests include gastrofibroscopy or upper gastrointestinal series”). At the baseline survey, the patients were also provided with relevant pictures (e.g., patients getting gastrofibroscopy). A positive answer to any screening question was followed by questions about the timing of the most recent exam (less than 1 year, 1–2 years, 2–5 years, >5 years, or none).

The secondary outcome measures were the knowledge and attitude regarding SPC screening at the 2-week interview (**Table S3**). The questionnaires on knowledge and attitudes were developed based on our qualitative studies and were pre-tested on 5 survivors. The knowledge questionnaire included 5 true-false questions covering: 1) occurrence of SPC; 2) difference between ‘routine surveillance test’ and ‘second cancer screening’; 3) cancer screening needs and recommendations for cancer survivors; 4) risk of developing SPC; and 5) meaning of routine surveillance tests (blood test and chest X-ray). Correct answers were given 1 point, and “Don't know” responses were treated as incorrect. The Cronbach α for the baseline assessment of knowledge was 0.23, reflecting the heterogeneity of the items [25] and the lack of familiarity of survivors with the topic [26]. The scores for all questions on attitude were averaged for each participant.

The attitude questionnaire included six questions on: 1) needs for cancer screening; 2) intention to have screening; 3) intention to have screening following physicians' recommendation; 4) perceived health benefits; 5) perceived benefits for the family; and 6) perceived benefits of cancer screening. Responses were recorded on 4-point scale (strongly agree = 3, agree = 2, disagree = 1, strongly disagree = 0) and the scores for all questions on attitude were averaged for each participant. The Cronbach α for the baseline assessment was 0.81.

In addition, we also conducted a brief process evaluation among participants two weeks after the intervention. Intervention exposure was assessed by asking whether the respondent had looked at the material. Potential responses were ‘did not have a look at all’, ‘browsed it quickly’, ‘read it through’, and ‘read it carefully in detail’. Follow-up interviewers were not blinded to intervention assignment.

### Sample size and statistical analysis

Based on published screening rates, [9], [27] we assumed the SPC screening completion rate in the cancer survivors would be approximately 40%. We determined that 326 subjects would be required to detect an absolute difference of 20% in completion of all appropriate screening with a power of 90%, a two-tailed α of 0.05, and 20% of losses to follow-up.

We compared the baseline characteristics of study participants in the intervention and control groups using χ^2^ tests for categorical variables and t-tests for continuous variables. All analysis of study outcomes were performed by intention-to-treat. We compared the primary outcome (i.e. screening behavior) in the intervention and control groups using χ^2^ tests and secondary outcome measures (i.e. knowledge and attitudes) using t-tests. A two-sided P value of <0.05 was used to determine statistical significance. All analyses were conducted using SAS 9.1.3 statistical software (SAS institute, Cary, NC).

## Results

### Sample characteristics

The baseline characteristics of the survivors in the control and intervention groups are presented in **Table 1**. Although the two groups were similar at baseline, survivors in the intervention group were significantly more likely to be married than those in the control (92.9% vs. 85.0%). The two groups did not differ significantly on baseline knowledge, attitudes, and behaviors regarding cancer screening (**Table 2**). At the 2-week and 1-year follow-ups there were no differences in drop-out rates between the intervention and the attention control groups (data not shown).

**Table 1:** Demographic and clinical characteristics of study participants at baseline.

Characteristic	Intervention(n = 164)	Control(n = 162)	P-value
Age, years (mean, SD)	56.4, 9.6	57.5, 10.3	0.32
Sex			
Female	92 (56.1)	102 (63.0)	0.21
Male	72 (43.9)	60 (37.0)	
Marital status			
Single/Divorced/Separated	12 (7.4)	24 (15.0)	0.03
Married	151 (92.6)	136 (85.0)	
Level of education			
<12 years	46 (28.2)	41 (25.6)	0.60
≥12 years	117 (71.8)	119 (74.4)	
Monthly income (KRW)			
<3,000,000	85 (53.1)	86 (54.8)	0.77
≥3,000,000	75 (46.9)	71 (45.2)	
Employment			
Unemployed	88 (54.0)	100 (62.5)	0.12
Employed	75 (46.0)	60 (37.5)	
Time since diagnosis, years (mean, SD)	3.3 (2.0)	3.4 (2.1)	1.00
1–2 year	56 (34.1)	57 (35.2)	0.84
>2 years	197 (65.9)	105 (64.8)	
Cancer type			
Stomach	31 (18.9)	30 (18.5)	0.99
Breast	42 (25.6)	43 (26.5)	
Colon	60 (36.6)	60 (37.0)	
Thyroid	31 (18.9)	29 (17.9)	
Stage			
I	76 (46.3)	69 (42.6)	0.79
II	40 (24.4)	42 (25.9)	
III	48 (29.3)	51 (31.5)	
Comorbidities			
Yes (≥1)	91 (55.5)	93 (57.8)	0.68
No	73 (44.5)	68 (42.2)	
Current smoking			
Yes	8 (5.0)	4 (2.6)	0.26
No	153 (95.0)	152 (97.4)	
Current drinking			
Yes	37 (22.7)	29 (18.2)	
No	126 (77.3)	130 (81.8)	0.32
Perceived health			
Good, Very good	66 (40.2)	72 (45.6)	
Very poor, Poor, Fair	98 (59.8)	86 (54.4)	0.33

Values in the table are number (percentage) unless otherwise indicated.

S.D.: Standard deviation; KRW: Korean Won.

P-values from t-tests for continuous variables or χ^2^ tests for categorical variables.

**Table 2:** Knowledge, attitudes, and screening practices of study participants at baseline.

Characteristics	Intervention(n = 164)	Control(n = 162)	P-value
Knowledge and attitudes at baseline			
Knowledge, mean (SD)	0.75 (0.19)	0.74 (0.22)	0.89
Attitude, mean (SD)	2.68 (0.43)	2.67 (0.40)	0.69
Screening practice at baseline			
Gastric cancer screening (n = 265), N (%)	80 (60.2)	87 (65.9)	0.33
Breast cancer screening (n = 109), N (%)	46 (72.0)	32 (54.2)	0.06
Colon cancer screening (n = 206), N (%)	35 (33.7)	37 (36.3)	0.69
Cervix cancer screening (n = 194), N (%)	58 (64.1)	68 (66.7)	0.71
Completion, N (%)	64 (39.0)	59 (36.4)	0.63

Values in the table are number (percentage) unless otherwise indicated.

S.D.: Standard deviation.

Completion: completion of all appropriate second cancer screening in the last 2 years (for details, please see text).

P-values from t-tests for continuous variables or χ^2^ tests for categorical variables.

### Process evaluation

Among 134 survivors in the intervention group who were reached 2 weeks after the intervention, 115 (85.8%) replied that they had read the material. Most of them found the material easy to follow (70.4%), recommendable to other survivors (61.7%), and helpful or very helpful for planning future cancer screening (88.6%) (**Table 3**).

**Table 3:** Process evaluation of study intervention.

Among all who were reached 2 weeks after the intervention	Intervention(n = 134)	Control(n = 127)	P-value
Have you read the material?			0.94
No	19 (14.2)	21 (16.5)	
Browsed it	3 (2.2)	2 (1.6)	
Read it through	52 (38.8)	48 (37.8)	
Read it carefully in detail	60 (44.7)	56 (44.1)	

Among those who read the material	Intervention(n = 115)	Control(n = 106)	Group differenceP-value
Was the material easy to understand?			0.89
Easy to follow	81 (70.4)	77 (72.6)	
Intermediate difficulty	28 (24.4)	23 (21.7)	
Hard to understand	6 (5.2)	6 (5.7)	
Would you recommend the material to other survivors?			0.22
Yes	71 (61.7)	57 (53.8)	
Uncertain	28 (24.4)	37 (34.9)	
No	16 (13.9)	12 (11.3)	
Was it helpful to plan your future cancer screening?			NA
Very helpful	25 (21.9)		
Helpful	76 (66.7)		
Somewhat helpful	12 (10.5)		
Not helpful at all	1 (0.9)		

P-values from χ^2^ tests or Fisher's exact tests when indicated.

### Efficacy of the intervention

After 2 weeks, the knowledge score in the intervention group was significantly higher than in the control group (mean [SD] 0.81 (0.18) vs. 0.75 (0.21); P<0.01), while there was no significant difference in attitude score (mean [SD] 2.64 (0.38) vs. 2.57 (0.46); P = 0.18). After 1 year of follow-up, completion of all appropriate cancer screening within 2 years was 47.2% in both intervention (67/142) and control groups (68/144). There was also no significant difference between two groups for each screening test (**Table 4**). Sensitivity analysis which excluded those who completed all appropriate screening within 1 year at baseline (76/326, 23.3%) did not affect the results (data not shown). Per-protocol analysis limited to patients who had read the material through or carefully generated similar results (data not shown). A pre-post comparison of screening rates showed significant increase in the completion of all appropriate cancer screening from baseline to one year of follow-up (39.5% vs. 47.2%; P = 0.02), primarily due to an increase in colorectal cancer screening (37.2% to 48.3%; P<0.01) (**Table 5**).

**Table 4:** Comparison of outcomes between intervention and control group.

Characteristics	Intervention	Control	P-value
Knowledge and attitudes at 2 weeks after intervention	(n = 134)	(n = 127)	
Knowledge, mean (SD)	0.81 (0.18)	0.75 (0.22)	<0.01
Attitude, mean (SD)	2.64 (0.38)	2.57 (0.46)	0.18
Screening practice at 1 year after intervention	(n = 142)	(n = 144_	
Gastric cancer screening (n = 233), N (%)	77 (67.5)	80 (67.2)	0.96
Breast cancer screening (n = 94), N (%)	32 (72.7)	28 (56.0)	0.09
Colon cancer screening (n = 180), N(%)	42 (47.2)	45 (49.5)	0.76
Cervix cancer screening (n = 168), N (%)	52 (66.7)	60 (66.7)	1.00
Completion (n = 286), N(%)	67 (47.2)	68 (47.2)	1.00

S.D.: Standard deviation.

Completion: completion of all appropriate second cancer screening in the last 2 years (for details, please see text).

P-values from t-tests for continuous variables or χ^2^ tests for categorical variables.

**Table 5:** Change in the SPC screening behavior in the intervention and control group.

	BaselineN (%)	After 1 yearN (%)	P-value
Gastric cancer screening			
All (N = 233)	150 (64.4)	157 (67.4)	0.49
Intervention group (N = 118)	72 (63.2)	77 (67.5)	0.42
Control group (N = 119)	78 (65.5)	80 (67.2)	0.85
Breast cancer screening			
All (N = 94)	59 (62.8)	60 (63.8)	1.00
Intervention group (N = 44)	32 (72.7)	32 (72.7)	1.00
Control group (N = 50)	27 (54.0)	28 (56.0)	1.00
Colon cancer screening			
All (N = 180)	67 (37.2)	87 (48.3)	<0.01
Intervention group (N = 89)	33 (37.1)	42 (47.2)	0.08
Control group (N = 91)	34 (37.4)	45 (49.5)	0.05
Cervix cancer screening			
All (N = 168)	112 (66.7)	112 (66.7)	1.00
Intervention group (N = 78)	51 (65.4)	52 (66.7)	1.00
Control group (N = 90)	61 (67.8)	60 (66.7)	1.00
All appropriate cancer screening			
All (N = 286)	113 (39.5)	135 (47.2)	0.02
Intervention group (N = 142)	58 (40.8)	67 (47.2)	0.18
Control group (N = 155)	55 (38.2)	68 (47.2)	0.07

P-value for difference between baseline and 1 year by McNemar tests for matched sample.

## Discussion

To our knowledge, this is the first trial targeted to this specific population. We found that the educatinal material was effective for increasing knowledge of SPC screening. However, it did not promote cancer screening practice among cancer survivors. The strengths of our study include the development of educational materials based on previous qualitative studies and theoretical models, the use of an attention control design to reduce attention biases, and the low attrition rate.

Distributing a photo-novel educational material during clinic visits is an attractive strategy because it can be broadly implemented at low cost. This strategy, however, was found ineffective for increasing SPC screening rates. There might be several reasons for the lack of efficacy. First, our simple intervention targeted at increasing knowledge may be insufficient to achieve a measurable impact on SPC screening. Although we observed a small but significant effect on knowledge, a single educational material unaccompanied by other supporting and reinforcing components constitutes a weak stimulus, probably insufficient for changing complex behavior. [13] Survivors did not discuss the educational materials with health care professionals (eg, health educators) in face-to-face or telephone interviews, which could have provided opportunities to increase knowledge, ask questions, reinforce their motivation, and facilitate the actual uptake process. Our findings are consistent with a recent systematic review of the US Agency for Healthcare Research and Quality which found that small media interventions, defined as print or video educational materials mailed or provided without decision aids, generally have not been effective in improving cancer screening rates. [28]

Second, our intervention did not incorporate logistical support, such as direct provision of services or personalized guidance for actual screening. Our recent qualitative study showed that oncologists are not knowledgeable about SPC screening and they do not directly provide screening tests to their patients (unpublished data). In addition, although Korea has a national cancer screening program, patients have to arrange their own tests as there is no designated primary care provider to arrange cancer screening in Korea. So, to be successful our intervention may require additional patient actions that may need further support and logistical organization. [29]

Third, our results also suggest the possibility of contamination in the attention control group as the screening rate increased during the trial period regardless of the group assignment. Survivors in the control group may have had the opportunity to think about second cancers through administration of study questionnaires at baseline and at two weeks, and may have opted for further cancer screening even without complete knowledge of SPC screening. Media coverage of SPC risk in cancer survivors during the study period based on a previous epidemiological study in Korea [2] may have been another potential source of contamination.

There are some limitations. The assessment of cancer screening practices was based on participants' self-report, which may be subject to over- or underreporting [30]. Surveys are the most common methods for monitoring screening compliance [30], and we used carefully phrased questions to maximize accuracy without requiring survivors to distinguish among similar tests. In addition, we conducted the study at two major cancer centers in the Seoul metropolitan area, and the results may not be generalized to all Korean cancer survivors who may be less educated or living in other communities.

As a first study of its kind in this population, our study provides important insights into the efficacy of interventions aimed at increasing SPC screening and suggests the need for more resource-intensive strategies to actively engage survivors and encourage SPC screening. Promising alternatives which have been used with some success in promoting cancer screening include one-on-one [31] or group education, [32] telephone outreach, [33] and patient navigators. [34] The lack of efficacy of our patient-based intervention also suggests the need for future research into system-level interventions (eg, using nonclinicians to support screening [35]) or provider-directed approaches (eg, physician reminders [36], [37] or performance feedback [38]). In addition to SPC, cancer survivors face multiple challenges after treatment of the primary cancer and discordant expectations with respect to the roles of oncologists and primary care physicians can lead to deficiencies in clinical care after completion of treatment for the primary cancer [39]. Clarifying the roles and responsibilities of the different levels of care, providing navigation support for the patient, shared care, [40] and routine visit to primary care physicians [10], [41] could be potential approaches to improve SPC screening in this population.

In conclusion, this study does not support the use of simple educational materials to increase SPC screening practices among cancer survivors, although we observed a small but significant improvement in SPC knowledge. Given the low rates of SPC screening, more intensive interventions for patients and physicians and system-levels interventions to facilitate SPC screening need to be explored and tested among cancer survivors.

## Supporting Information

Table S1 Incidence and 5 year relative survival rates of four cancers in Korea.
(DOC)

Table S2 Korean recommendations and operational definition of appropriate cancer screening used in this study.
(DOCX)

Table S3 Items to measure knowledge, attitude, and behavior regarding second cancer screening.
(DOCX)

Protocol S1 Trial Protocol.
(PDF)

Checklist S1 CONSORT Checklist.
(DOC)
