# Supplementary material for: Efficacy of an Educational Material on Second Primary Cancer Screening Practice for Cancer Survivors: A Randomized Controlled Trial
Source: PLoS One. 2012 Mar 29;7(3):e33238. doi: 10.1371/journal.pone.0033238 (PMC3315564; doi:10.1371/journal.pone.0033238)
Supplement: Table S1 — Incidence and 5 year relative survival rates of four cancers in Korea. (DOC) [file pone.0033238.s001.doc]

| Table S1. Incidence and 5 year relative survival rates of four cancers in Korea | | | | | | | |
| --- | --- | --- | --- | --- | --- | --- | --- |
|  | Incidence (Crude)* | | |  | 5 year relative survival^†^ | | |
|  | Male |  | Female |  | Male |  | Female |
| Stomach | 65.5 |  | 26.3 |  | 57.0 |  | 55.1 |
| Colorectal | 39.6 |  | 22.2 |  | 66.7 |  | 62.4 |
| Breast | 0.2 |  | 31.0 |  | 87.1 |  | 87.3 |
| Thyroid | 5.8 |  | 35.0 |  | 95.7 |  | 98.5 |

* per 100,000 persons per year

^†^ percent
