# Supplementary material for: Efficacy of an Educational Material on Second Primary Cancer Screening Practice for Cancer Survivors: A Randomized Controlled Trial
Source: PLoS One. 2012 Mar 29;7(3):e33238. doi: 10.1371/journal.pone.0033238 (PMC3315564; doi:10.1371/journal.pone.0033238)
Supplement: Table S2 — Korean recommendations and operational definition of appropriate cancer screening used in this study. (DOCX) [file pone.0033238.s002.docx]

Table S2. Korean recommendations and operational definition of appropriate cancer screening used in this study

|  | **Stomach cancer screening** | **Colorectal cancer screening** | **Breast cancer screening** | **Cervical cancer screening** |
| --- | --- | --- | --- | --- |
| **National Cancer Screening Guideline**^20^ | - Male and female ≥40 - Every 2 years - Gastrofibroscopy or UGIS | - Male and female ≥50 - Every 5-10 years - Colonoscopy or barium enema and sigmoidoscopy - Earlier and more frequent screening for high risk groups | - Female ≥40 - Every 1-2 years - Mammogram and clinical breast exam | - Female ≥20 - Every year - Papanicolau tests |
| **National Cancer Screening Program**^19^ | - Male and female ≥40 - Every 2 years - Gastrofibroscopy or UGIS | - Male and female ≥50 - Every year - FOBT; if positive, colonoscopy or barium enema follows | - Female ≥40 - Every 2 years - Mammogram and clinical breast exam | - Female ≥30 - Every 2 years - Papanicolau test |
| **Lifetime health promotion for Koreans**^21^ | - Male ≥40, Female ≥50 - Every 2 years | - Male and female ≥50 - FOBT every year, or barium enema or sigmoidoscopy every 5 years, or colonoscopy every 10 years | - Female ≥40 - Every 2 years (40 – 49 years of age) or every 3 years (≥50 years of age) - Mammogram | - Female with history of sexual contact - Every 3 years - Papanicolau test |
| **Screening guideline for seven major cancers**^22^ | - Male and female ≥40 - Every 2 years - Gastrofibroscopy or UGIS | - Male and female ≥50 - Every 5-10 years - Colonoscopy or barium enema and sigmoidoscopy - Earlier and more frequent screening for high risk groups | - Female ≥40 - Every 1-2 years - Mammogram and clinical breast exam | - Female ≥20 - Every year - Papanicolau test |
| **Other considerations** |  | - Korean male cancer survivors have 4.0 times the risk of colorectal cancer than the general population^2^ - Colonoscopy every 3 years has been suggested as the most cost effective strategy for male cancer survivors^24^ | - Korean women have the peak incidence of breast cancer in their forties^15^ - Asian women have high prevalence of dense breasts^25, 26^ - Breast sonography is commonly practiced in combination with or as a replacement of mammography in Korea^26^ |  |
| **Operational definition of appropriate cancer screening used in the current study** | - Within 2 years - Gastrofibroscopy or UGIS | - Within 2 years - FOBT or sigmoidoscopy or colonoscopy or barium enema | - Within 2 years - Mammography or breast sonography | - Within 2 years - Papanicolau test |

UGIS: Upper gastrointestinal series; FOBT: Fecal occult blood testing
