# Supplementary material for: Efficacy of an Educational Material on Second Primary Cancer Screening Practice for Cancer Survivors: A Randomized Controlled Trial
Source: PLoS One. 2012 Mar 29;7(3):e33238. doi: 10.1371/journal.pone.0033238 (PMC3315564; doi:10.1371/journal.pone.0033238)
Supplement: Table S3 — Items to measure knowledge, attitude, and behavior regarding second cancer screening. (DOCX) [file pone.0033238.s003.docx]

Table S3. Items to measure knowledge, attitude, and behavior regarding second cancer screening

| Knowledge on second primary cancer screening |
| --- |
| Q1. Cancer survivors can develop another cancer. (Yes) |
| Q2. If taking routine follow-up tests as recommended, periodic cancer screening is not necessary. (No) |
| Q3. Cancer survivors have to receive at least cancer screening tests recommended to those without cancer. (Yes) |
| Q4. Cancer survivors are higher risk of developing another cancer than those without cancer. (Yes) |
| Q5.Blood test or routine X-ray can detect all health problems.(No) |
| Attitudes on second primary cancer screening |
| Q1. I think cancer patients need to receive screening for another cancer. |
| Q2. I would like to receive screening for another cancer. |
| Q3. If doctor recommend me, I would like to receive screening for another cancer. |
| Q4. If I receive screening for another cancer, it will be good for my family. |
| Q5. If I receive screening for another cancer, it will make me feel that my health is well managed. |
| Q6. Receiving screening for another cancer is the best way to cope with development of another cancer. |
