# Supplementary material for: Efficacy of an Educational Material on Second Primary Cancer Screening Practice for Cancer Survivors: A Randomized Controlled Trial
Source: PLoS One. 2012 Mar 29;7(3):e33238. doi: 10.1371/journal.pone.0033238 (PMC3315564; doi:10.1371/journal.pone.0033238)
Supplement: Protocol S1 — Trial Protocol. (PDF) [file pone.0033238.s004.pdf]

Efficacy of an educational material on second primary cancer screening practice for cancer survivors: A randomized controlled trial

Protocol Number: NCCNCS-09-266

Principal investigator [National Cancer Center]:

Dong Wook Shin, MD, MBA
Chief, Hospice & Palliative care branch,
National Cancer Control Institute,
National Cancer Center
Goyang, Gyeonggi, Korea, Republic of, 410-769

Principal investigator [Samsung Medical Center]:

Juhee Cho, MA, PhD
Cancer Education Center,
Samsung Medical Center
Seoul, Korea, Republic of, 135-710

NATIONAL CANCER CENTER
809 Madu 1-dong, Ilsan-gu, Goyang-si,
Gyeonggi-do, 411-764, Republic of Korea
TEL ;+82-31-920-0001
FAX ;+82-31-920-0002

CONTENTS

1. **Summary of the study**
2. **Objectives**
3. **Background and significance**
4. **Methods**
   - **A. Trial design**
   - **B. Study population - eligibility criteria**
   - **C. Intervention**
   - **D. Outcome**
   - **E. Sample size**
   - **F. Randomization**
   - **G. Statistical Methods**
5. **Human subjects**

1. Summary of the study

Cancer surivors have limited knowledge about second primary cancer (SPC) screening and suboptimal rates of completion of screening practices for SPC. Our objective is to test the efficacy of an educational material on the knowledge, attitudes, and screening practices for SPC among cancer survivors. Randomized, controlled trial among cancer survivors from 6 oncology care outpatient clinics in Korea will be conducted. Patients will be randomized to an intervention or an attention control group. The intervention is a photo-novel, culturally tailored to increase knowledge about SPC screening. Knowledge and attitudes regarding SPC screening will be assessed two weeks after the intervention, and screening practices will be assessed after one year.

2. Objectives

- **Primary objective:** To assess the impact of an educational material regarding second primary cancer screening on screening practice among cancer survivors
- **Secondary objective:** To assess the impact of an educational material on knowledge and attitudes toward second priamry cancer screening among cancer survivors.

3. Background and significance

- Higher incidence of second primary cancer was reported in cancer survivor.
- The standardized incidence ratio for second primary cancer were reported to be 2.3 times higher in male cancer survivors in Korea (Park SM et al, 2006,)

- Cancer screening practice of cancer survivors are reported to vary, but usually less than optimally recommended level Wilkins, 2008)
- The 3rd round of Korean National Health and Nutrition Survey showed that cancer survivors had better screening rates than non-cancer controls, but the absolute screening rates were around 50% meaning screening rates in cancer survivors were still suboptimal (Ministry of Health and Welfare)
- According to the cohort study performed among Korean cancer survivors in 2007, doctors usually focus on the follow-up of primary index cancer, and rarely recommend the screening for second primary cancer (National Cancer Center Report).
- Above study also showed that most cervical, breast, and stomach cancer survivors did not have recommendation for the second primary cancer screening from their doctor. In addition, fear of detecting a new cancer and screening cost are among barriers to second primary cancer screening practice. (National Cancer Center Report).
- According to previous qualitative study, cancer survivors had high interest in their health and were very receptive to their doctors' health recommendation, they frequently had not heard about second primary cancer or screening for it. Lack of knowledge seemed to be the critical barrier for SPC screening in this population, and it was suggested that simple intervention, such as an educational material regarding second primary cancer screening, would be effective.

- Other barriers identified were the high cost of private cancer screening program, and the concern for quality of National Cancer Screening program.
- Considering above study results, we hypothesized that simple intervention, such as an educational material regarding second primary cancer and screening for it, would be effective in increasing the knowledge, attitude, and behaviors regarding second primary cancer screening.

4. Methods

A. Trial design

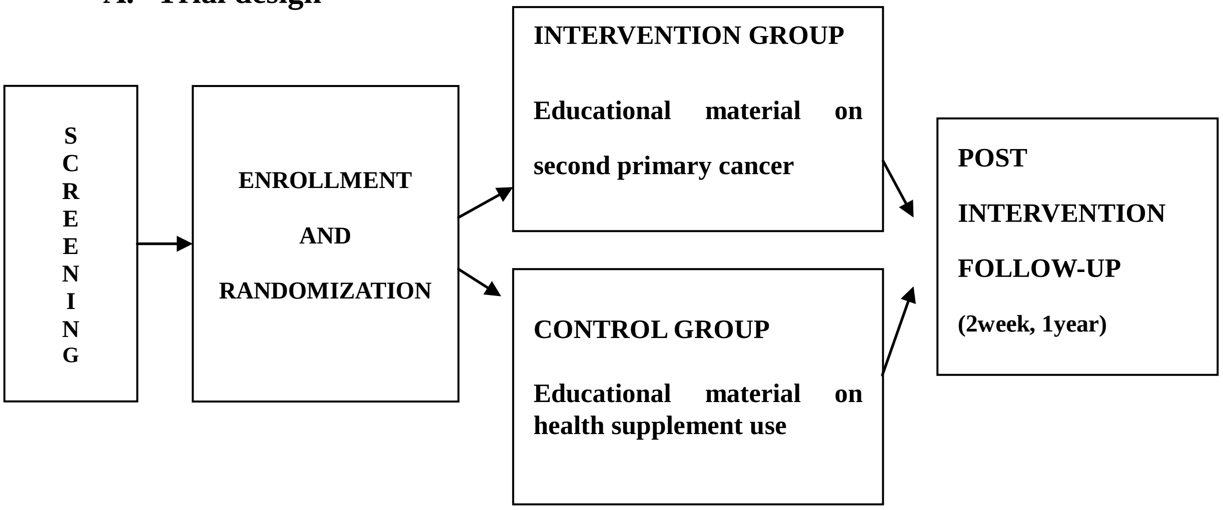

All participants completed a self-administered pre-intervention survey and received the corresponding intervention materials. A pre-intervention test evaluated their knowledge, attitudes, and practice regarding SPC screening.

Two weeks after receiving the intervention materials, we conducted phone interviews with

study participants to evaluate knowledge and attitudes regarding SPC screening. One year after the initial contact, we performed a telephone interview to assess SPC screening practice. After the final interview, we mailed the intervention material on SPC screening to the attention control group, and the control material on health supplement use to the intervention group.

B. Study population - eligibility criteria

Inclusion Criteria:

- Age >=18, <80
- pathologically confirmed cancer diagnosis
- >= 1 year from primary treatment (surgery, radiotherapy, chemotherapy)
- No evidence of recurrence, metastasis, and second primary cancer
- No evidence of hereditary cancer
- ECOG 0,1 or 2
- Ability in reading and understanding Korean

Exclusion Criteria:

- ECOG 3 or 4
- Disagree with the investigators study purpose

C. Intervention

The intervention primarily targets the knowledge deficits of cancer survivors regarding SPC

screening, but also provides encouraging messages and information on screening participation to address attitudes and self-efficacy. We adopt a photo-novel format for the intervention because of the low understanding of SPC among the study population and because this format fits our social cognitive model framework and our purpose to increase interest among cancer survivors.

The development process of the photo-novel follows the National Cancer Institute guidelines for designing printed educational materials for screening. The printed material is 21 pages in length and featured colorful graphics and easy-to-read text. It is designed to enable 100% comprehension at the 10th grade level and use amateur actors instead of real cancer survivors. The material includes a 17-page photo-novel with the story of a female breast cancer survivor who attends a support group meeting and heard from a friend that another survivor is recently diagnosed with colorectal cancer. The main character is confused about the nature of the new cancer and thought that it could be a breast cancer metastasis, but the friend clarifies that it is a newly developed colorectal cancer. The main character then begin to worry about the occurrence of a new cancer and consults her physician to find out how to address this problem. The physician explains the definition of SPC and recommended routine screening for other common cancers. The main character then gets routine cancer screening tests and is reassured by the physician**.** The printed materials also includes brief

summaries of the following topics: 1) the distinction between SPC and recurrence or metastasis; 2) the risk of developing cancer in survivors compared to the general population; 3) the early detection of SPC; 4) the prevention of SPC; and 5) information on the Korean National Cancer Screening Program.

Patients in the attention control group receive an educational material on the use of health supplement products. The material is almost the same design, format, and graphics as the intervention materials, but differed in the contents. A female breast cancer survivor who attends a support group meeting and hear from a friend about a health supplement which is good for cancer survivors. The main character is confused about the safety and effectiveness of the supplement. She thinks that it is o.k. to take as it is made of natural herb, but a friend of her recommend her to reconsider it. Then the main character consults her physician to find out whether to take the supplement or not. The physician explains the definition of health supplements and recommends health diet instead of taking supplements. In addition, he explains the patient how to choose health supplement such as ingredients, side effects, and safety. The printed materials also include brief summaries about how to take health supplement.

Both intervention and control education material are supplemented at the end of the study

protocol.

D. Outcome

The primary outcome measure of the study is completion of all appropriate screening within 2 years for cancers other than the survivor's primary cancer. Specific criteria are defined considering the National Cancer Screening Program in Korea, the cancer screening guidelines in Korea, epidemiological evidence from cancer survivors and from Asian populations, and current cancer screening practices in Korea. Screening tests aim at detecting the specific primary cancer for each cancer survivor is excluded from each calculation.

Questions on screening practices address whether individuals had ever had exams for breast cancer (mammogram or breast sonography), stomach cancer (endoscopy or upper gastrointestinal series), cervical cancer (Papanicolaou test) or colorectal cancer (fecal occult blood test, sigmoidoscopy, colonoscopy or barium enema). As patients have difficulties with the description of different screening tests, the questions include relevant explanations and are simplified so that patients do not have to identify the specific type of screening test (e.g., we asked "Have you ever had a stomach screening test? Stomach cancer screening tests include gastrofibroscopy or upper gastrointestinal series"). At the baseline survey, the patients are also provided with relevant pictures (e.g., patients getting gastrofibroscopy). A

positive answer to any screening question is followed by questions about the timing of the most recent exam (less than 1 year, 1-2 years, 2-5 years, >5 years, or none).

The secondary outcome measures are the knowledge and attitude regarding SPC screening at the 2-week interview. The questionnaires on knowledge and attitudes are developed based on our qualitative studies and are pre-tested on 5 survivors. The knowledge questionnaire included 5 true-false questions covering: 1) occurrence of SPC; 2) difference between 'routine surveillance test' and 'second cancer screening'; 3) cancer screening needs and recommendations for cancer survivors; 4) risk of developing SPC; and 5) meaning of routine surveillance tests (blood test and chest X-ray). Correct answers are given 1 point, and "Don't know" responses are treated as incorrect. The Cronbach α for the baseline assessment of knowledge is 0.23, reflecting the heterogeneity of the items and the lack of familiarity of survivors with the topic. The scores for all questions on attitude are averaged for each participant.

The attitude questionnaire includes six questions on: 1) needs for cancer screening; 2) intention to have screening; 3) intention to have screening following physicians' recommendation; 4) perceived health benefits; 5) perceived benefits for the family; and 6) perceived benefits of cancer screening. Responses are recorded on 4-point scale (strongly

agree = 3, agree = 2, disagree = 1, strongly disagree = 0) and the scores for all questions on attitude are averaged for each participant.

In addition, we also conduct a brief process evaluation among participants two weeks after the intervention. Intervention exposure is assessed by asking whether the respondent have looked at the material. Potential responses are 'did not have a look at all', 'browsed it quickly', 'read it through', and 'read it carefully in detail'. Follow-up interviewers are not blinded to intervention assignment.

E. Sample size

Based on published screening rates, we assume the SPC screening completion rate in the cancer survivors would be approximately 40%. We determined that 326 subjects would be required to detect an absolute difference of 20% in completion of all appropriate screening with a power of 90%, a two-tailed α of 0.05, and 20% of losses to follow-up.

F. Randomization

Eligible cancer survivors will be approached during their regular follow-up visits, and subjects who agreed to participate in the study will be individually randomized to an

intervention or an attention control group according to randomization tables which was generated using a randomization program stratified by oncologist with blocks of size four.

G. Statistical Methods

We compare the baseline characteristics of study participants in the intervention and control groups using χ^2 tests for categorical variables and t-tests for continuous variables. All analysis of study outcomes are performed by intention-to-treat. We compare the primary outcome (i.e. screening behavior) in the intervention and control groups using χ^2 tests and secondary outcome measures (i.e. knowledge and attitudes) using t-tests. A two-sided P value of <0.05 is used to determine statistical significance. All analyses are conducted using SAS 9.1.3 statistical software (SAS institute, Cary, NC).

5. Human subjects

Individual participants in this study gave written informed consent. The study protocol was approved by the Institutional Review Board of National Cancer Center and Samsung Medical Center. All participants signed a written informed consent.

암을 한 번 겪으신 분들을 위한 안내서

더욱 소중한 내 몸을 위한 아름다운 실천

▶ 정기적인 암검진으로 이차암 조기 발견 ◀

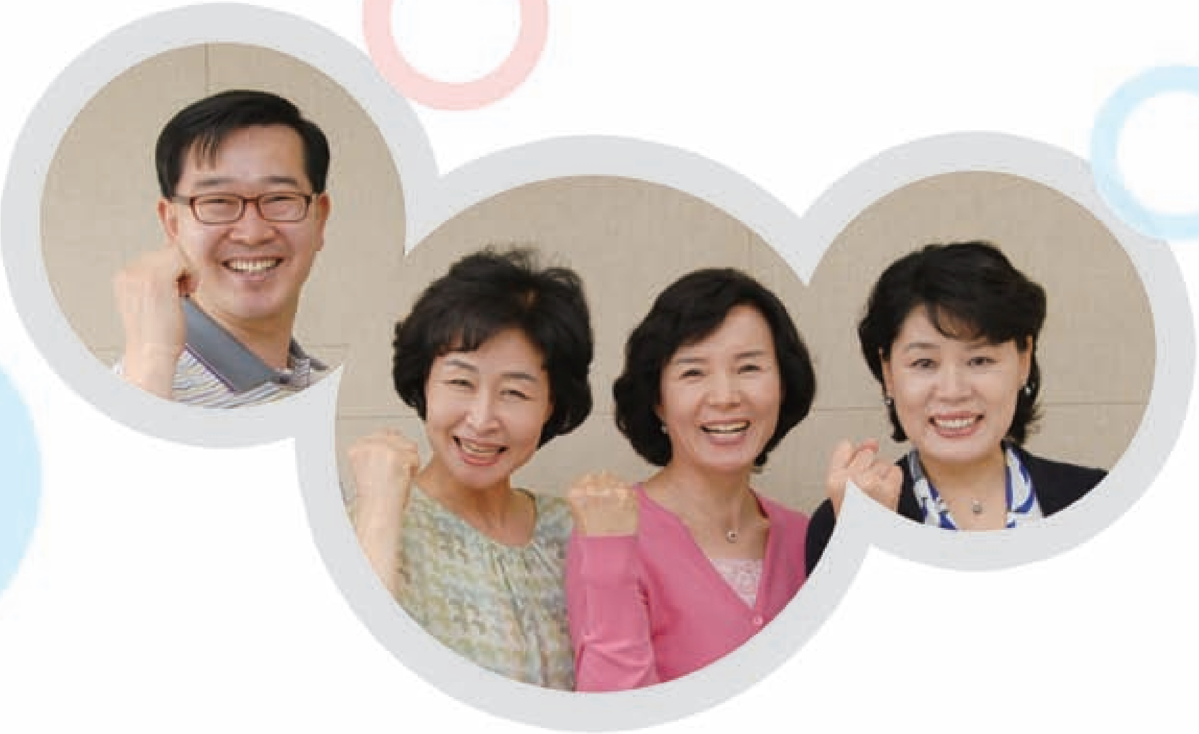

배경 설명

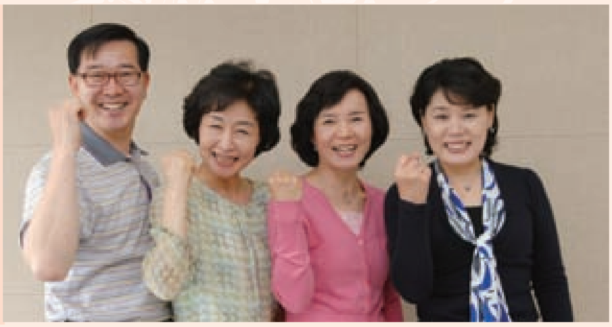

목 적 본 교육자료의 목적은 암을 한번 겪으셨던 분들에게 이차암이 발생할 수 있음을 알려드리고, 이에 대한 적절한 대처 방법을 전달하기 위함입니다.

줄 거 리 유방암 환자인 지혜 엄마는, 환우 모임에서 알고 지내던 유방암 환우인 유미엄마가 이차암으로 대장암이 생겼다는 이야기를 듣고 고민에 빠지게 된다.

등장인물

지혜엄마, 유방암 환자 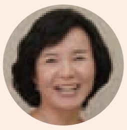

지혜아빠 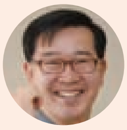

유방암 환자 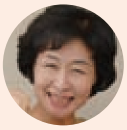 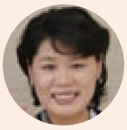

지혜엄마 주치의 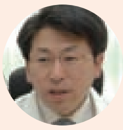

지혜엄마는 한 달에 한 번, 유방암 환우 모임에 나간다.

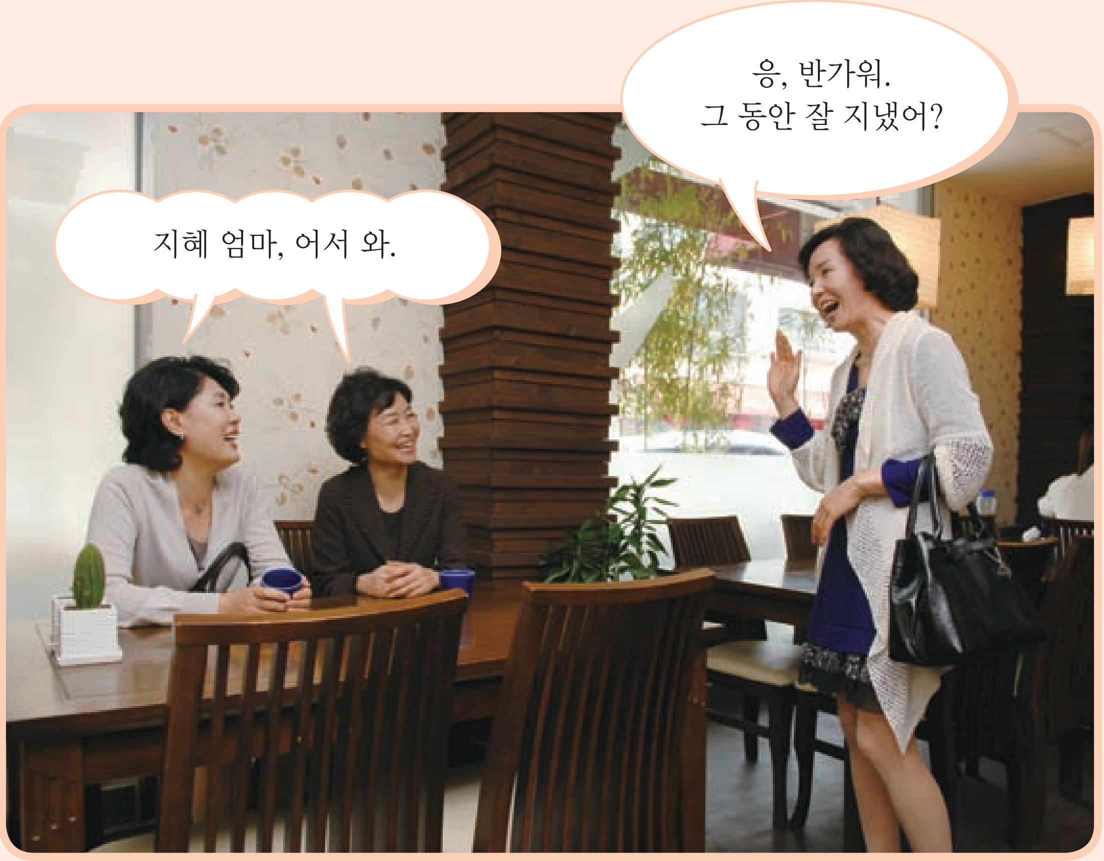

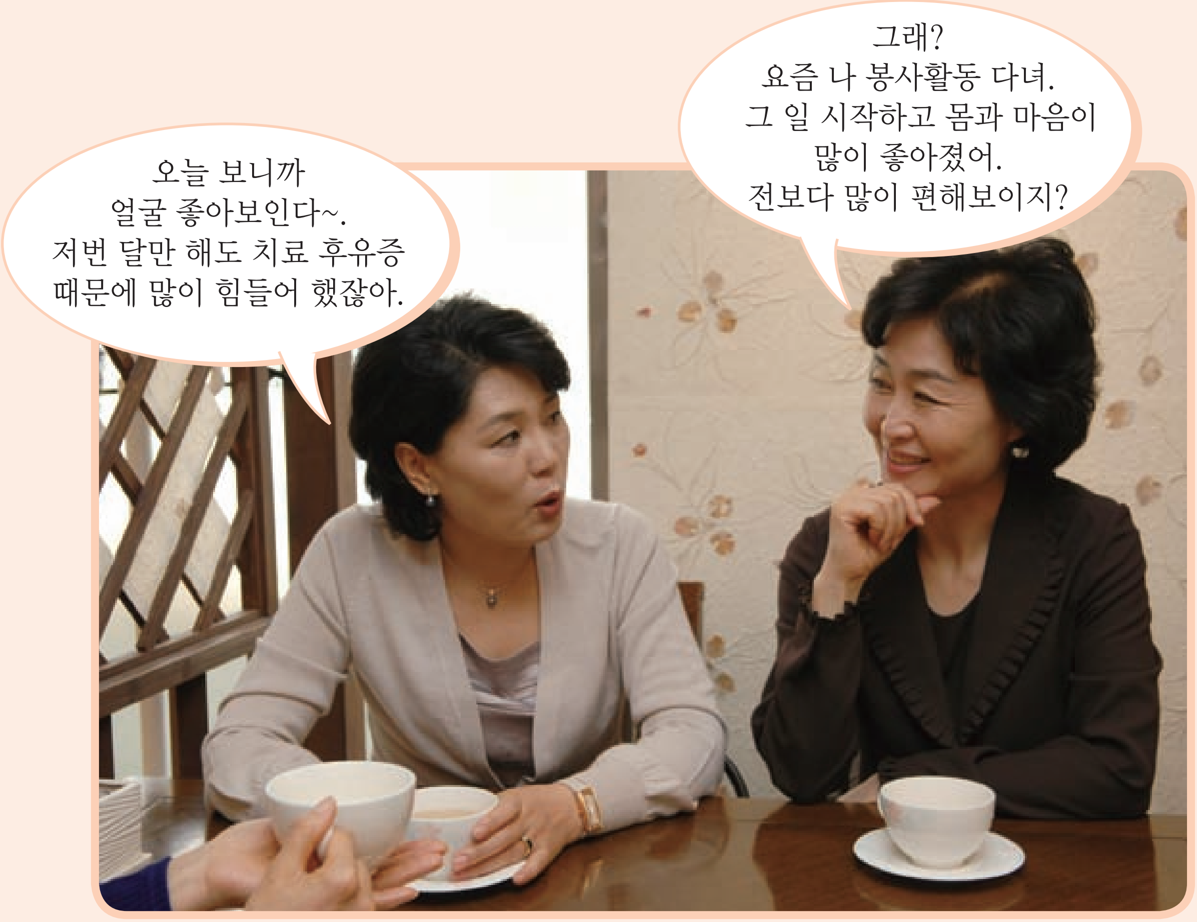

이야기 중, 모임에 안 나온 다른 환우의 소식을 듣게 된다.

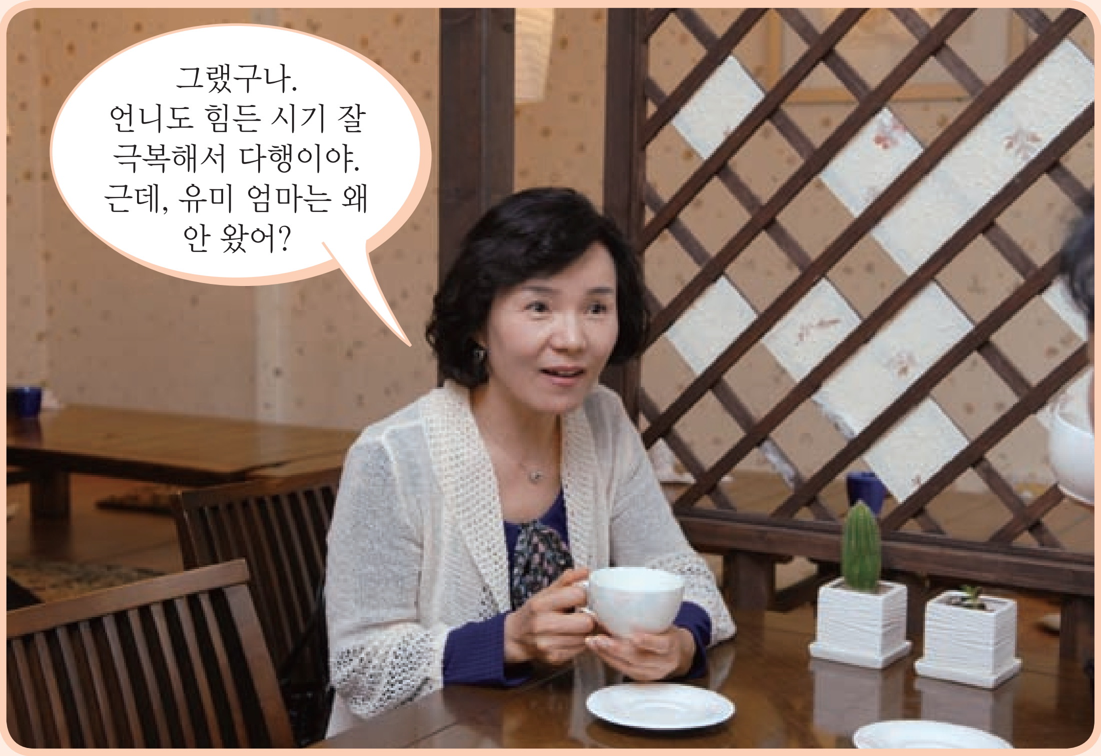

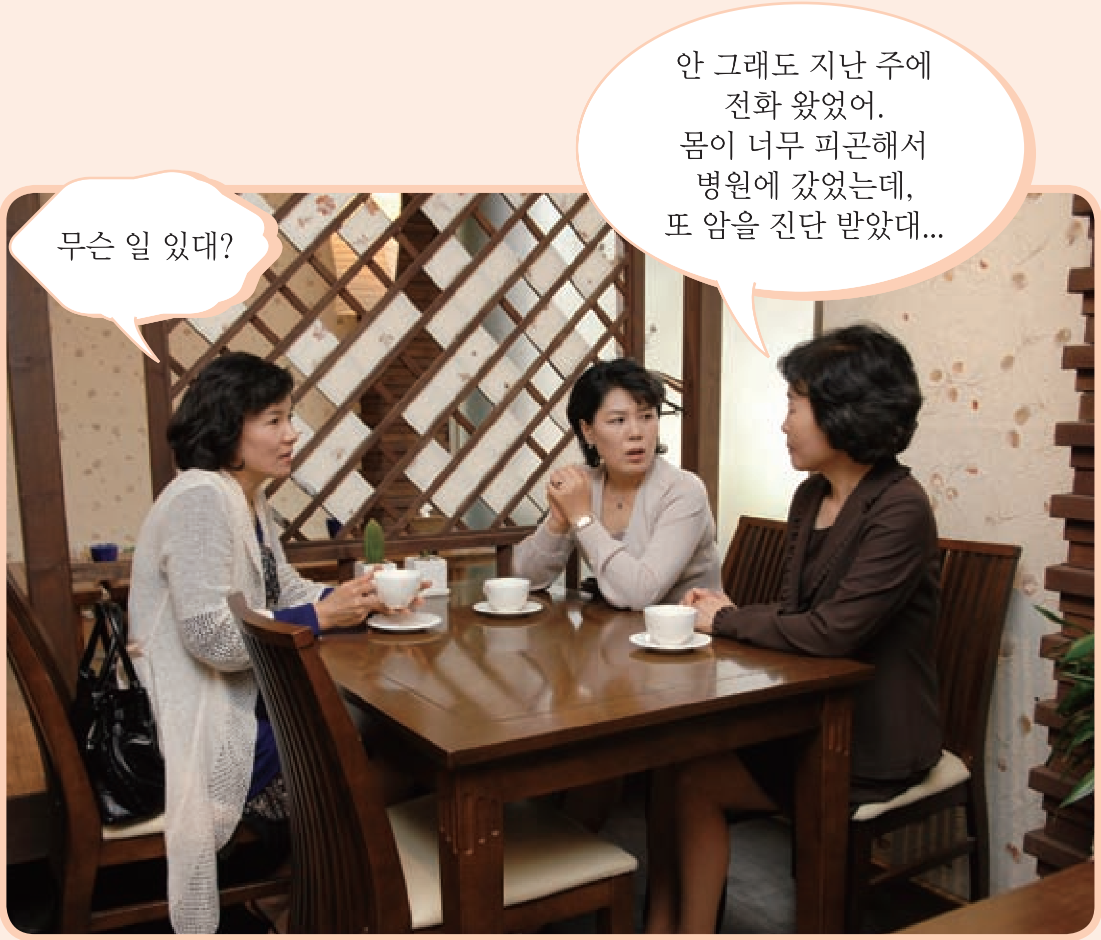

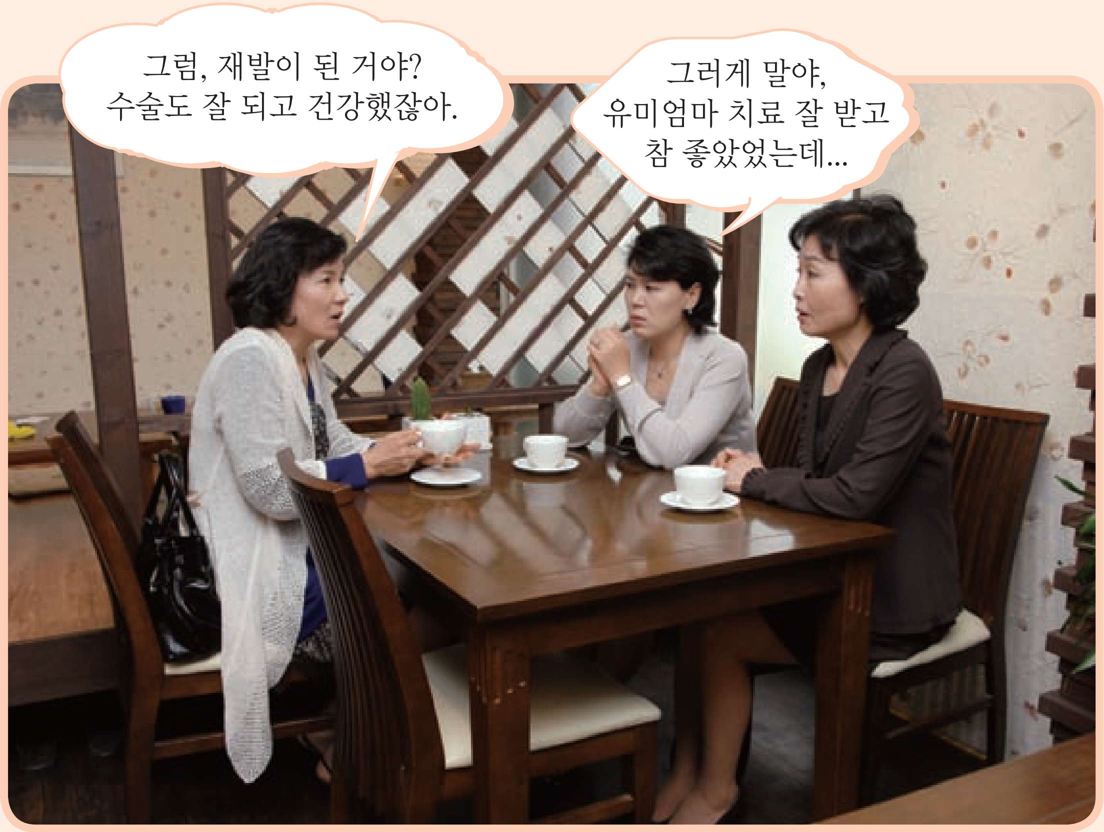
그럼, 재발이 된 거야?
수술도 잘 되고 건강했잖아.
그러게 말야,
유미엄마 치료 잘 받고
참 좋았었는데...

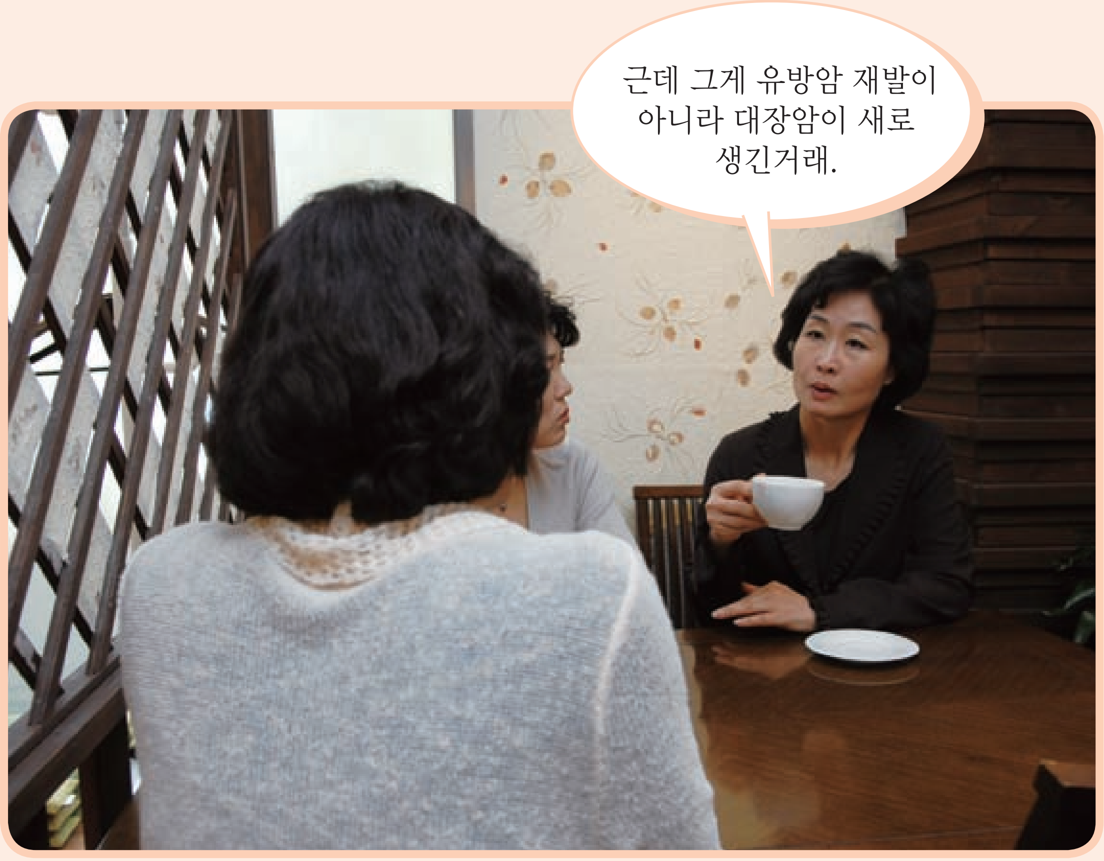
근데 그게 유방암 재발이
아니라 대장암이 새로
생긴거래.

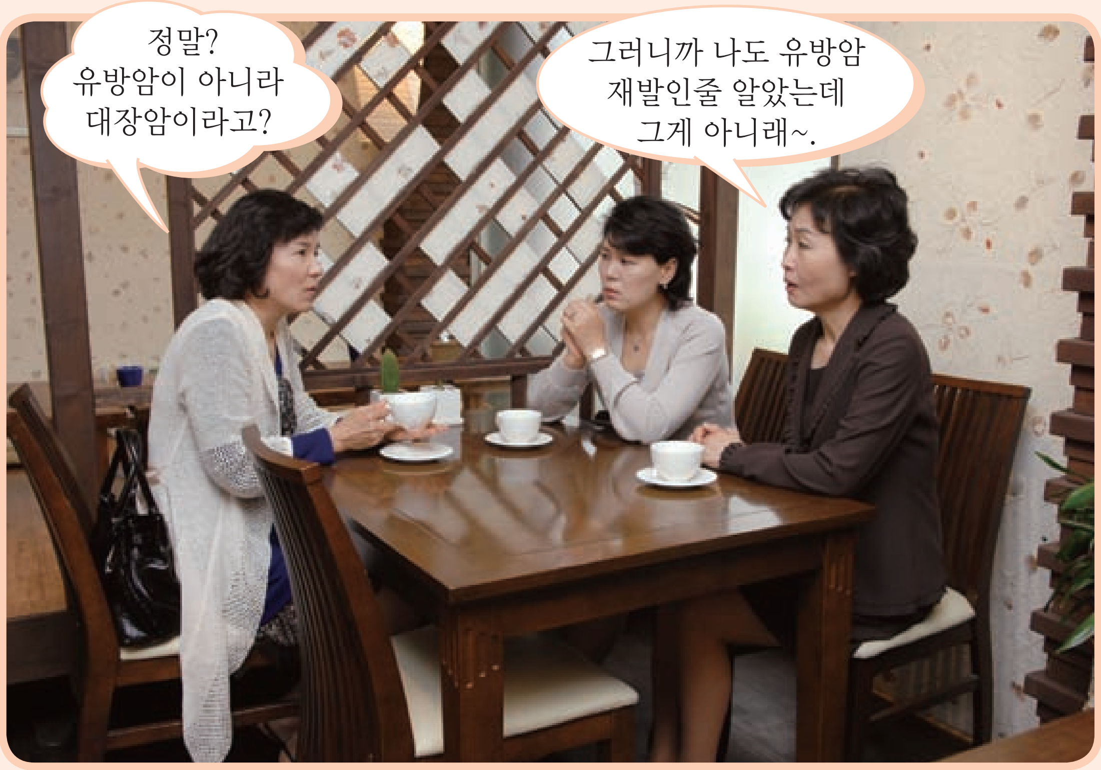
정말?
유방암이 아니라
대장암이라고?
그러니까 나도 유방암
재발인줄 알았는데
그게 아니래~.

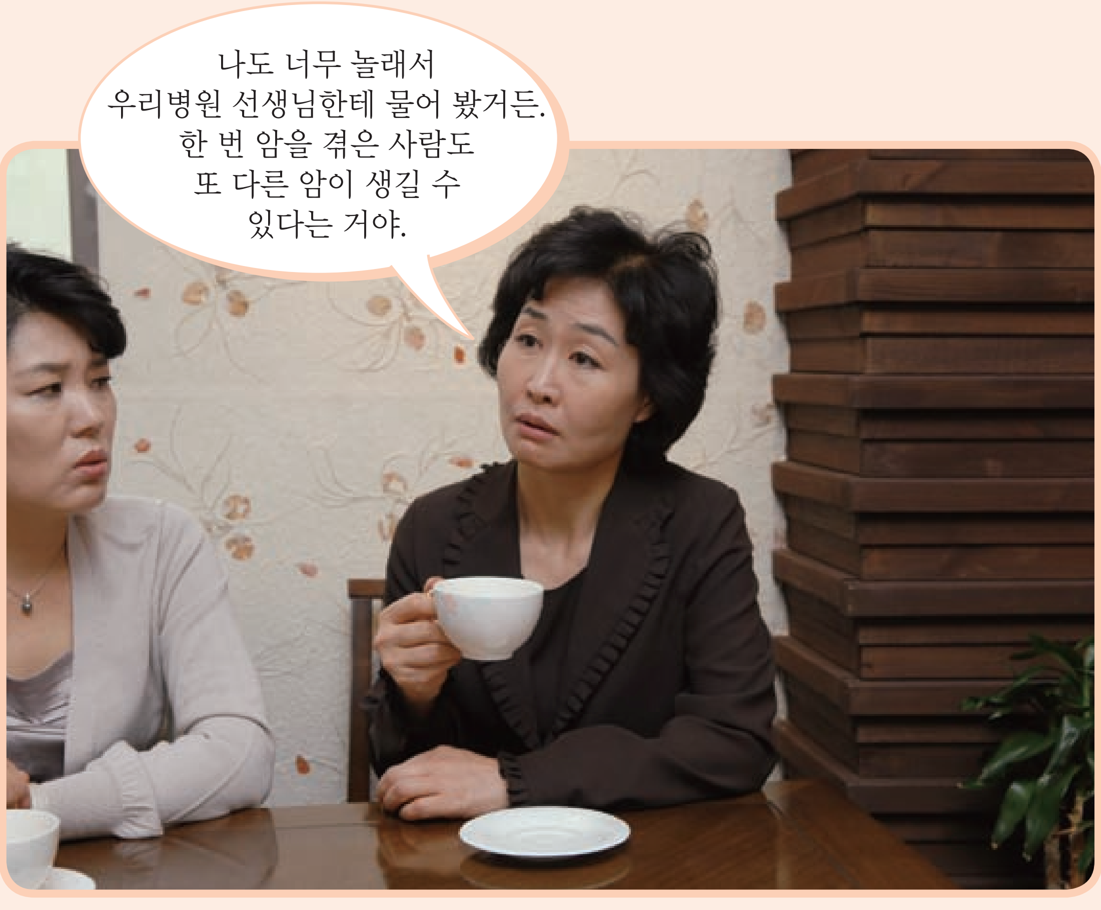
나도 너무 놀래서
우리병원 선생님한테 물어 봤거든.
한 번 암을 겪은 사람도
또 다른 암이 생길 수
있다는 거야.

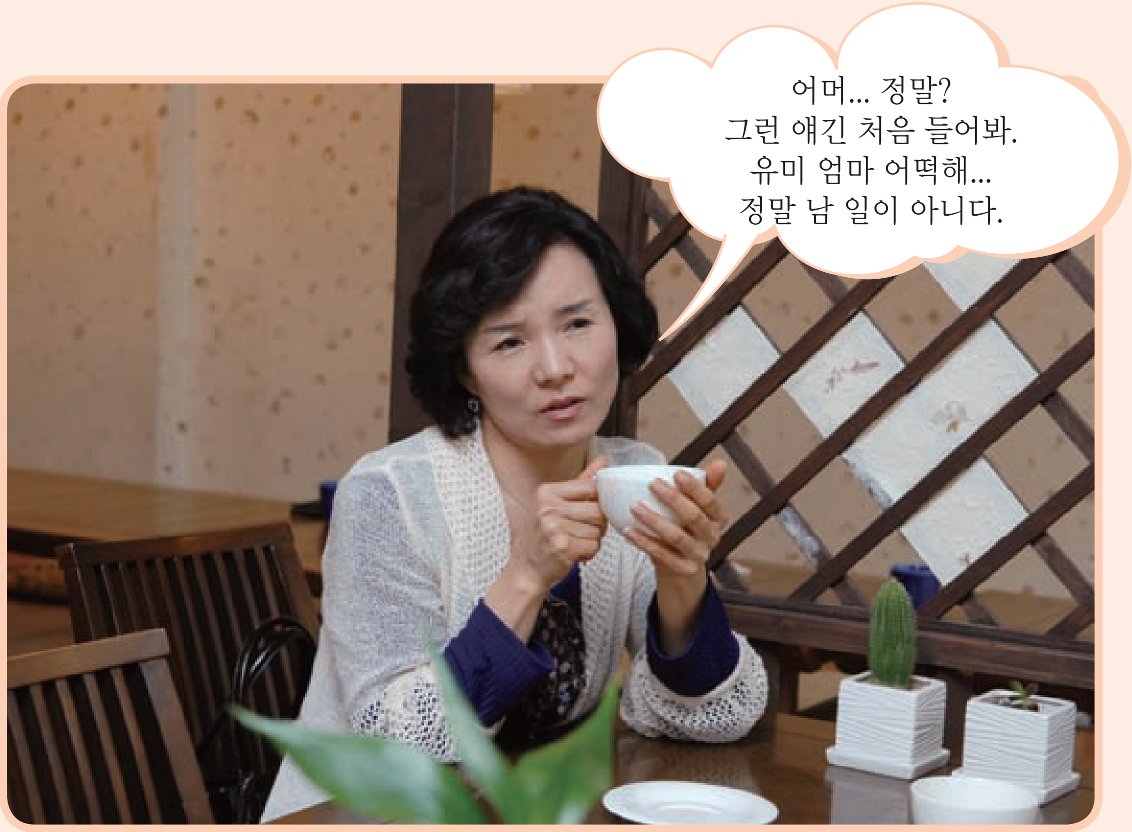

모임이 끝난 후...

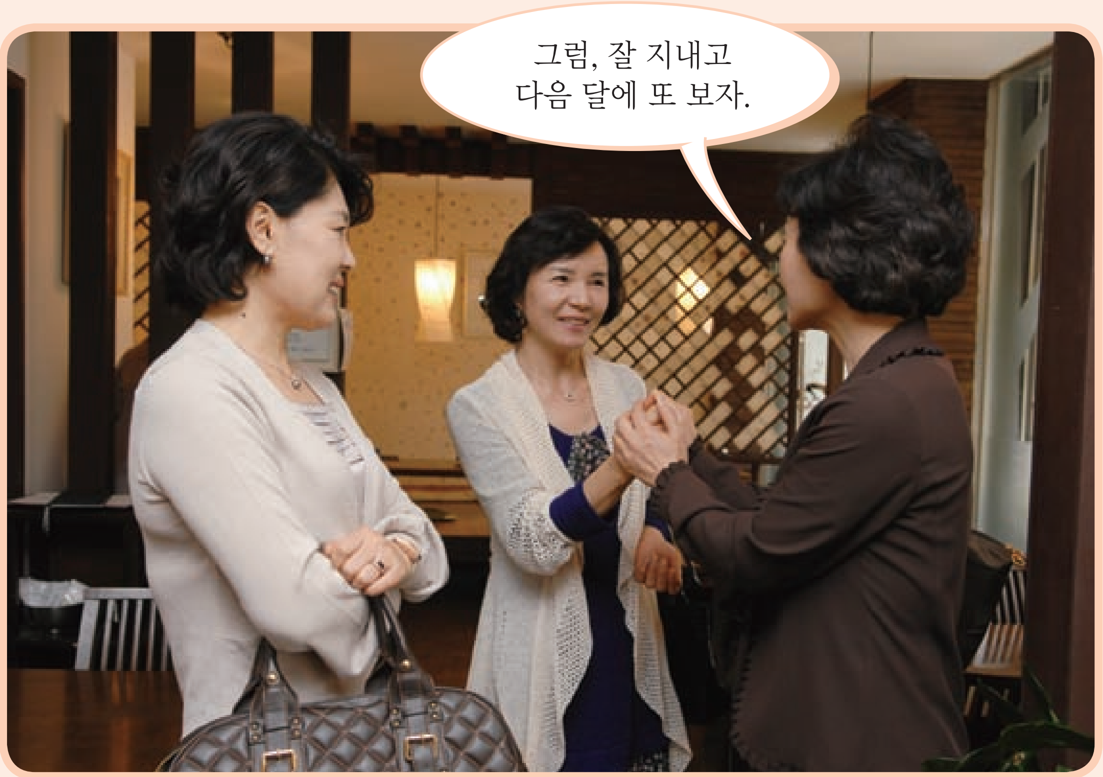

집에 온 지혜 엄마는 고민에 빠진다.

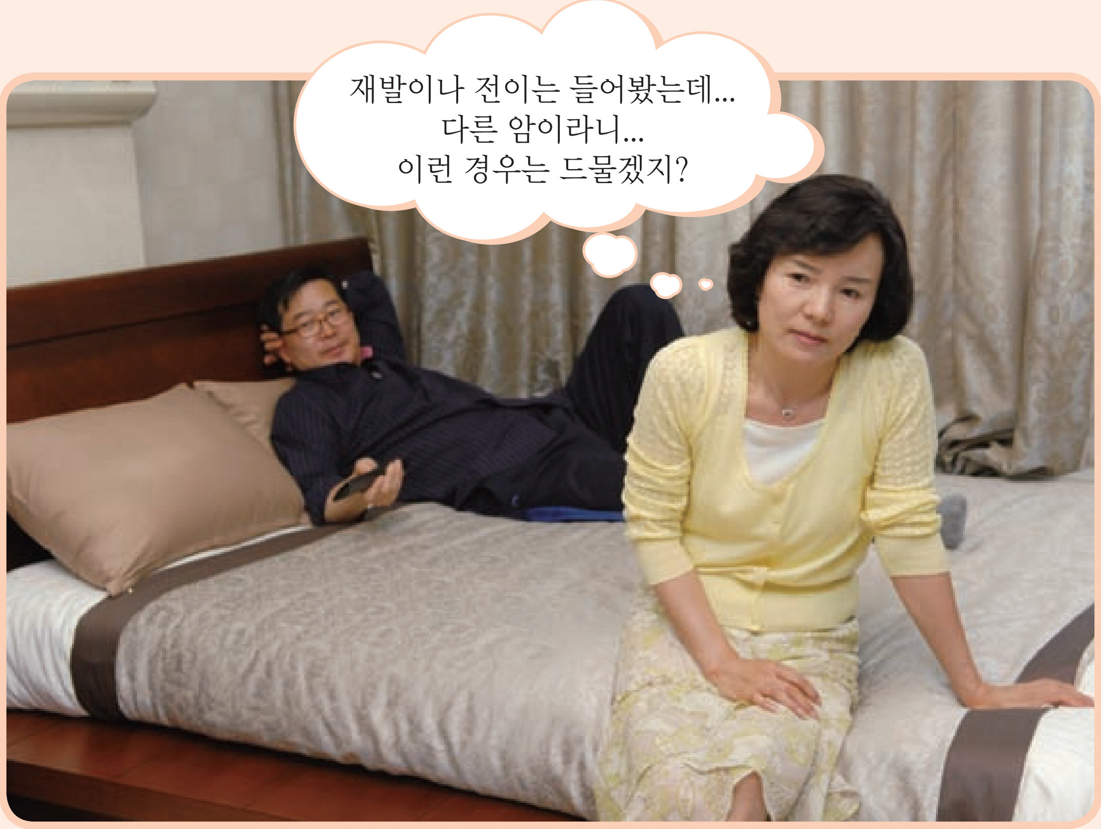

지혜엄마는 남편에게 자신의 고민을 얘기한다.

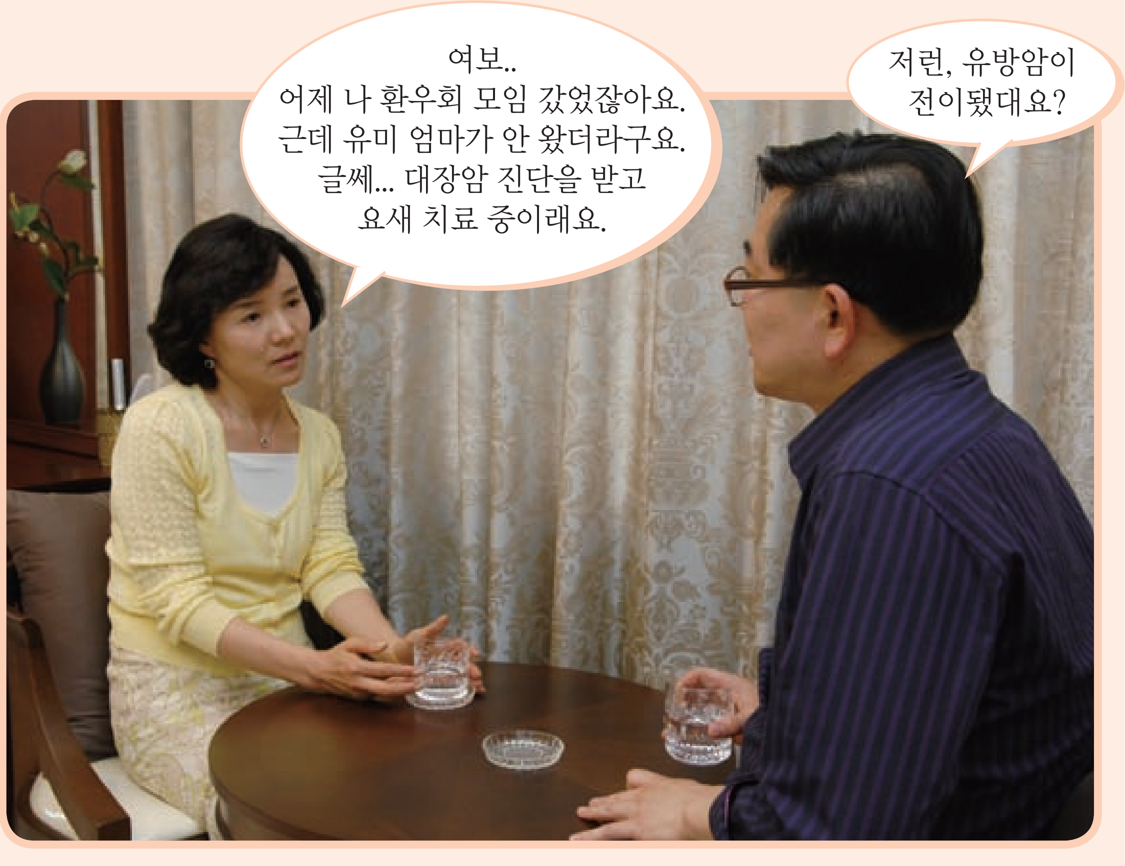

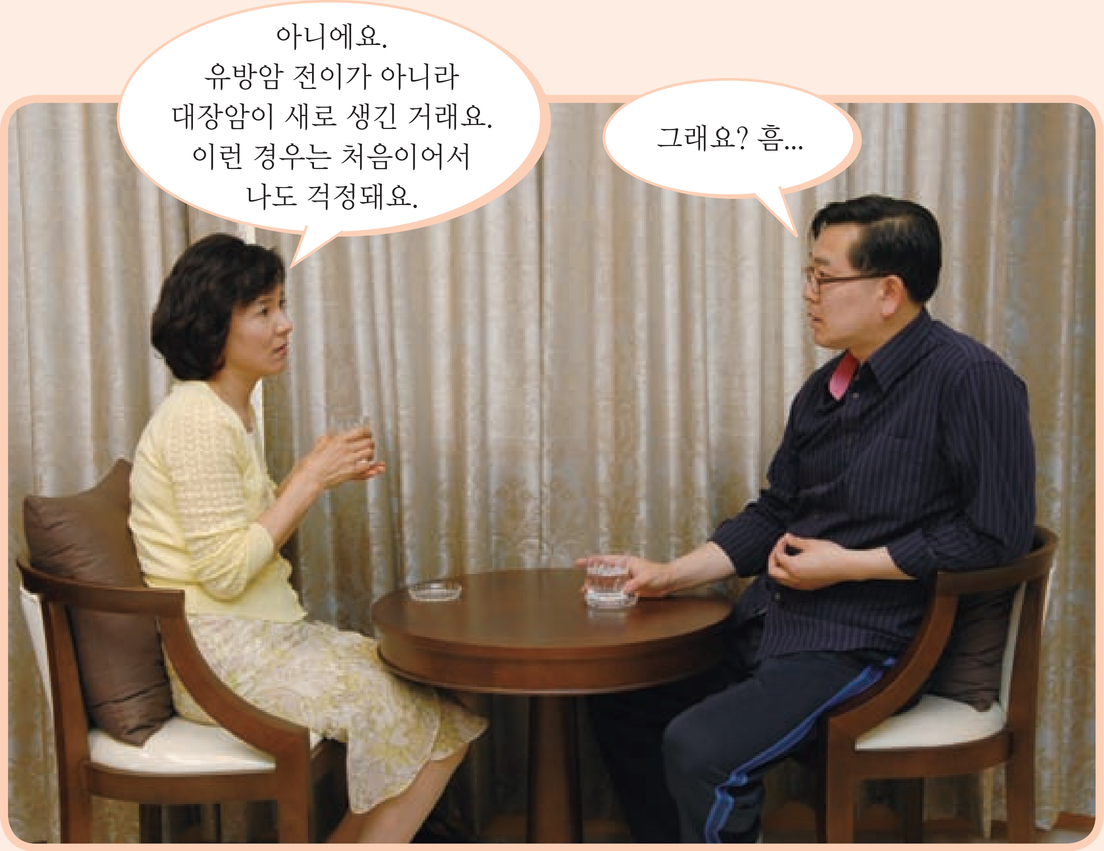
아니에요.
유방암 전이가 아니라
대장암이 새로 생긴 거래요.
이런 경우는 처음이어서
나도 걱정돼요.
그래요? 흠...

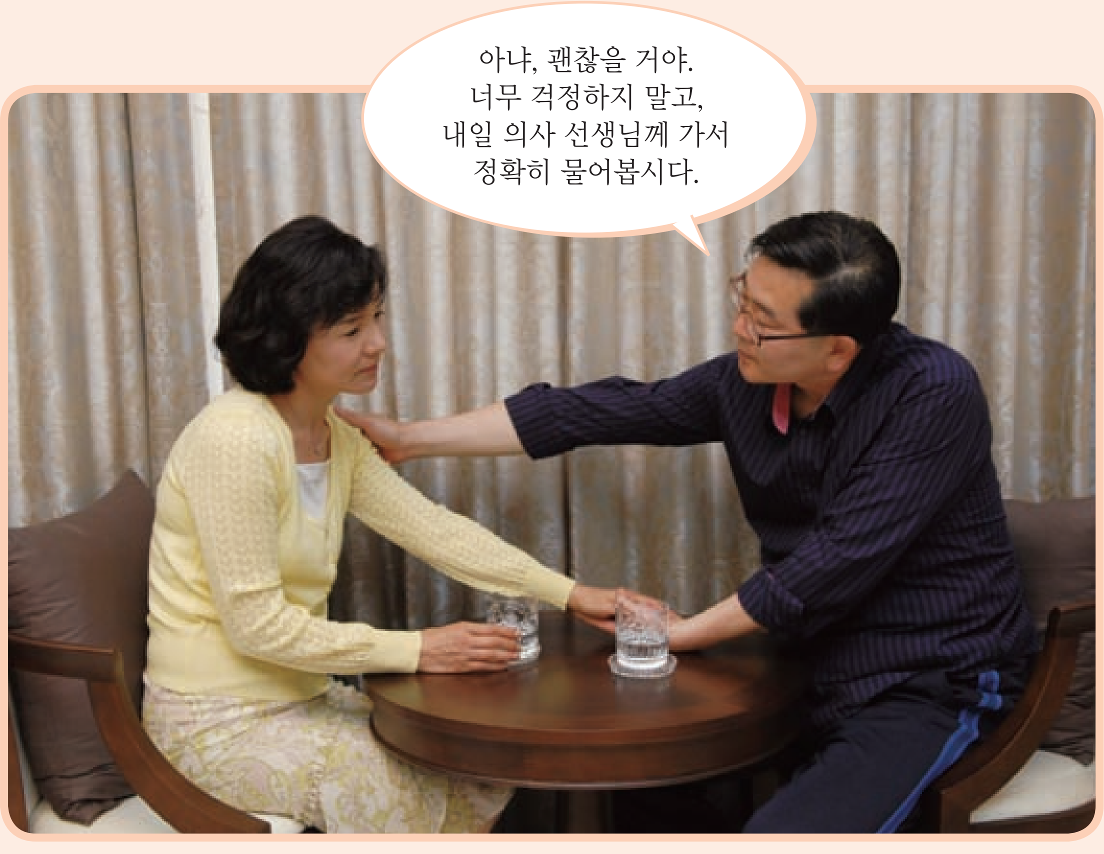
아냐, 괜찮을 거야.
너무 걱정하지 말고,
내일 의사 선생님께 가서
정확히 물어봅시다.

유방암에 대한 정기검진 받으러 간 날...

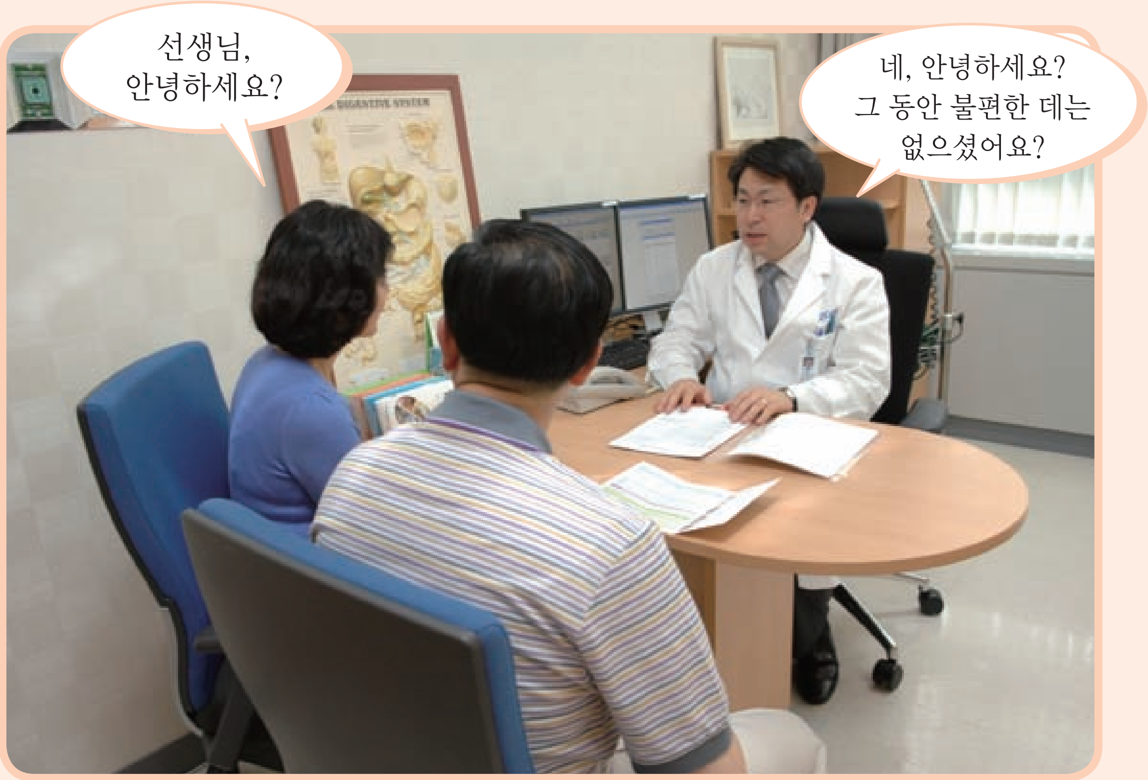

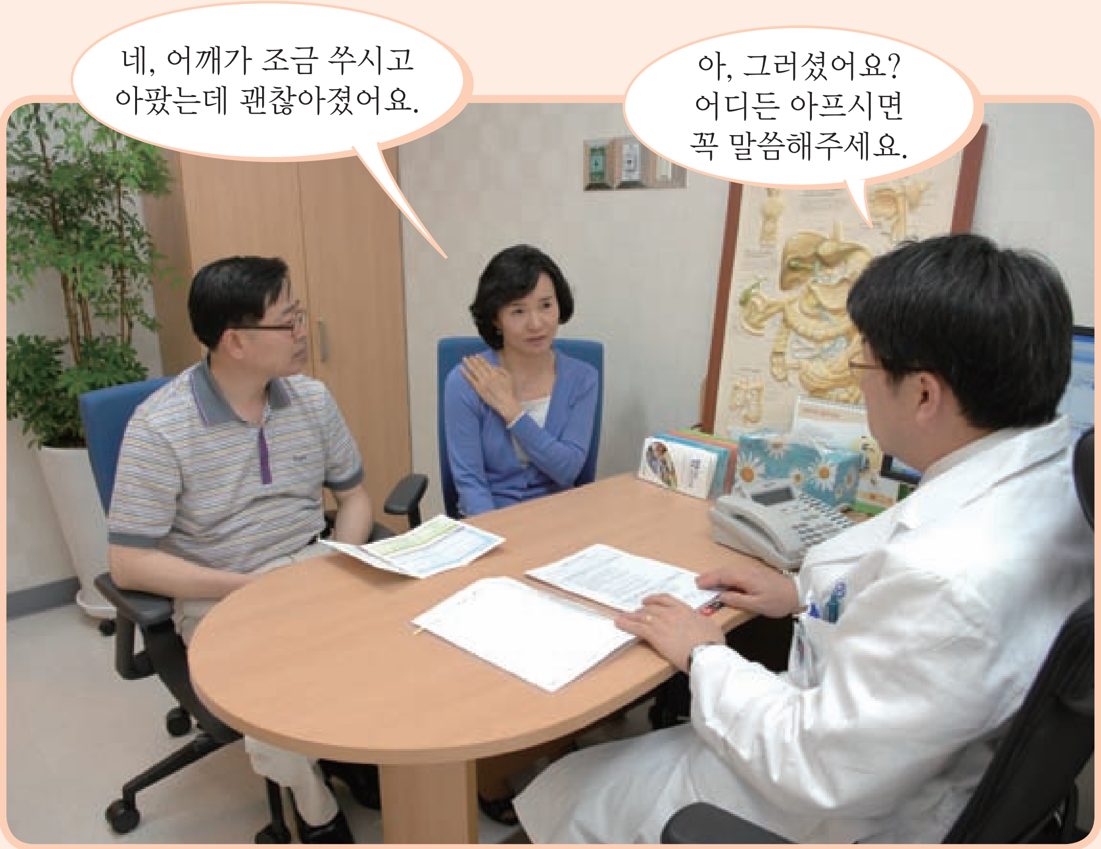

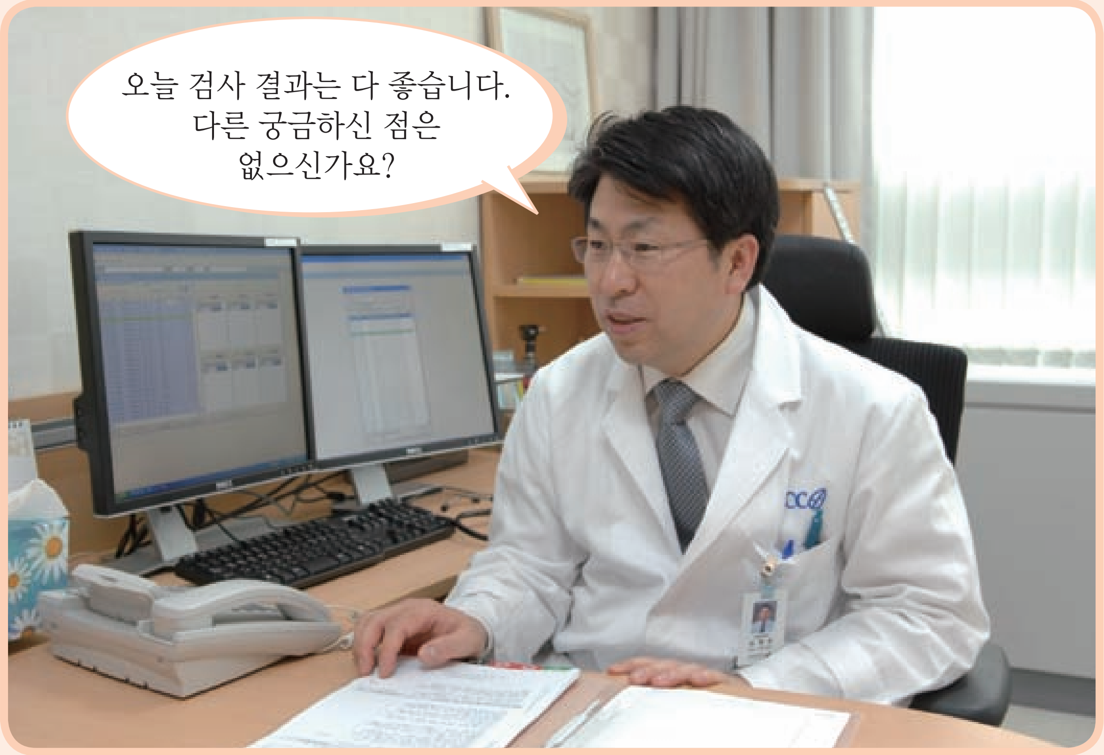
오늘 검사 결과는 다 좋습니다. 다른 궁금하신 점은 없으신가요?

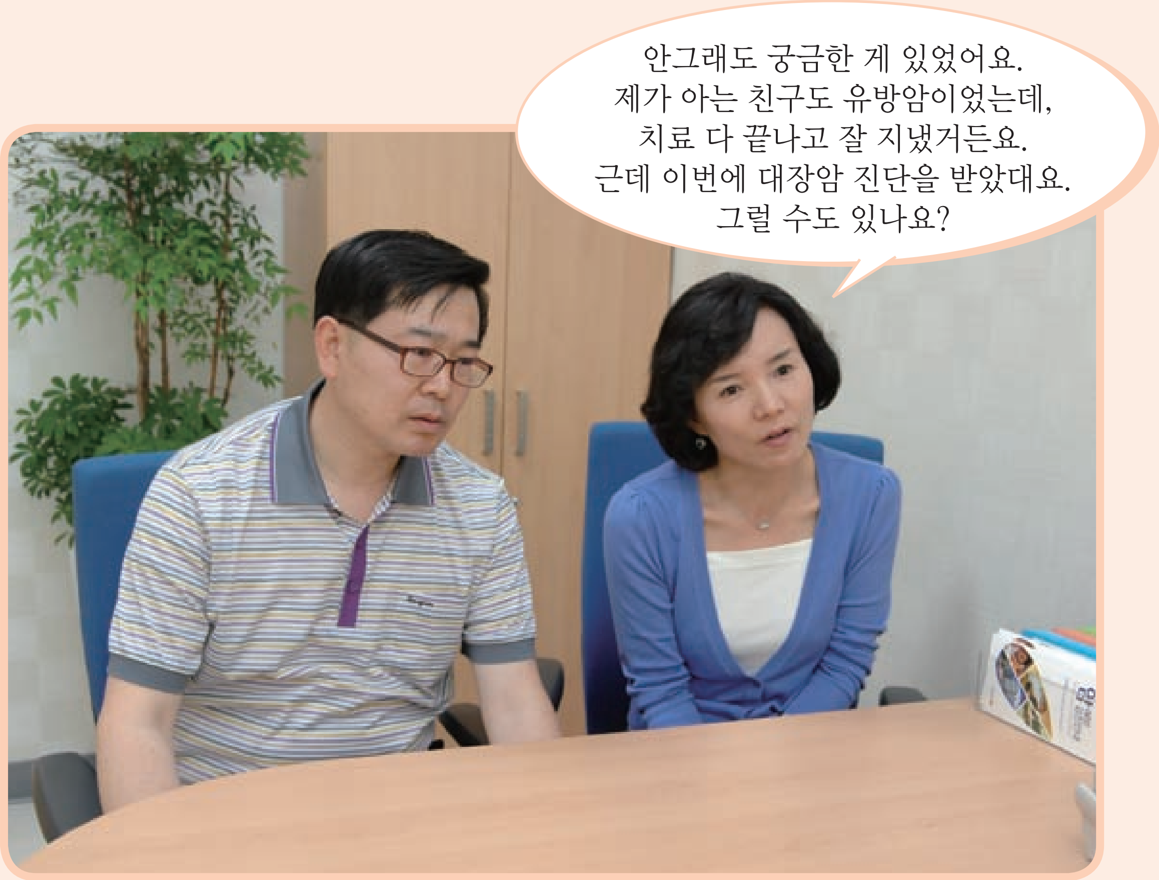
안그래도 궁금한 게 있었어요. 제가 아는 친구도 유방암이었는데, 치료 다 끝나고 잘 지냈거든요. 근데 이번에 대장암 진단을 받았대요. 그럴 수도 있나요?

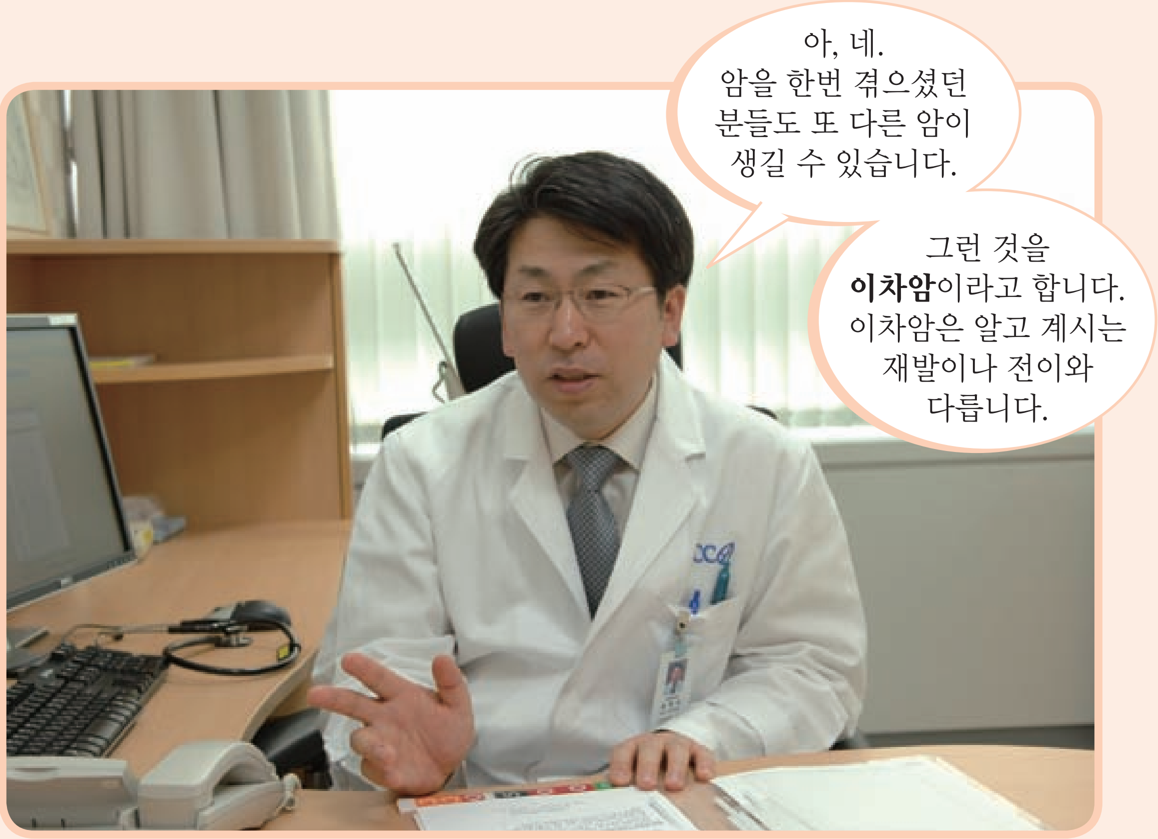
아, 네.
암을 한번 겪으셨던
분들도 또 다른 암이
생길 수 있습니다.
그런 것을
이차암이라고 합니다.
이차암은 알고 계시는
재발이나 전이와
다릅니다.

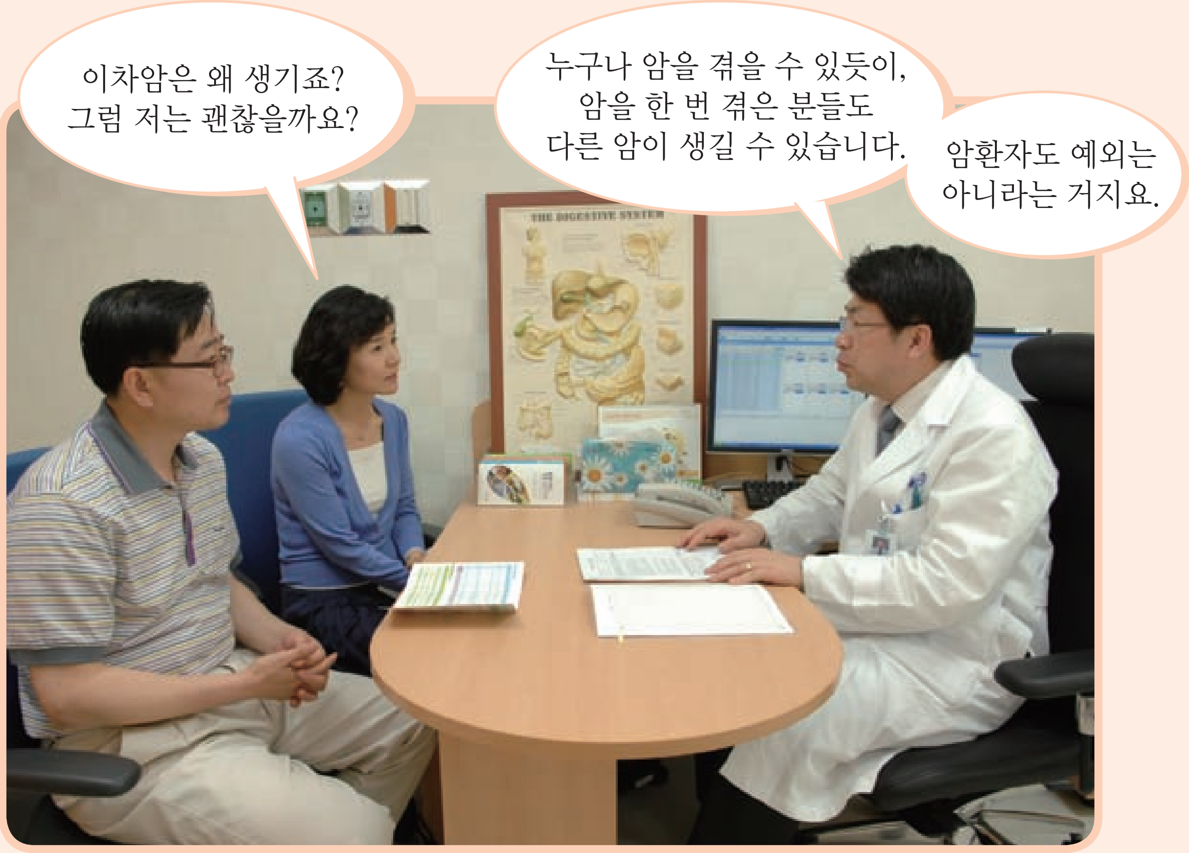
이차암은 왜 생기죠?
그럼 저는 괜찮을까요?
누구나 암을 겪을 수 있듯이,
암을 한 번 겪은 분들도
다른 암이 생길 수 있습니다.
암환자도 예외는
아니라는 거지요.
THE DIGESTIVE SYSTEM

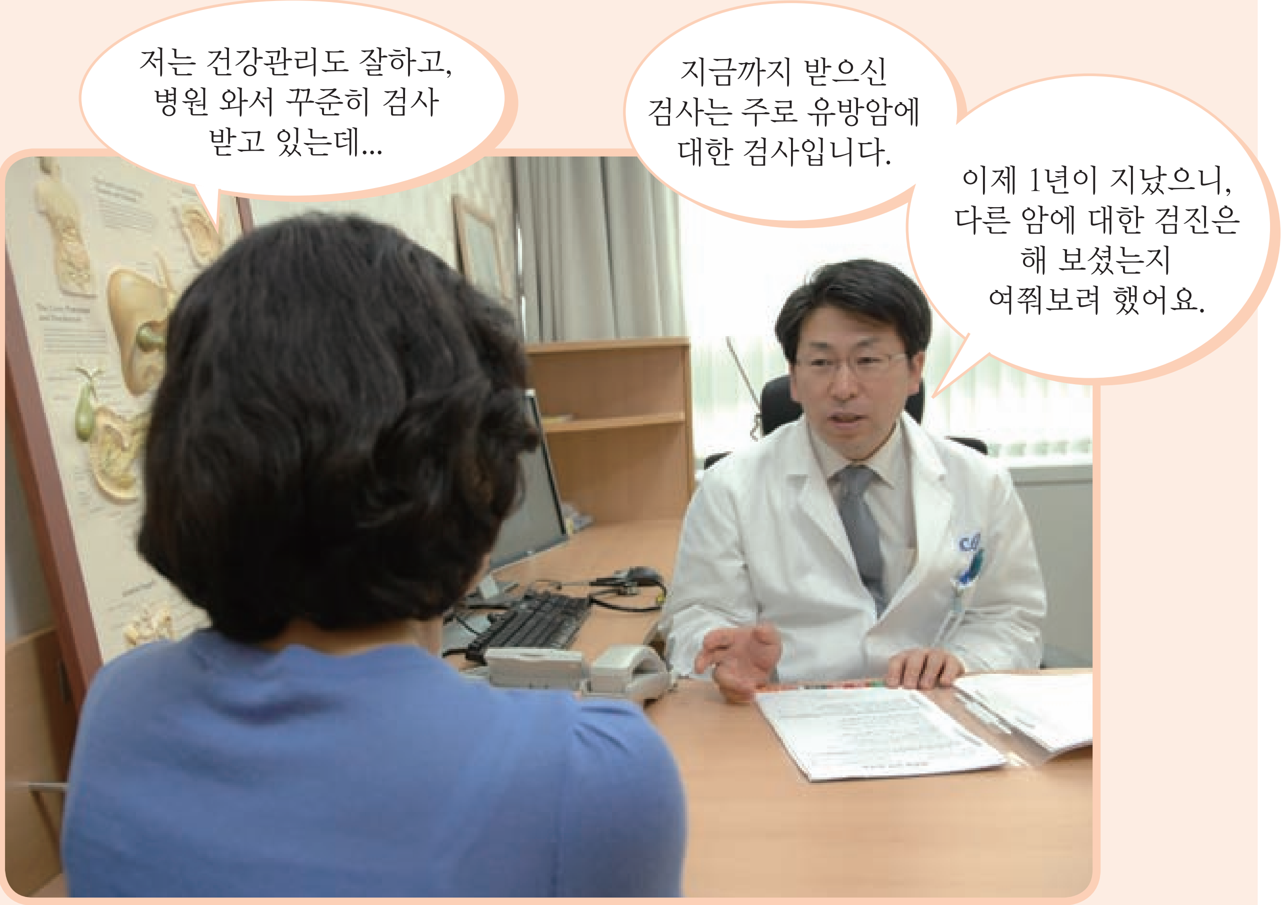

저는 건강관리도 잘하고, 병원 와서 꾸준히 검사 받고 있는데...
지금까지 받으신 검사는 주로 유방암에 대한 검사입니다.
이제 1년이 지났으니, 다른 암에 대한 검진은 해 보셨는지 여쭤보려 했어요.

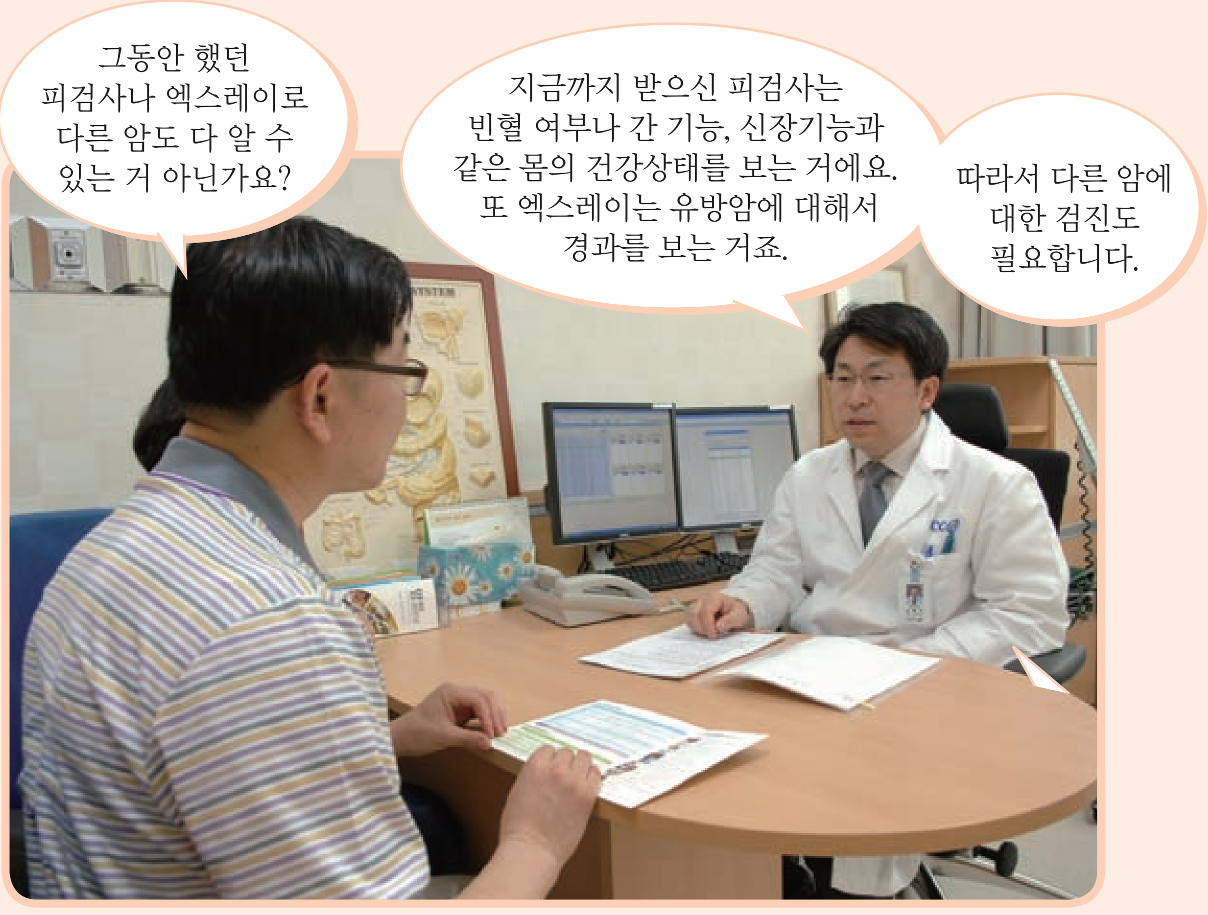

그동안 했던 피검사나 엑스레이로 다른 암도 다 알 수 있는 거 아닌가요?
지금까지 받으신 피검사는 빈혈 여부나 간 기능, 신장기능과 같은 몸의 건강상태를 보는 거에요. 또 엑스레이는 유방암에 대해서 경과를 보는 거죠.
따라서 다른 암에 대한 검진도 필요합니다.

지혜엄마는 암검진에 관한 다양한 교육자료를 받는다.

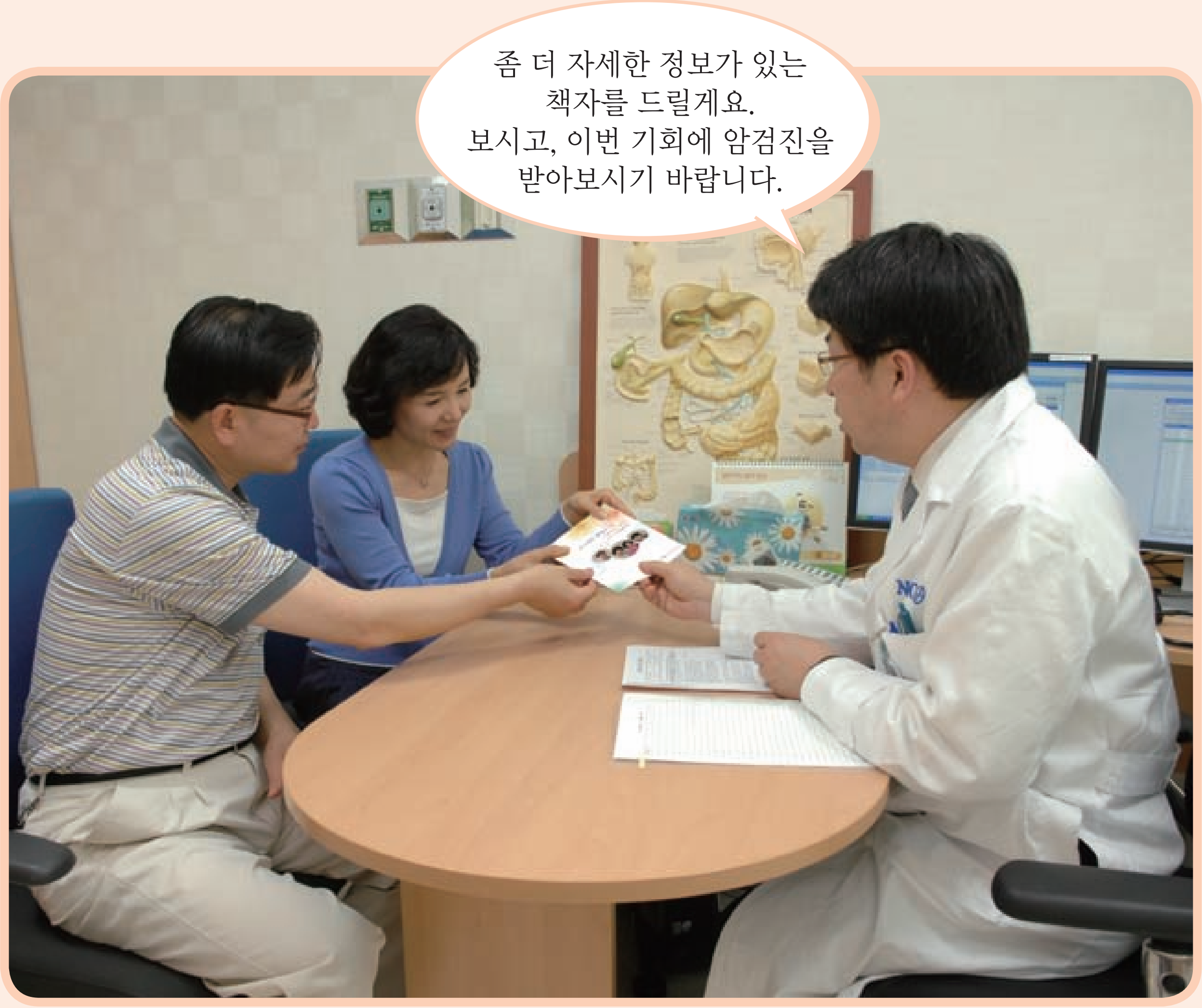

집에 돌아와서 ...

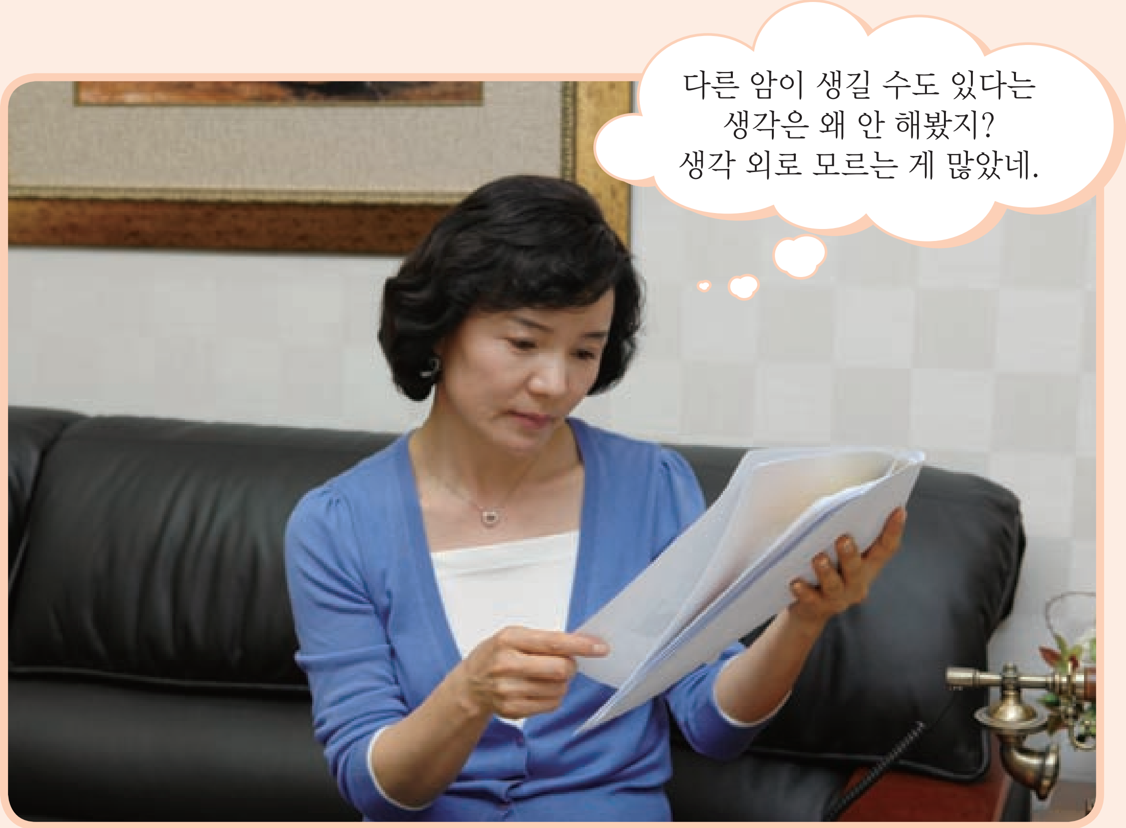
다른 암이 생길 수도 있다는
생각은 왜 안 해봤지?
생각 외로 모르는 게 많았네.

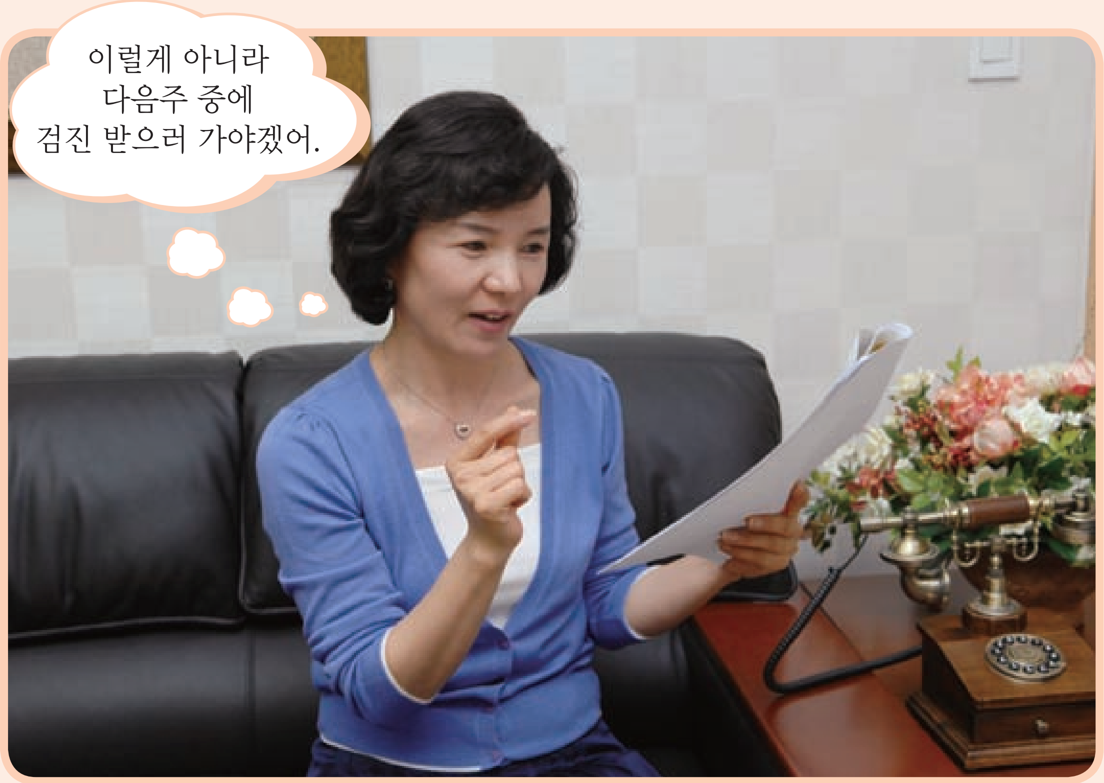
이렇게 아니라
다음주 중에
검진 받으러 가야겠어.

얼마 후, 지혜 엄마는 건강검진센터에서
암검진을 받는다.

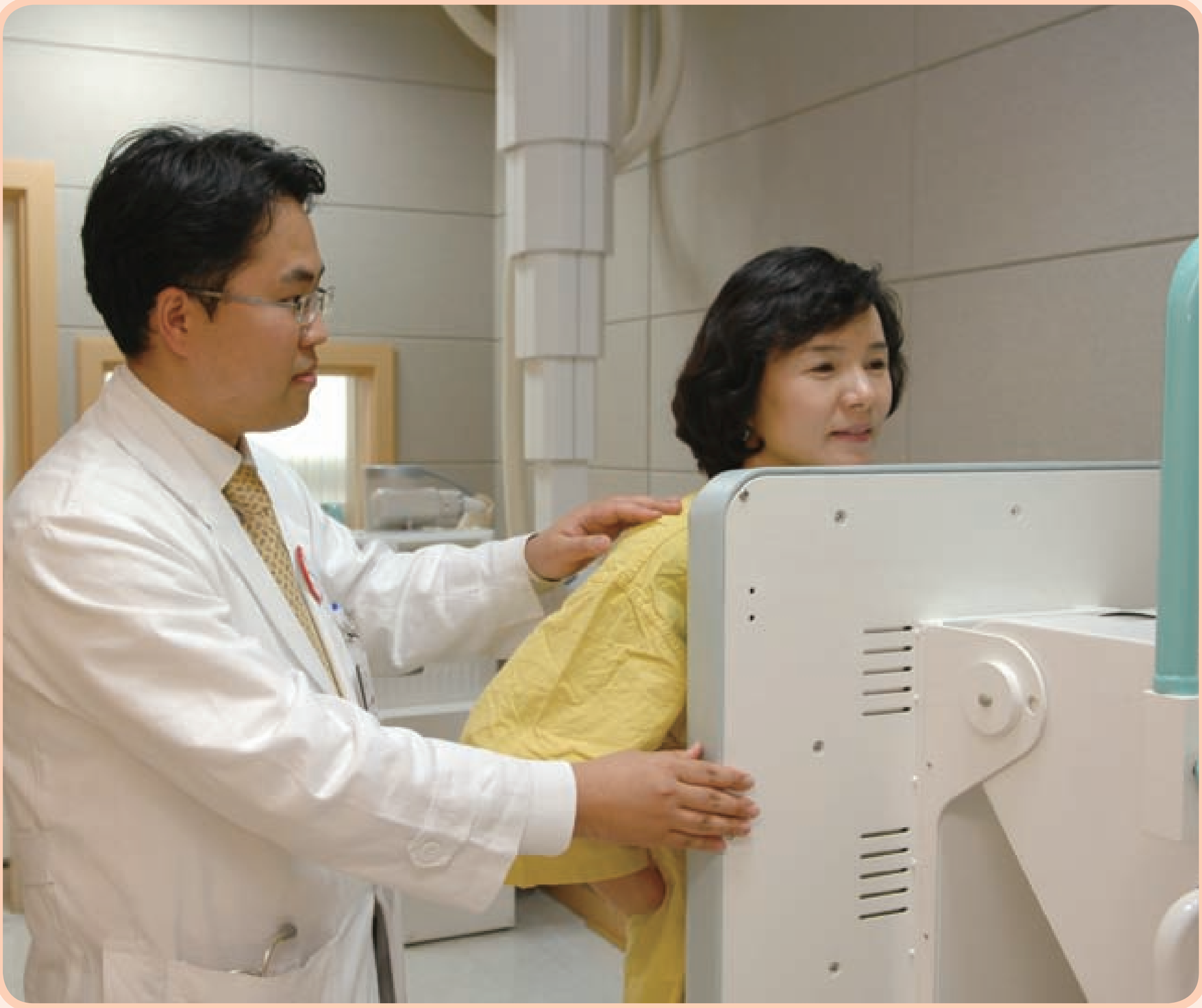

보름 후, 지혜 엄마는 남편과 함께 검진 결과를 들으러 간다.

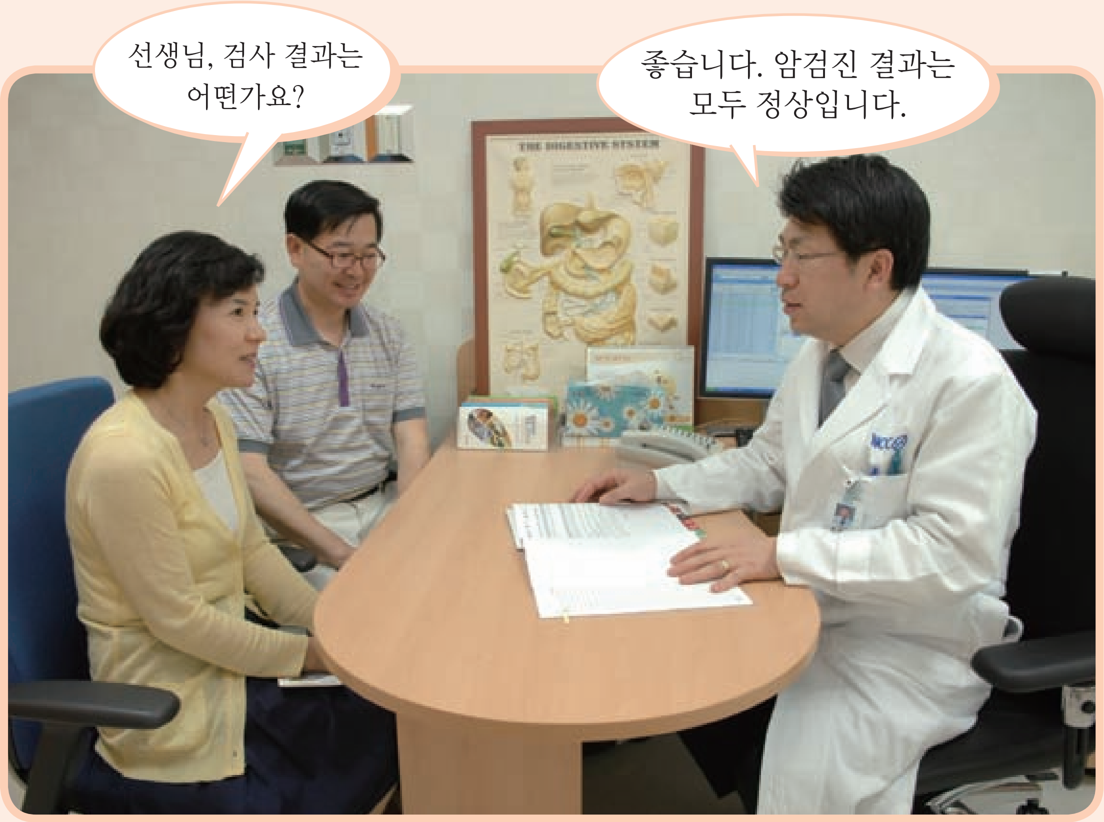

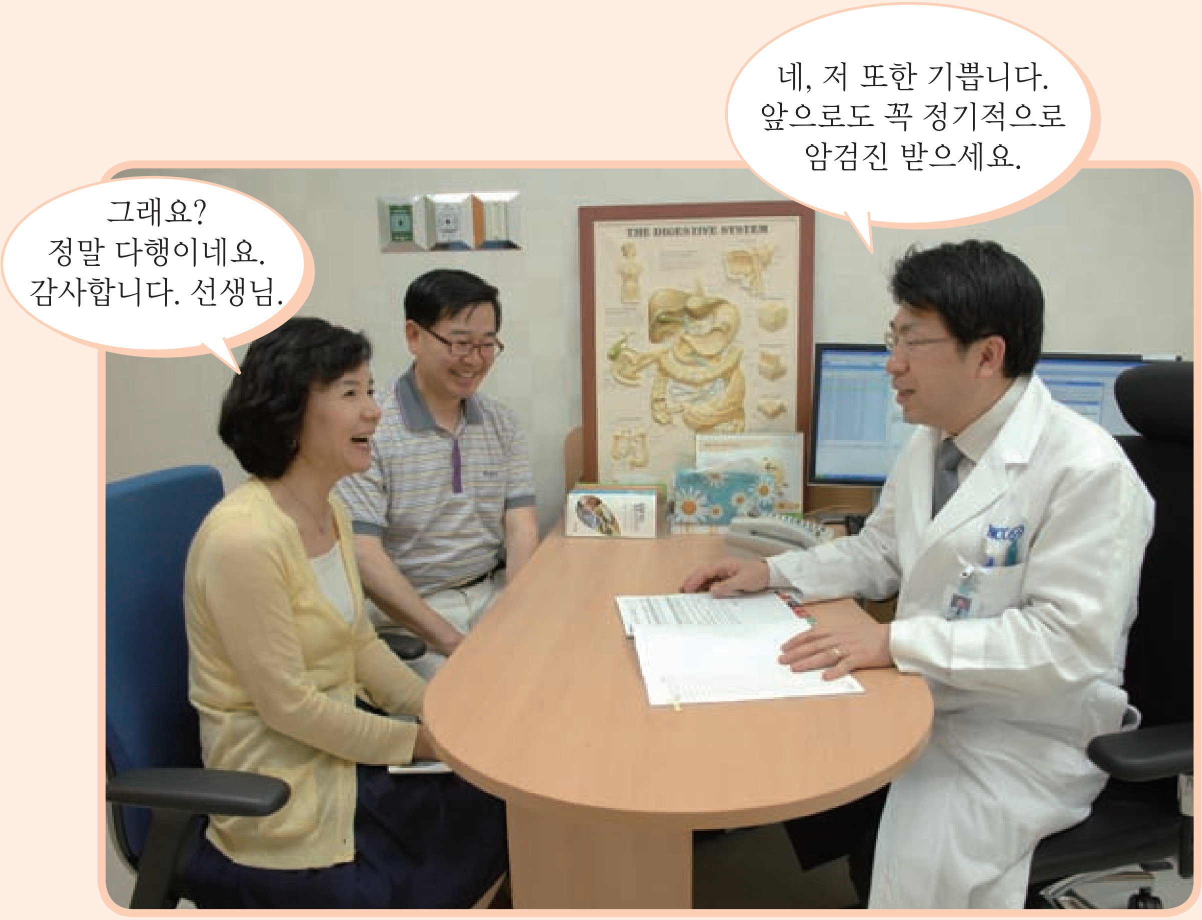

다음 모임에서…

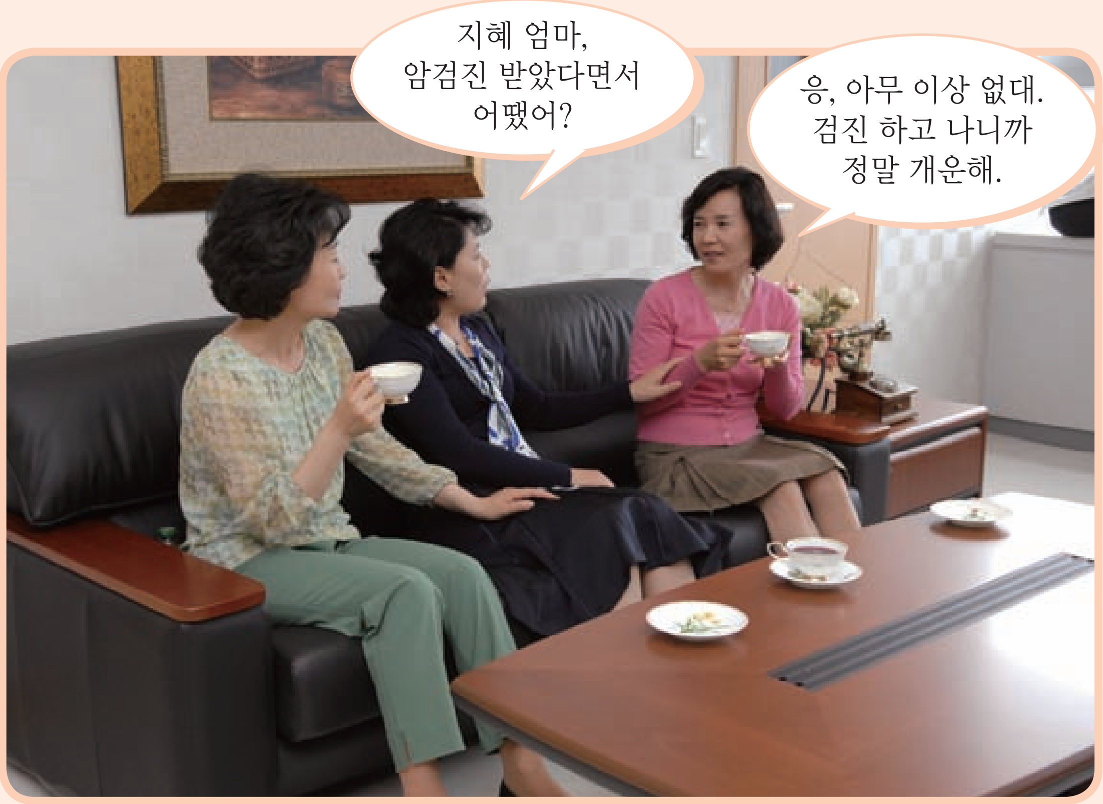
지혜 엄마, 암검진 받았다면서 어땠어?
응, 아무 이상 없대. 검진 하고 나니까 정말 개운해.

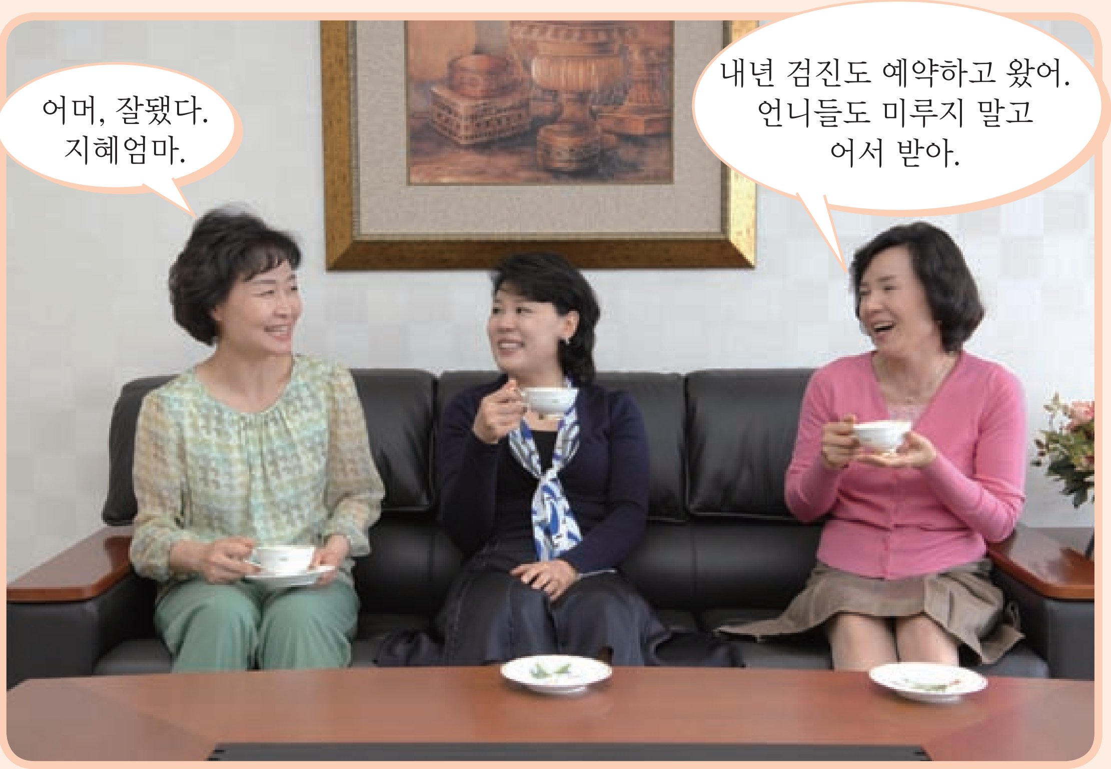
어머, 잘됐다. 지혜엄마.
내년 검진도 예약하고 왔어. 언니들도 미루지 말고 어서 받아.

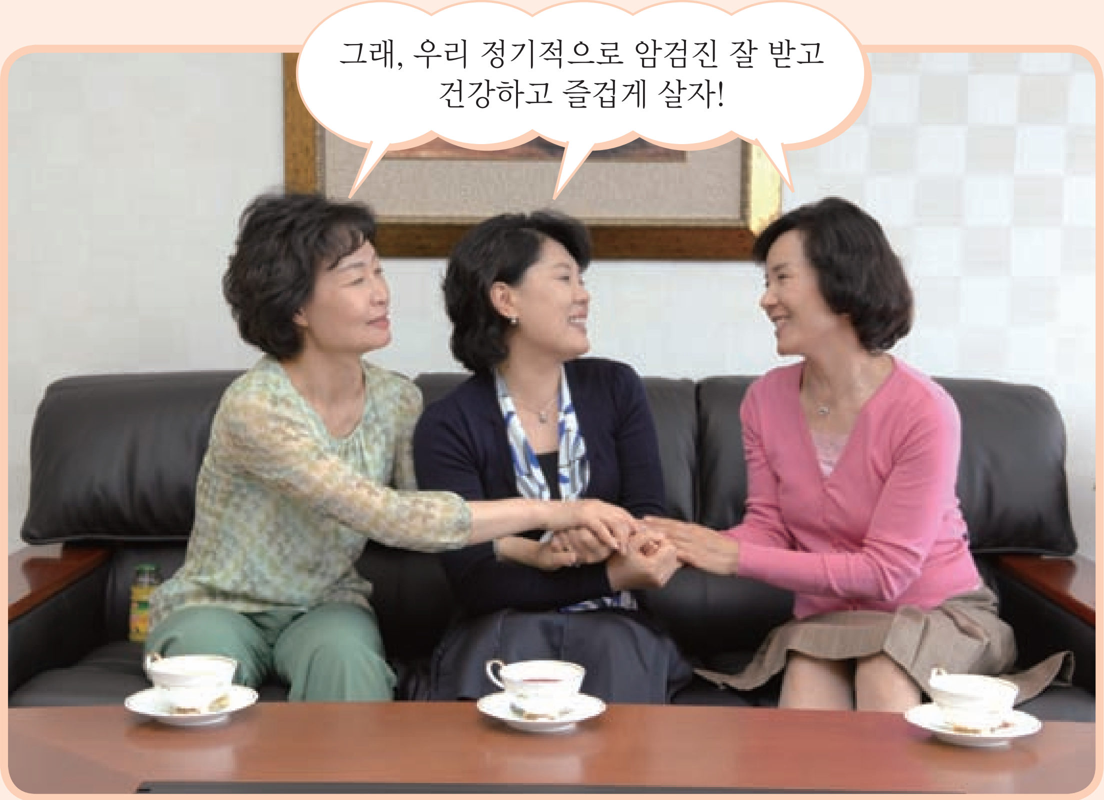
그래, 우리 정기적으로 암검진 잘 받고
건강하고 즐겁게 살자!

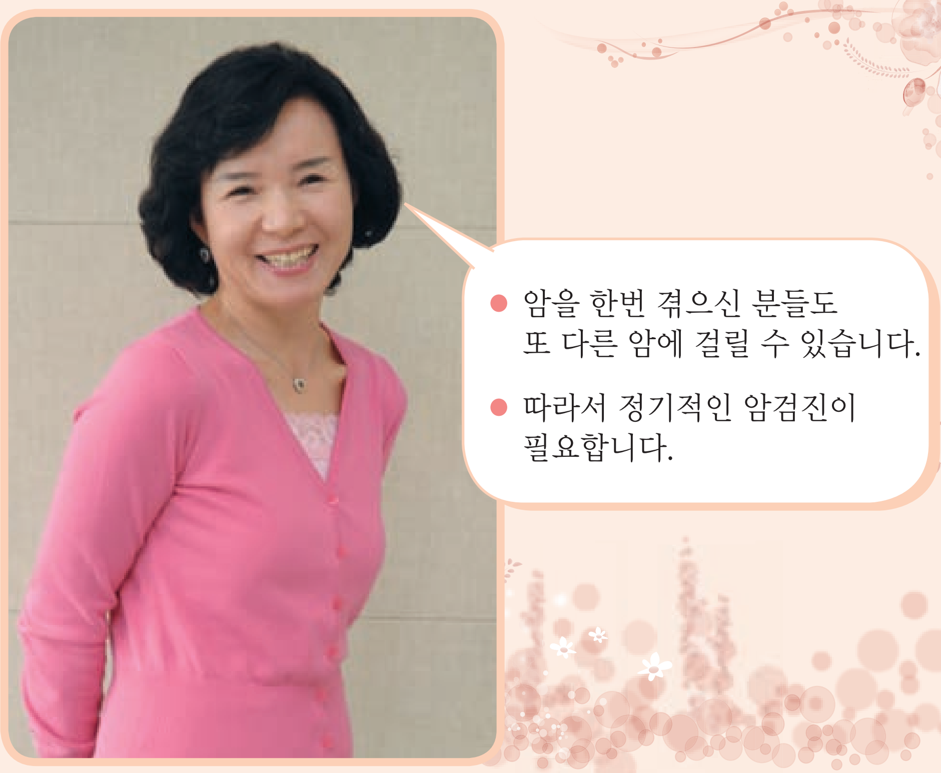
암을 한번 겪으신 분들도
또 다른 암에 걸릴 수 있습니다.
따라서 정기적인 암검진이
필요합니다.

이차암이란 무엇인가요?

한 번 암을 겪으셨던 분들에게서 암 치료 이후에 원래 있었던 암과 무관하게 새롭게 발생하는 암을 이차암이라고 합니다. 이는 원래 있었던 암이 인접 부위에서 다시 자라나는 것을 말하는 재발이나, 다른 부위로 옮겨져서 자라는 전이와는 다른 것입니다.

유방암 환자의 경우 예시

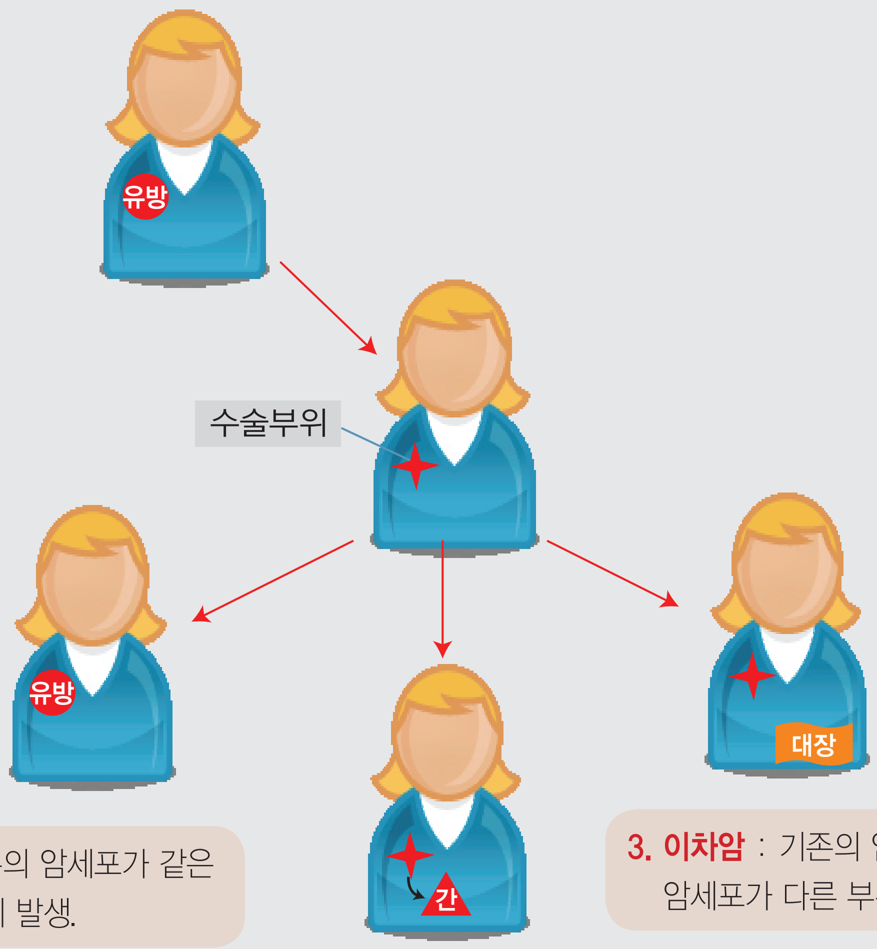

1. 재발 : 기존의 암세포가 같은 부위에 다시 발생.

2. 전이 : 기존의 암세포가 혈관을 타고 다른 부위에 발생.

3. 이차암 : 기존의 암세포와 다른 암세포가 다른 부위에 발생.

이차암은 왜 발생하나요?

누구에게나 암이 발생할 수 있듯이 암환자에게서도 새로운 암이 발생할 수 있습니다. 암 진단 전에 흡연이나 음주를 하거나 비만과 인슐린저항성(당뇨)이 있는 암환자의 경우에 이차암이 더 잘 발생하는 것으로 알려져 있습니다.

이차암의 위험도는 어떻게 되나요?

한 번 암을 겪으셨던 분들은 같은 나이와 성별의 일반인에 비하여 새로운 암이 발생할 위험도가 약간 더 높은 것으로 알려져 있습니다.

이차암은 어떻게 조기에 발견할 수 있나요?

- 암검진은 일반적으로 신체적 이상이나 증상이 없고 스스로 건강하다고 생각될 때 검사를 받음으로써 병을 조기에 발견하여 치료하고자 하는 것입니다.
- 암을 한 번 겪었던 분들도 이차암이 발병하였을 때 검진을 통해 암을 조기에 발견하여 치료하면 완치 가능성을 크게 높일 수 있습니다.
- 암 환자들도 최소한 일반인에서 적용되는 암조기검진 권고안대로 검진을 받는 것이 권장됩니다.
- 암 조기 검진을 받으려면 병원의 종합 검진 프로그램을 이용하거나, 국가 암검진 프로그램을 이용할 수 있습니다.

이차암 예방은 어떻게 하나요?

암이 완치되신 분들도 반드시 금연하도록 해야 합니다. 또한 비만도가 심할수록, 혈당이 높을수록 암 재발율도 높아지고 이차암 발생율도 높아집니다. 암 진단 후 신체활동량이 감소하면서 체중 증가는 매우 흔하게 나타납니다. 따라서 운동과 식이 조절을 통해 추가적인 체중 증가는 피해야 합니다.

암완치자의 비만 및 신체활동 관리 방법

1) 추가적인 체중 증가 피하기.
2) 신체활동량을 늘리고 거의 매일 30분 이상 중등도의 신체활동 하기.
3) 야채와 과일 섭취를 늘리고 지방섭취를 줄이는 건강한 식이습관을 유지하기.

※ 자세한 사항은 주치의와 상담하세요.

자기 확인 테스트

01	암으로 진단 받은 이후에 위암에 대한 검진으로 위장 조영 촬영이나 위내시경 검사를 2년 이내에 받으신 적이 있습니까?	☐ 예<br>☐ 아니오
02	암으로 진단 받은 이후에 대장암에 대한 검진으로 분별잠혈검사 또는 대장내시경 검사나 대장 이중 조영검사를 2년 이내에 받아보신 적이 있습니까?	☐ 예<br>☐ 아니오
03	암으로 진단 받은 이후에 유방암에 대한 검진으로 유방촬영을 2년 이내에 받으신 적이 있습니까?	☐ 예<br>☐ 아니오
04	암으로 진단 받은 이후에 자궁경부암에 대한 검진으로 자궁경부세포 검사를 2년 이내에 받으신 적이 있습니까?	☐ 예<br>☐ 아니오

※ 위의 항목 중 하나라도 아니오를 선택하신 분은, 암검진에 대해 주치의와 상의하십시오.

암환자에게 도움이 되는 기관 정보

국가암정보센터	www.cancer.go.kr	☎ 1577-8899
보건복지콜센터	www.129.go.kr	☎ 129
국민건강보험공단	www.nhic.or.kr	☎ 1577-1000

자료 개발 자문 및 검토

김영우 (국립암센터 암예방검진센터 · 위암센터)
오재환 (국립암센터 대장암센터)
정기욱 (국립암센터 유방암센터)
김석원 (국립암센터 유방암센터)
장윤정 (국립암센터 암정보교육과)
김나연 (삼성서울병원 암교육센터)
이정언 (삼성서울병원 유방암센터)
김진용 (삼성서울병원 대장암센터)
김수연 (삼성서울병원 암교육센터)

자료 개발에 도움을 주신 분들

등장인물	– 지혜 어머니 : 임사라
	– 지혜 아버지 : 서정민
	– 환우 1 : 하수연
	– 환우 2 : 이재순
	– 의사1 : 김영우 (국립암센터 암예방검진센터 · 위암센터)
	– 의사2 : 신동욱 (국립암센터 국가암관리사업단 · 가정의학클리닉)
촬영	김정민 (국립암센터 암정보교육과)
촬영 협조	국립암센터 암예방검진센터
	한식당 여자만
연구비지원	국립암센터 기관고유 연구사업
개발기관	국립암센터
개발자	신동욱 (국립암센터 국가암관리사업단 · 가정의학클리닉)
	조주희 (삼성서울병원 암교육센터)
	백영지 (국립암센터 국가암관리사업단)
개발연도	2009년 6월

이 교육자료는 국립암센터 기관고유연구사업 (grant 0710422-3)연구 목적으로 개발되었습니다.

이차암 조기 발견을 위해,
정기적인 암검진을 받으세요!

국가 암검진 프로그램

대상암	검진대상	검진방법	검진주기
위암	40세 이상 남녀	위장조영촬영술 또는 위내시경검사	2년
간암	40세 이상 남녀로 간경변증이나 B형 간염바이러스 항원 또는 C형 간염바이러스 항체 양성으로 확인된 자	간초음파검사 + 혈청알파태아단백검사	6개월
대장암	50세 이상 남녀	분변잠혈반응검사 결과 이상 소견시 대장내시경 검사 또는 이중조영바륨검사	1년
유방암	30세 이상 여성	유방자가검진	매월
	40세 이상 여성	유방촬영술 + 유방 임상진찰 권장	2년
자궁경부암	30세 이상 여성	자궁경부세포검사	2년

암을 한 번 겪으신 분들을 위한 안내서

더욱 소중한 내 몸을 위한 아름다운 실천

▶ 건강보조식품에 대한 바른 이해 ◀

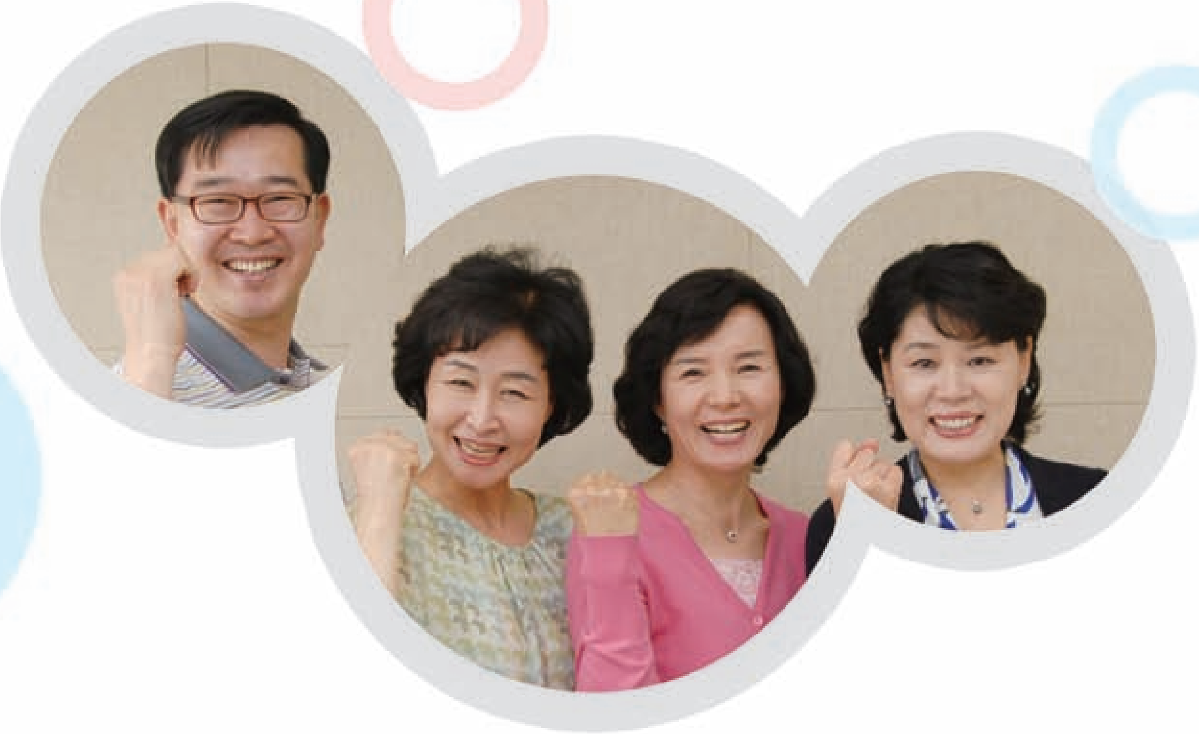

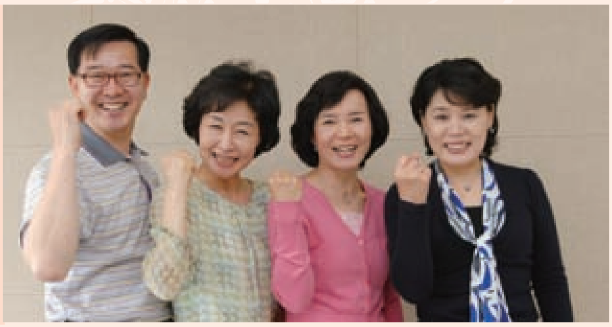

배경 설명

목 적 본 교육자료의 목적은 건강보조식품에 대한 암환자들의 이해를 도와 올바른 건강관리를 도모하기 위함입니다.

줄 거 리 암 치료후 건강관리에 부쩍 관심이 높아진 민호 엄마는 혜미 엄마와 통화 중, 암환자들이 많이 사용하고 있다는 건강보조식품인 '다좋아 천연진액'에 관한 이야기를 듣고 사용을 고민하게 된다.

등장인물

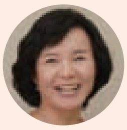

민호엄마, 유방암 환자

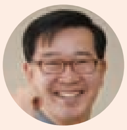

민호 아빠

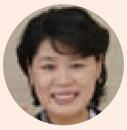

혜미엄마, 대장암 환자, 민호엄마의 고등학교 동창

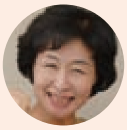

영희엄마, 남편이 위암 환자, 민호엄마의 고등학교 동창

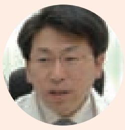

민호엄마의 주치의

민호 엄마는 혜미엄마로부터 건강보조식품인 '다좋아 천연진액'에 대한 이야기를 듣는다.

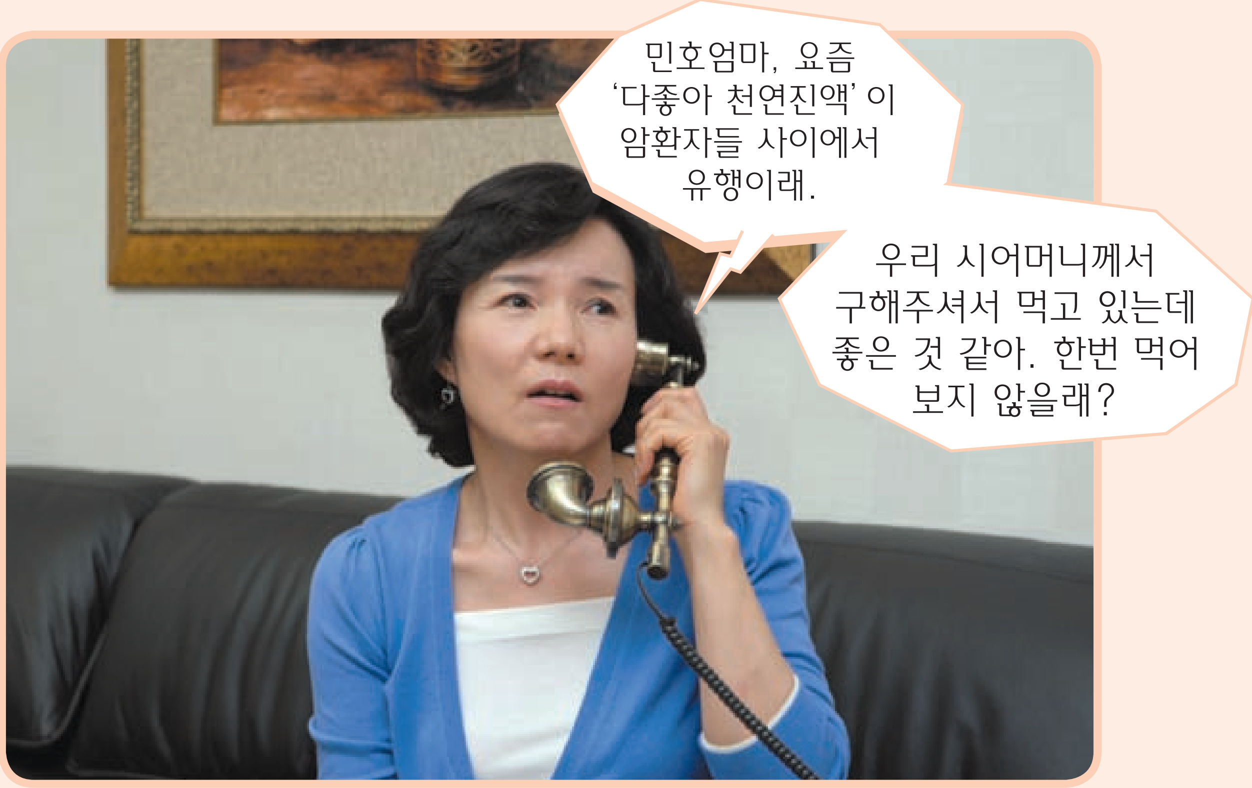

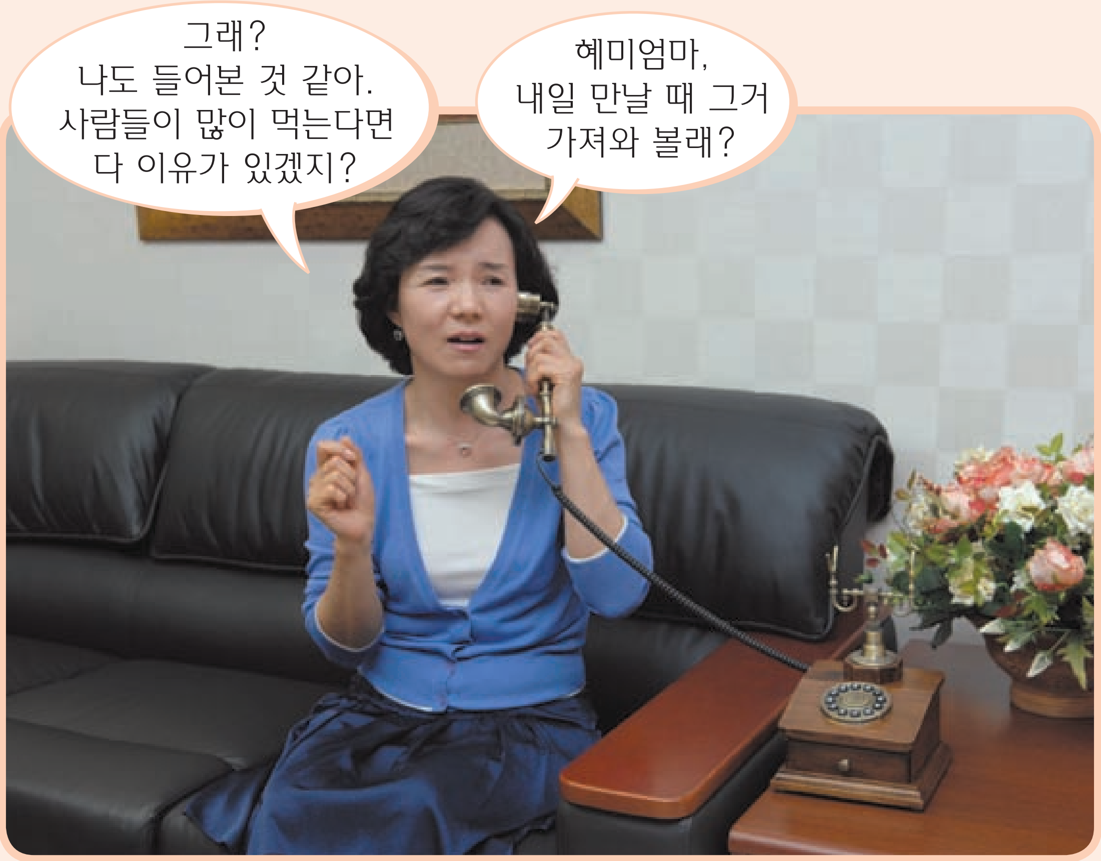

다음 날, 민호엄마와 혜미엄마, 영희엄마가 찻집에서 만나
'다좋아 천연진액' 에 대해 이야기 한다.

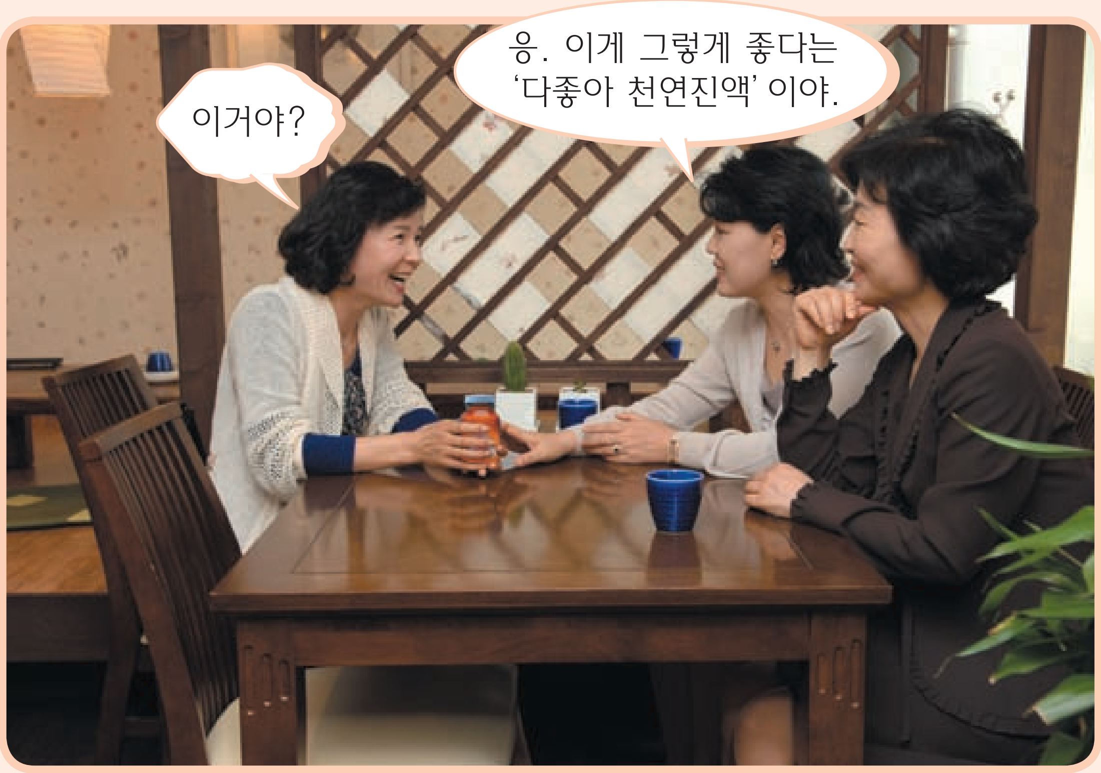

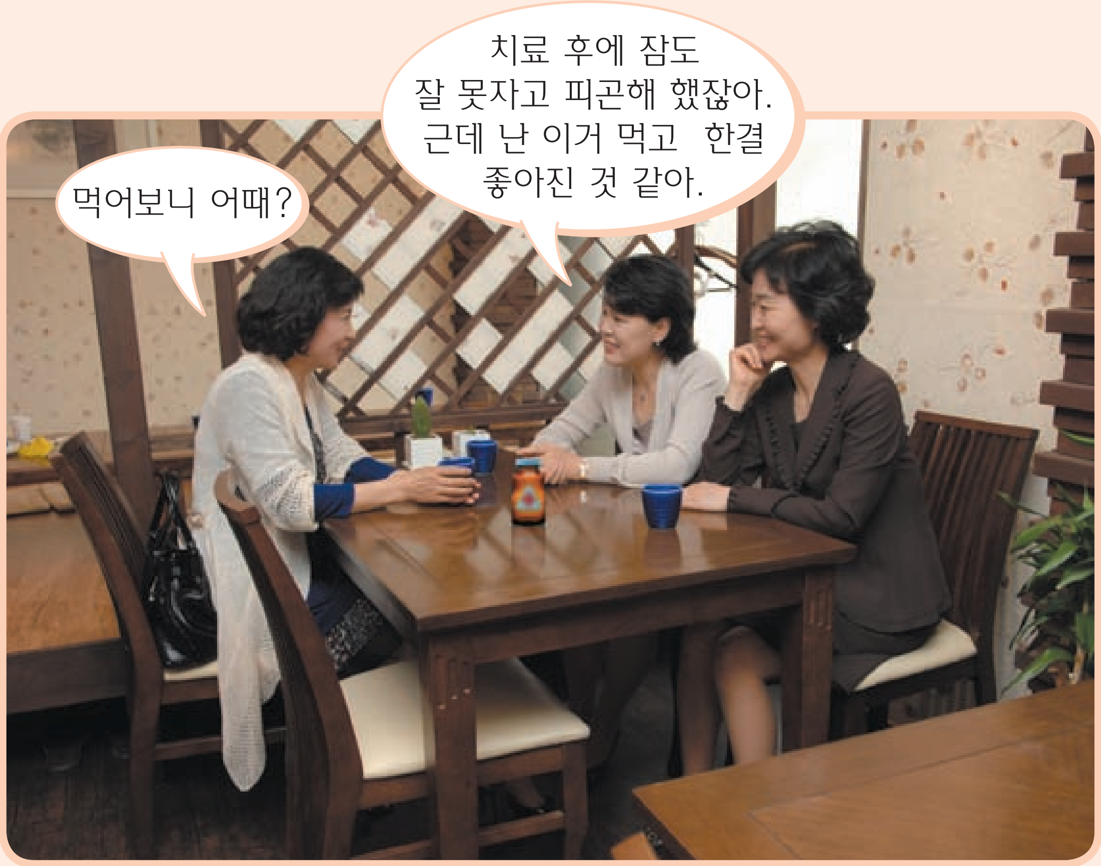

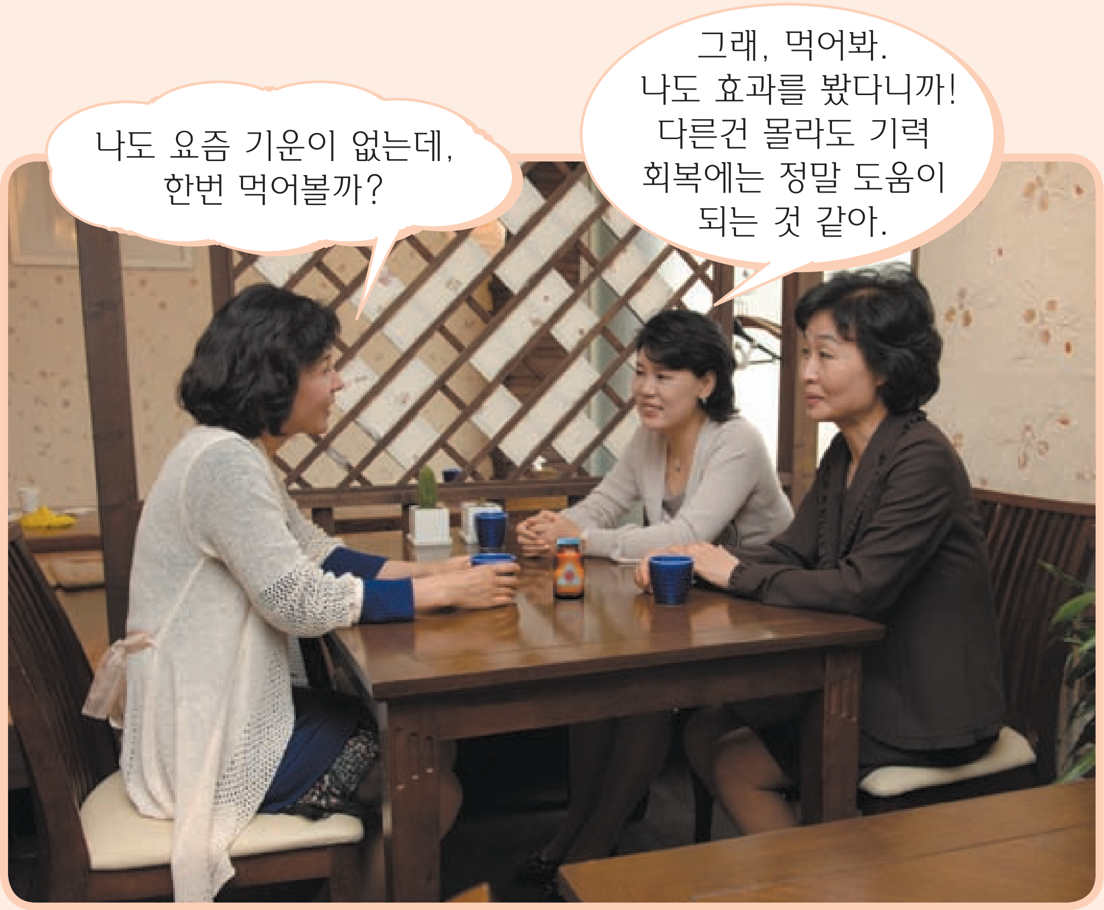
나도 요즘 기운이 없는데,
한번 먹어볼까?
그래, 먹어봐.
나도 효과를 봤다니까!
다른건 몰라도 기력
회복에는 정말 도움이
되는 것 같아.

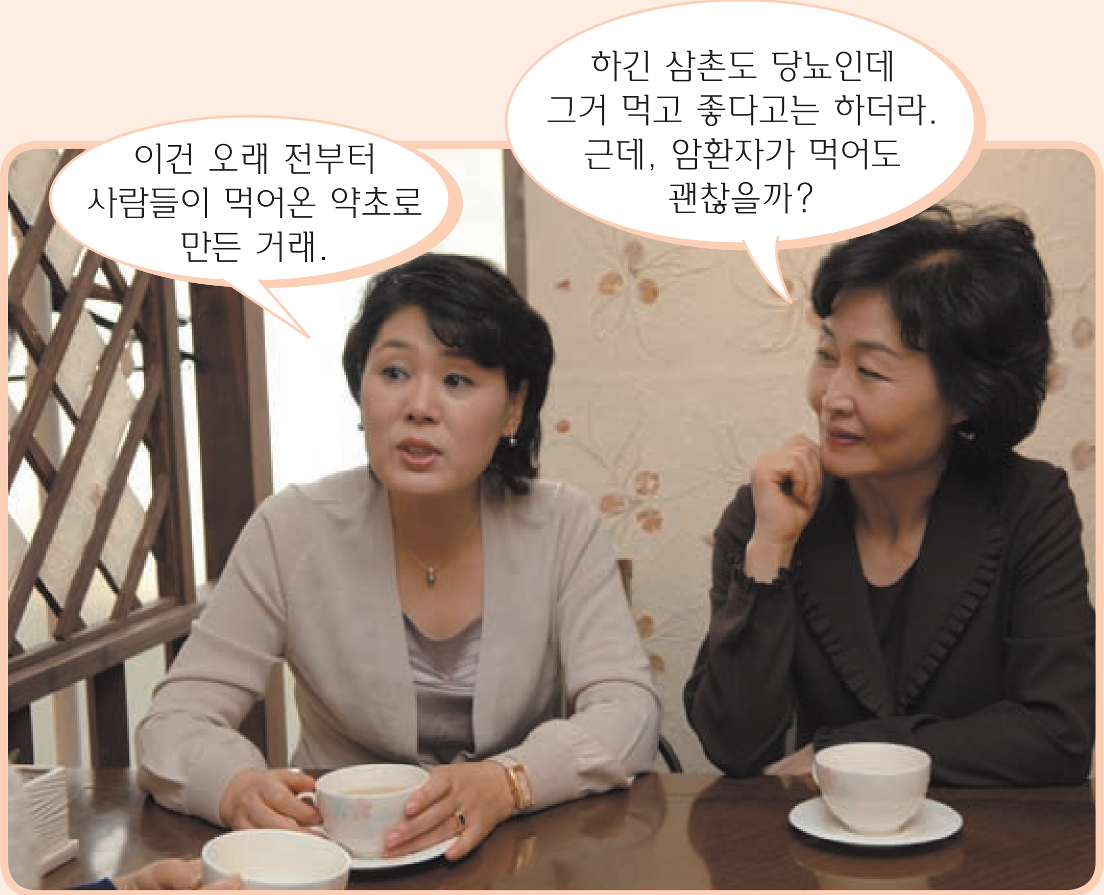
이건 오래 전부터
사람들이 먹어온 약초로
만든 거래.
하긴 삼촌도 당뇨인데
그거 먹고 좋다고는 하더라.
근데, 암환자가 먹어도
괜찮을까?

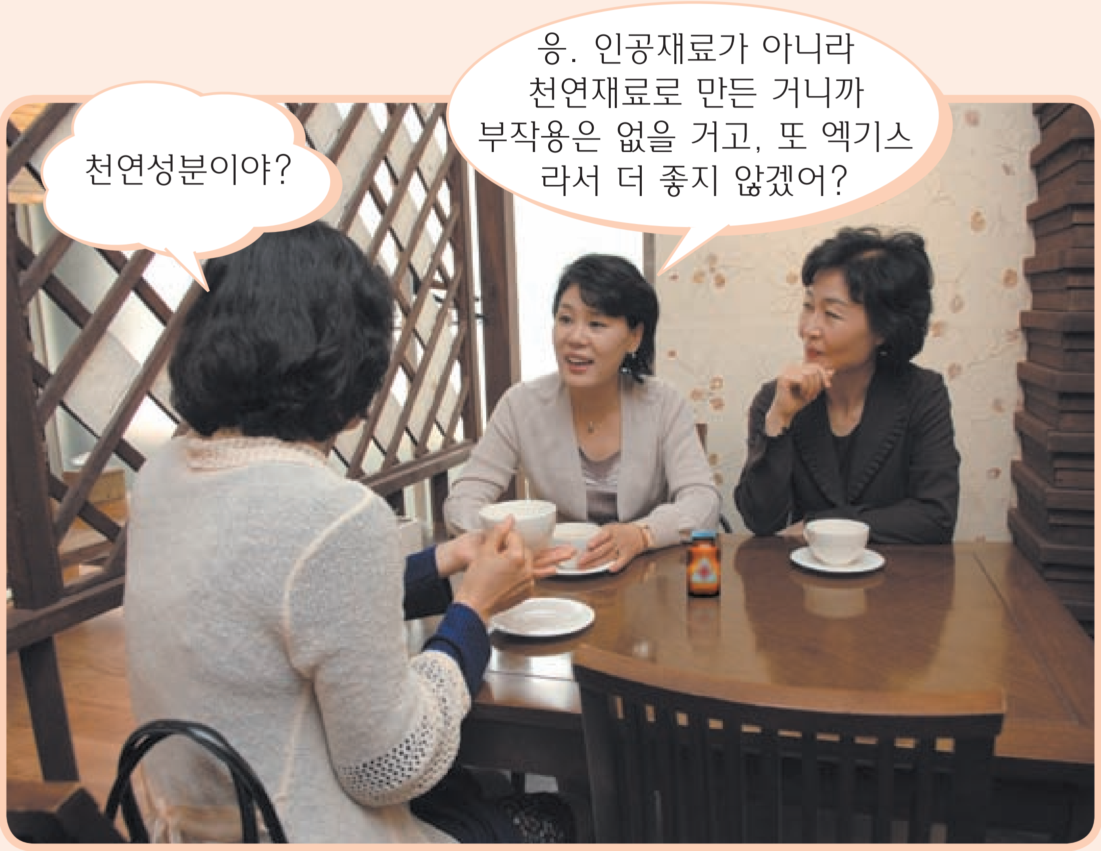
천연성분이야?
응. 인공재료가 아니라 천연재료로 만든 거니까 부작용은 없을 거고, 또 엑기스라서 더 좋지 않겠어?

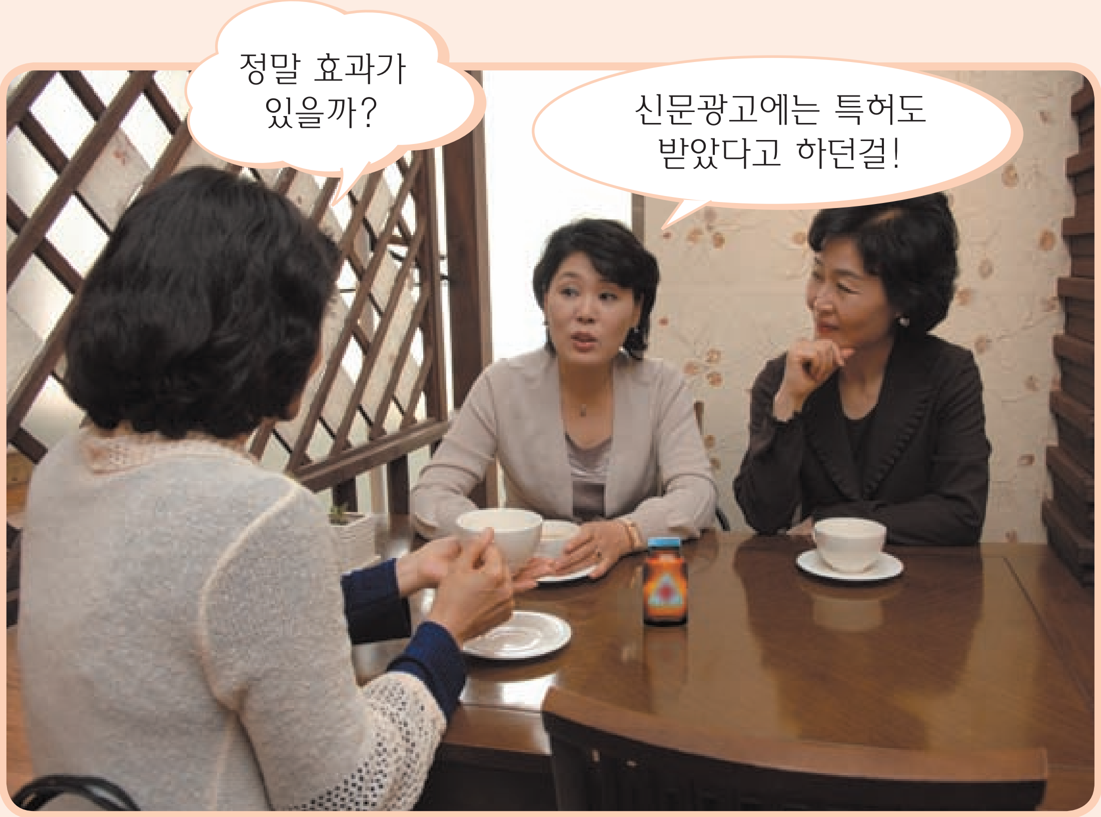
정말 효과가 있을까?
신문광고에는 특허도 받았다고 하던걸!

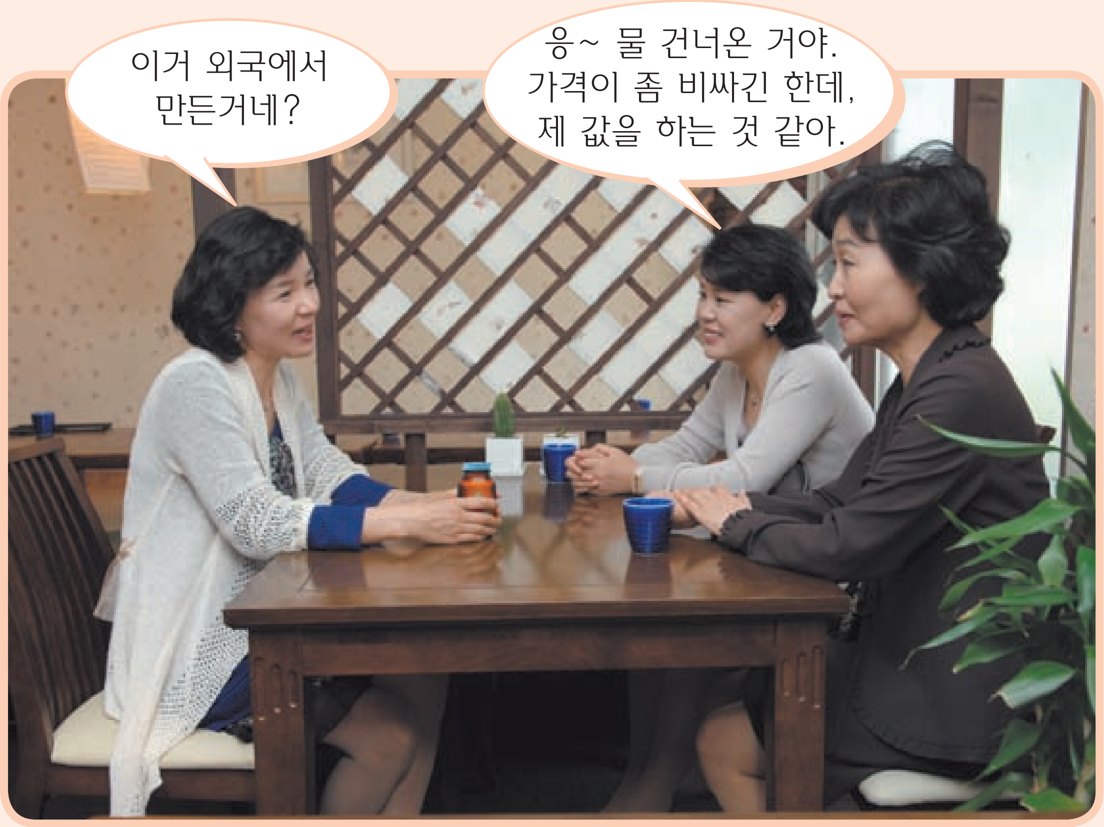
이거 외국에서 만든거네?
응~ 물 건너온 거야. 가격이 좀 비싸긴 한데, 제 값을 하는 것 같아.

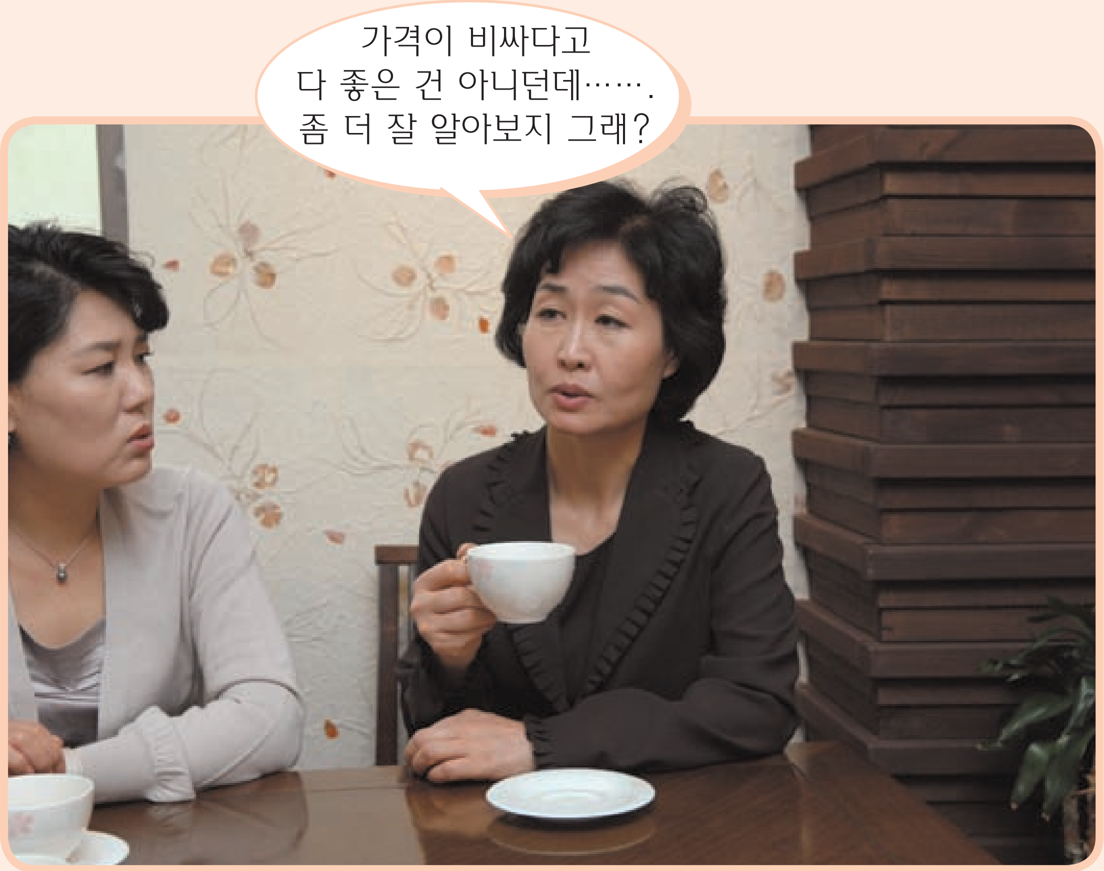
가격이 비싸다고 다 좋은 건 아니던데……. 좀 더 잘 알아보지 그래?

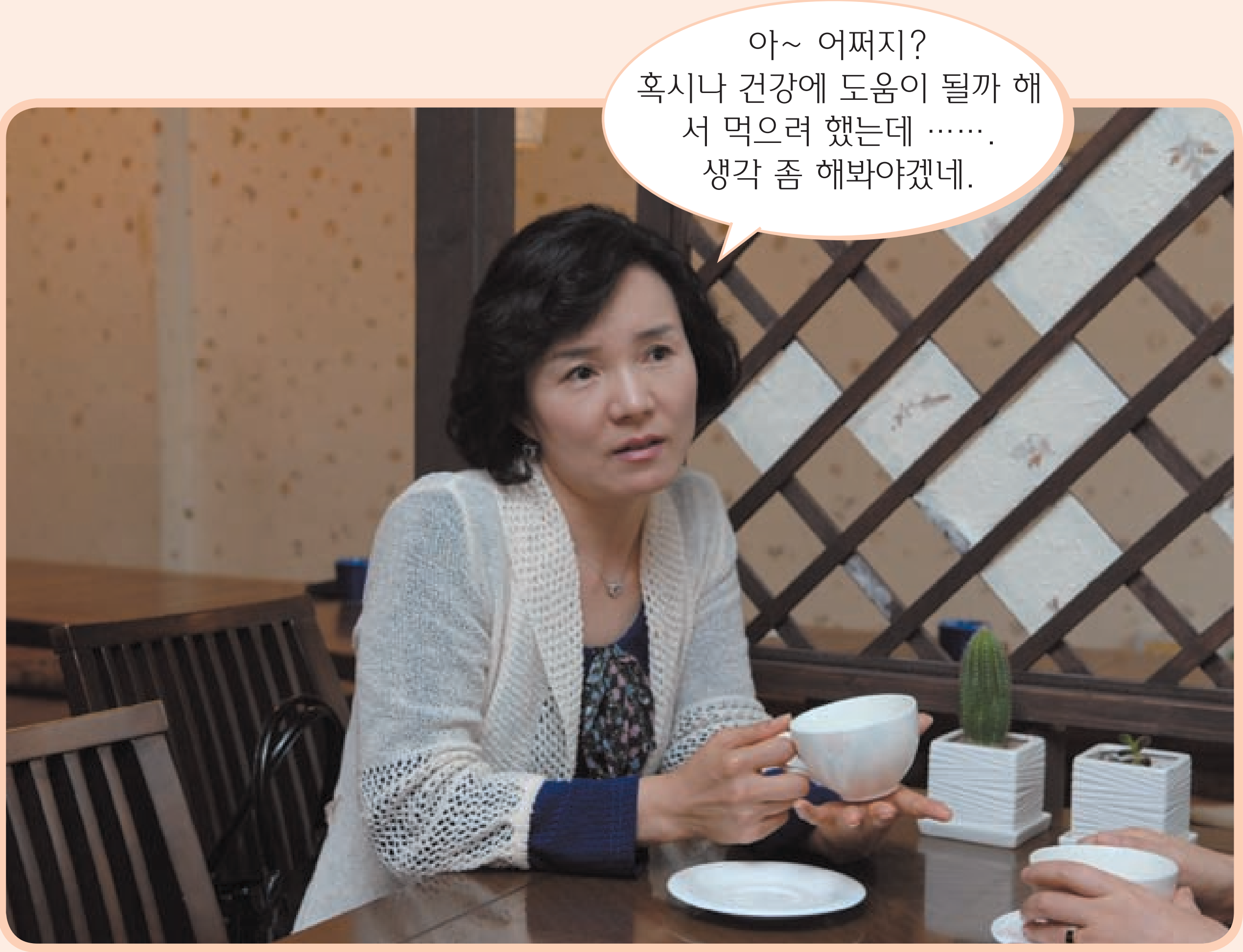

건강보조식품이란?

인체에 유용한 기능성을 가진 원료나 성분을 사용하여 정제 · 캅셀 · 분말 · 과립 · 액상 · 환 등의 형태로 제조 · 가공한 식품을 말합니다. 그 예로는 OO엑기스, OO버섯, O즙, OO화초 등이 있습니다.

집에 돌아온 민호엄마는 건강보조식품에 대해 고민을 한다.

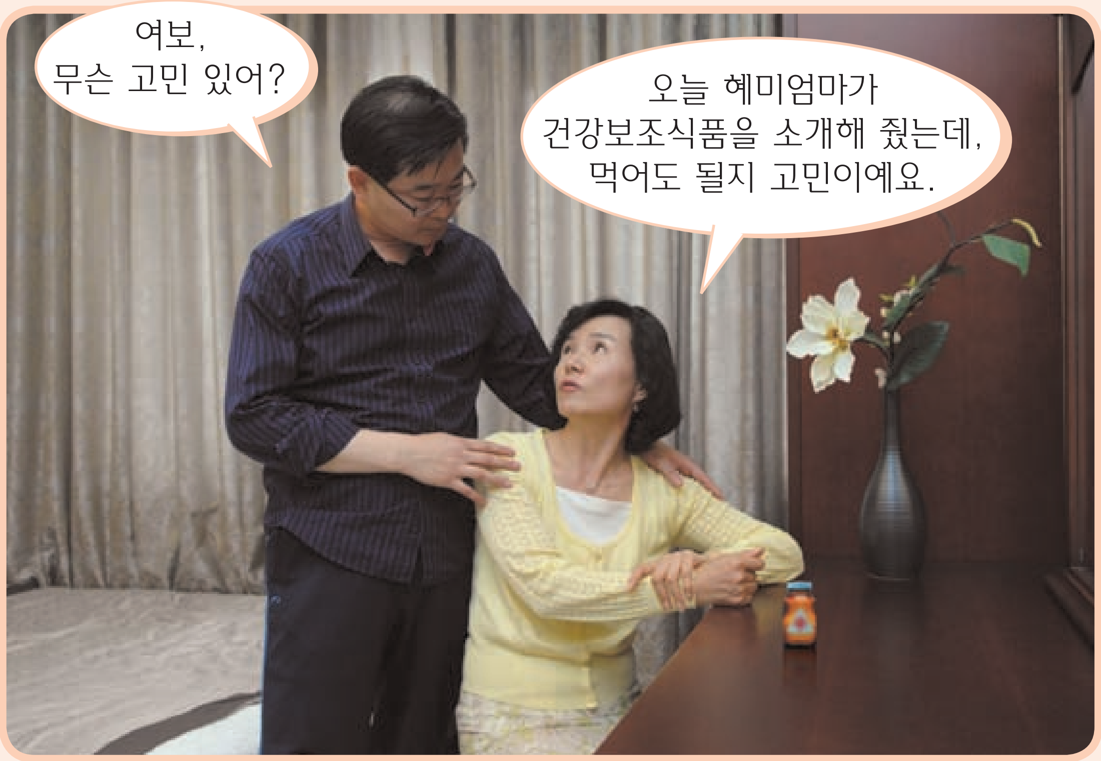

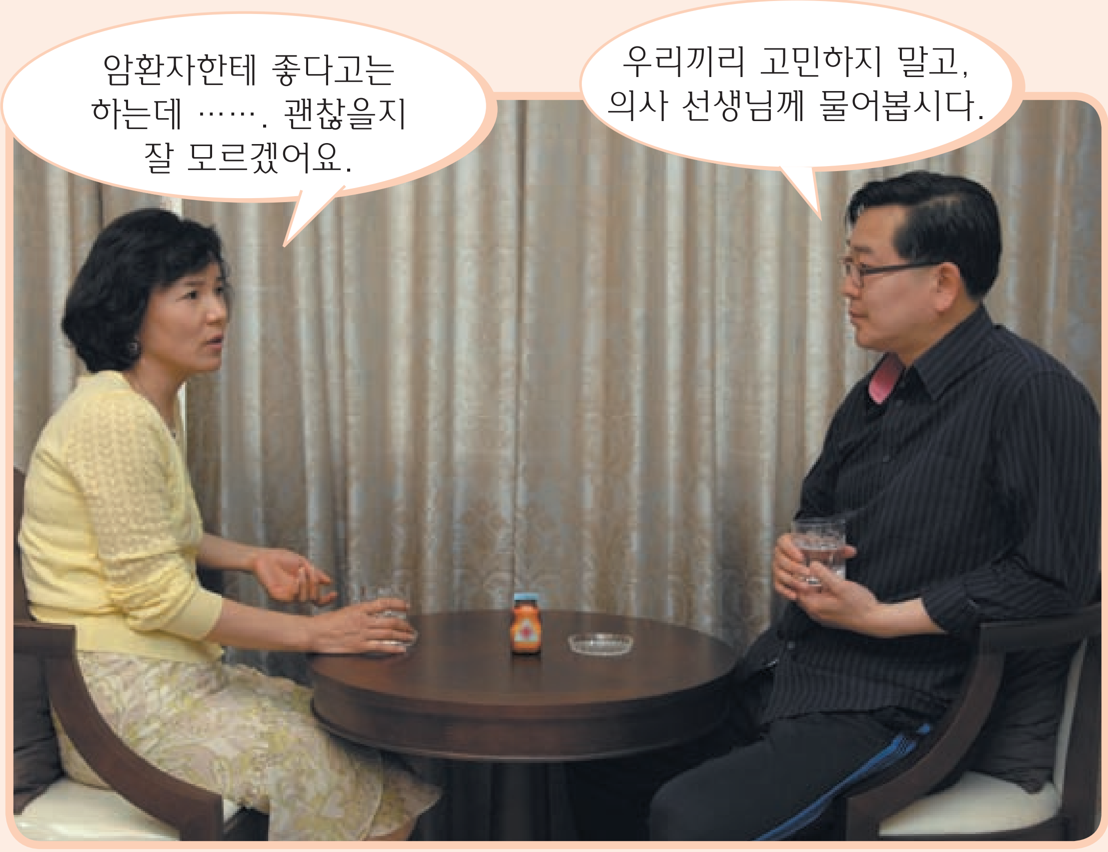

다음 날, 민호엄마는 병원에 정기진료를 받으러 가서
건강보조식품에 대해 의사 선생님께 물어본다.

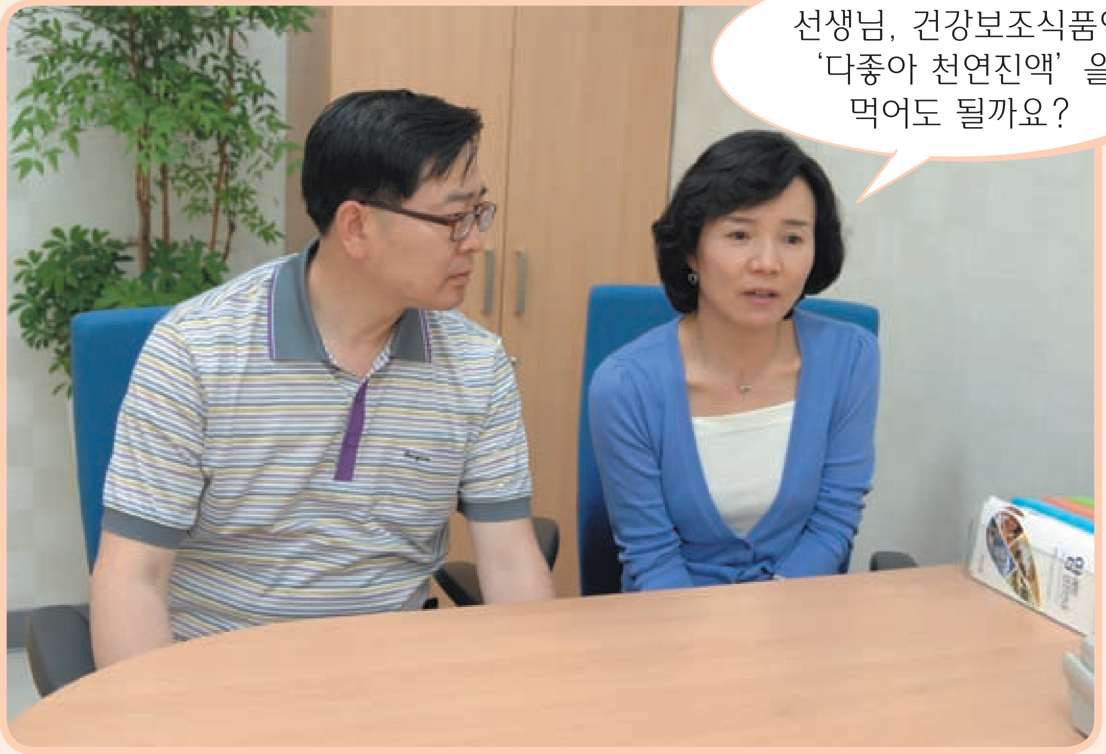

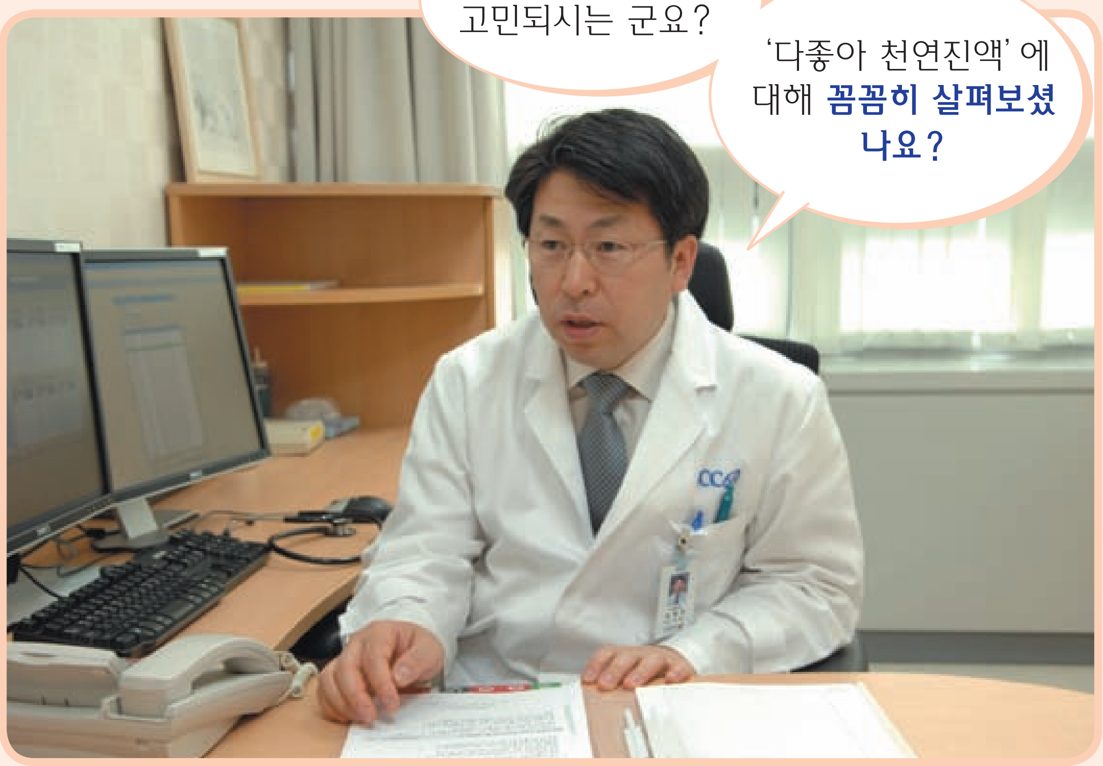

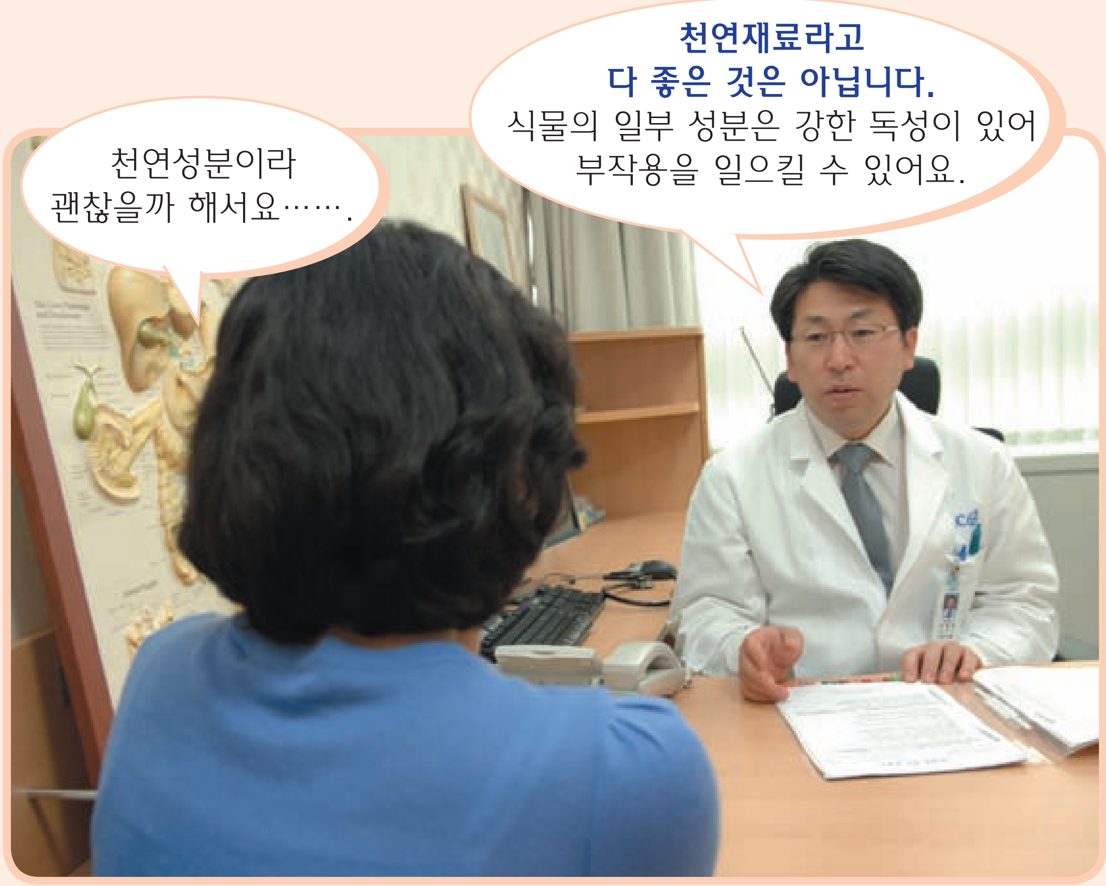
천연성분이라
괜찮을까 해서요…….
천연재료라고
다 좋은 것은 아닙니다.
식물의 일부 성분은 강한 독성이 있어
부작용을 일으킬 수 있어요.

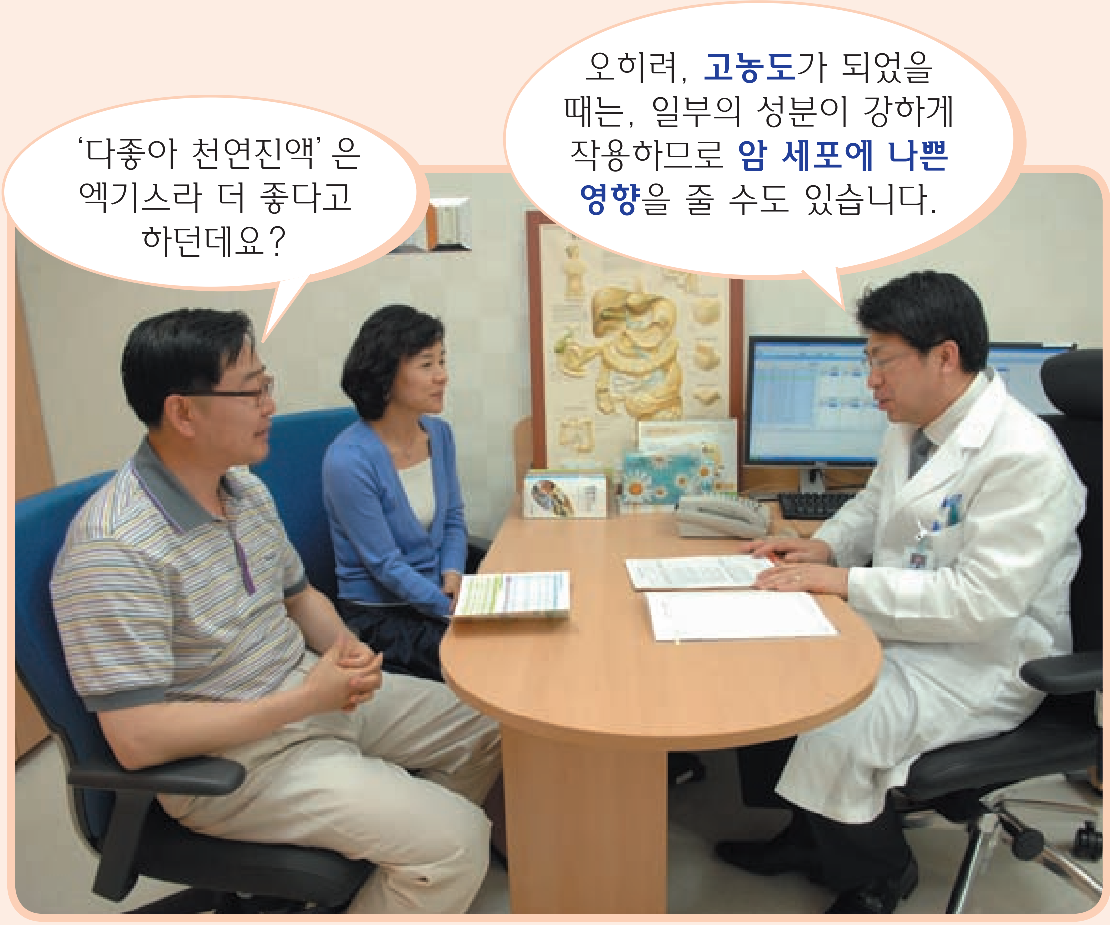
'다좋아 천연진액' 은
엑기스라 더 좋다고
하던데요?
오히려, 고농도가 되었을
때는, 일부의 성분이 강하게
작용하므로 암 세포에 나쁜
영향을 줄 수도 있습니다.

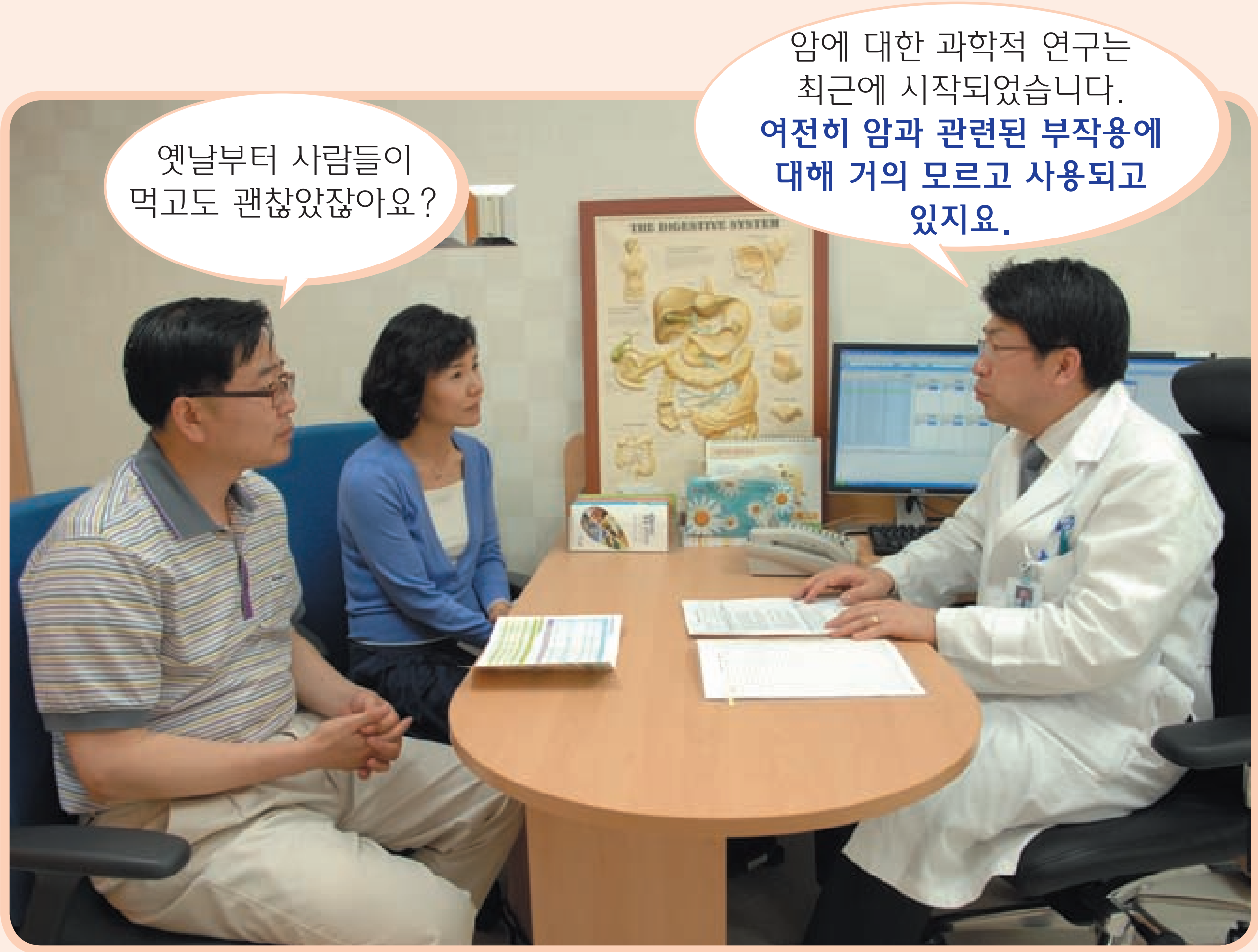
옛날부터 사람들이 먹고도 괜찮았잖아요?
암에 대한 과학적 연구는 최근에 시작되었습니다. **여전히 암과 관련된 부작용에 대해 거의 모르고 사용되고 있지요.**
THE DIGESTIVE SYSTEM

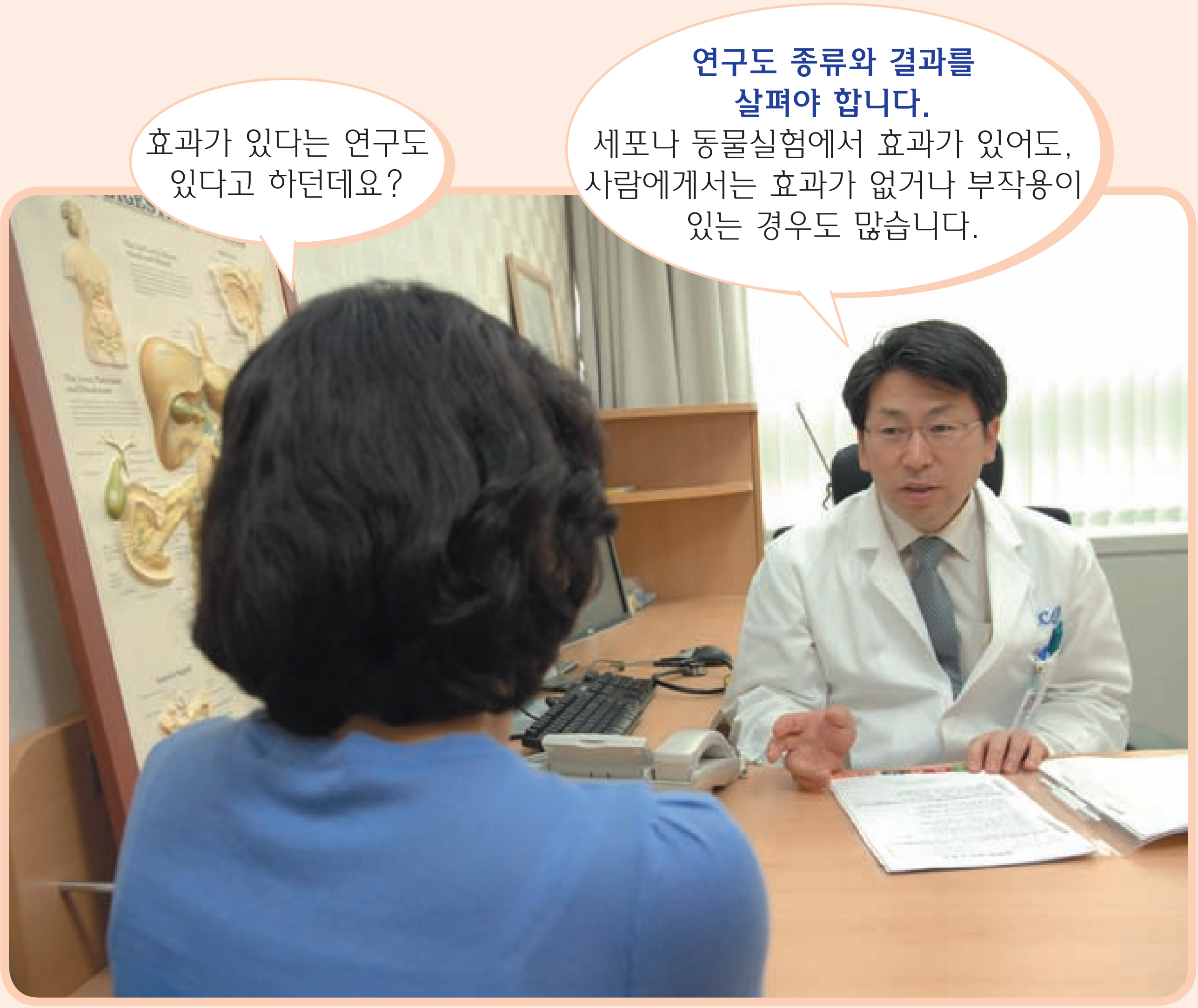
효과가 있다는 연구도 있다고 하던데요?
연구도 종류와 결과를 살펴야 합니다.
세포나 동물실험에서 효과가 있어도, 사람에게서는 효과가 없거나 부작용이 있는 경우도 많습니다.

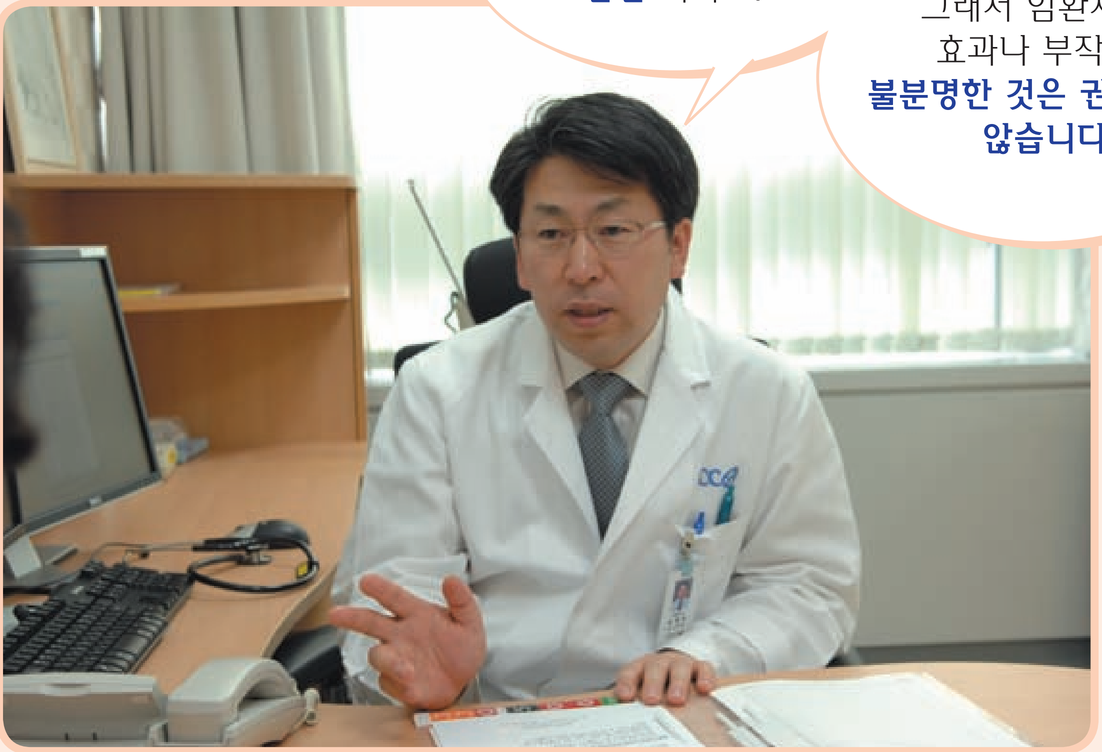
가장 중요한 것은 **혹시나 하는 효과보다 안전** 이지요.
그래서 암환자에게 효과나 부작용이 **불분명한 것은 권해드리지 않습니다.**

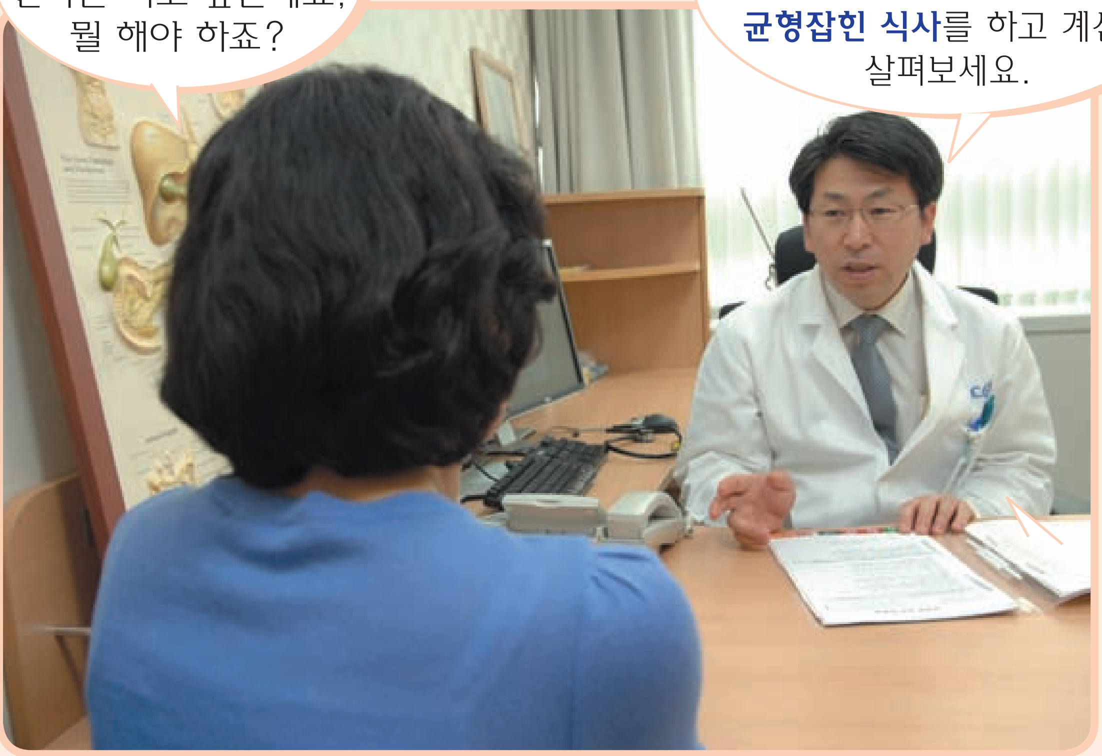
아, 그렇군요! 그래도 제 건강을 위해 뭔가는 하고 싶은데요, 뭘 해야 하죠?
우선 병원의 치료와 관리를 잘 받으세요. 그리고 영양과 관련해서는 **균형잡힌 식사**를 하고 계신지 살펴보세요.

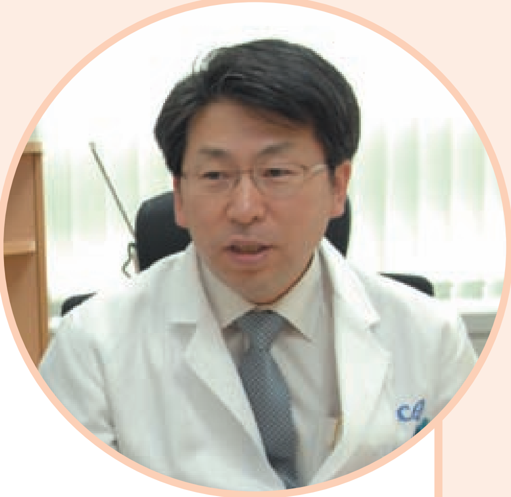

어떤 특정식품이나 영양소로 암을 치료할 수는 없습니다.

암 치료와 회복을 위해서는 좋은 영양상태를 유지해야 하며 이를 위해서는 **다양한 음식을 골고루 섭취**하십시오.

균형 잡힌 식사를 하지 않고 건강보조식품에 의존할 경우, 필수적인 영양이 부족해 회복이 느려지거나 과도한 성분이 암세포를 더 자극할 수 있습니다.

그래도, 건강보조식품을 고려한다면

- 이 제품이 **나에게 적합한지**
- **안전**하게 만들어졌는지
- 효과는 연구로 **입증** 되었는지
- **부작용**은 어떤 건지
- **장점과 단점**을 따져보는 것이 필요합니다.

실제로, 아무런 근거 없이 광고하는 것을 많이 봅니다.
(18쪽 참조)

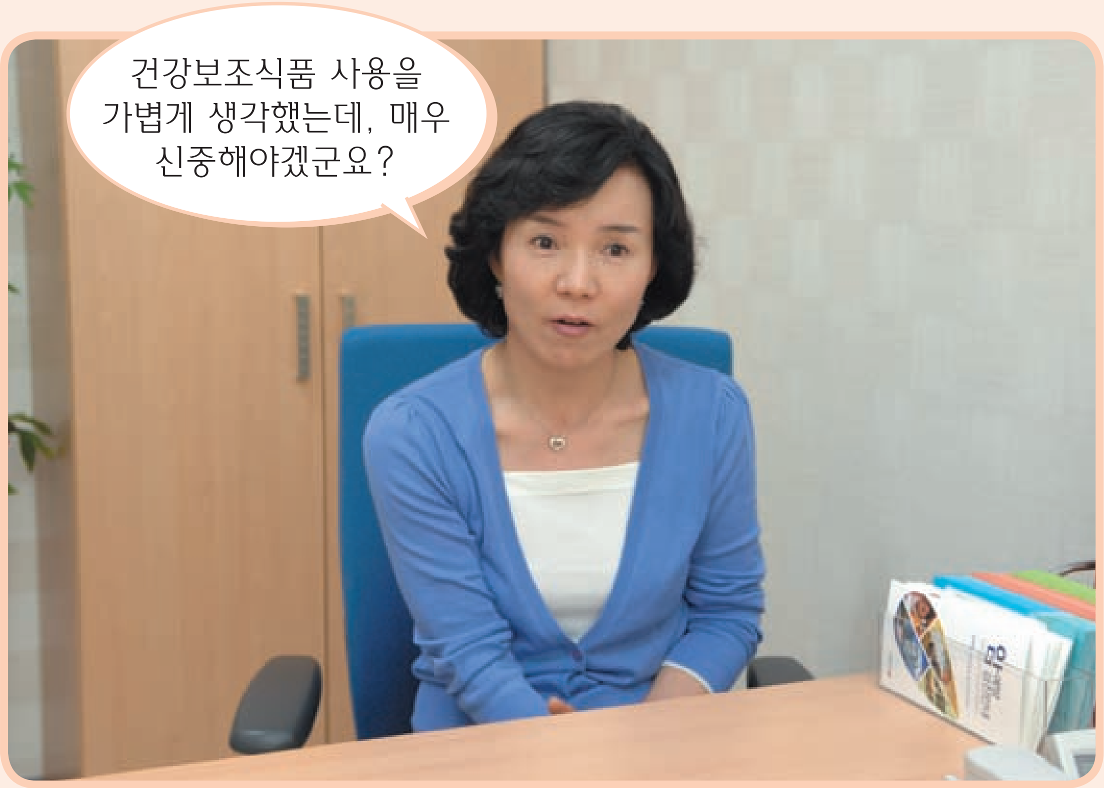
건강보조식품 사용을
가볍게 생각했는데, 매우
신중해야겠군요?

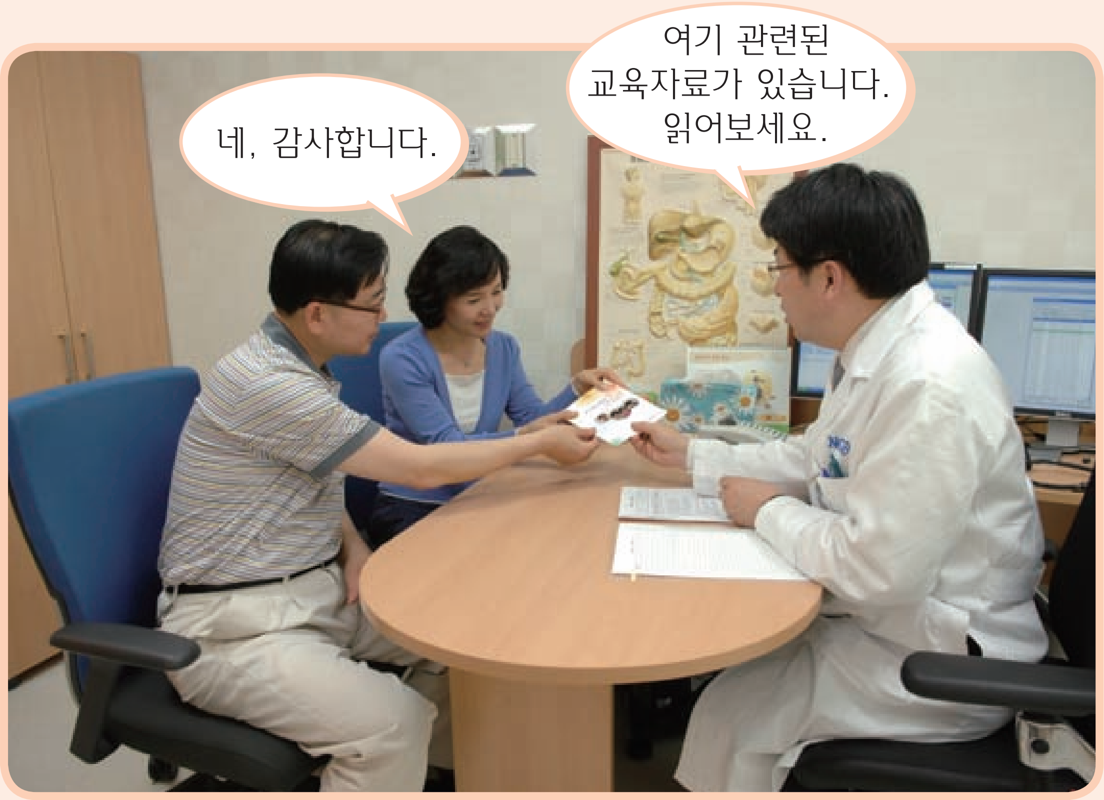
네, 감사합니다.
여기 관련된
교육자료가 있습니다.
읽어보세요.

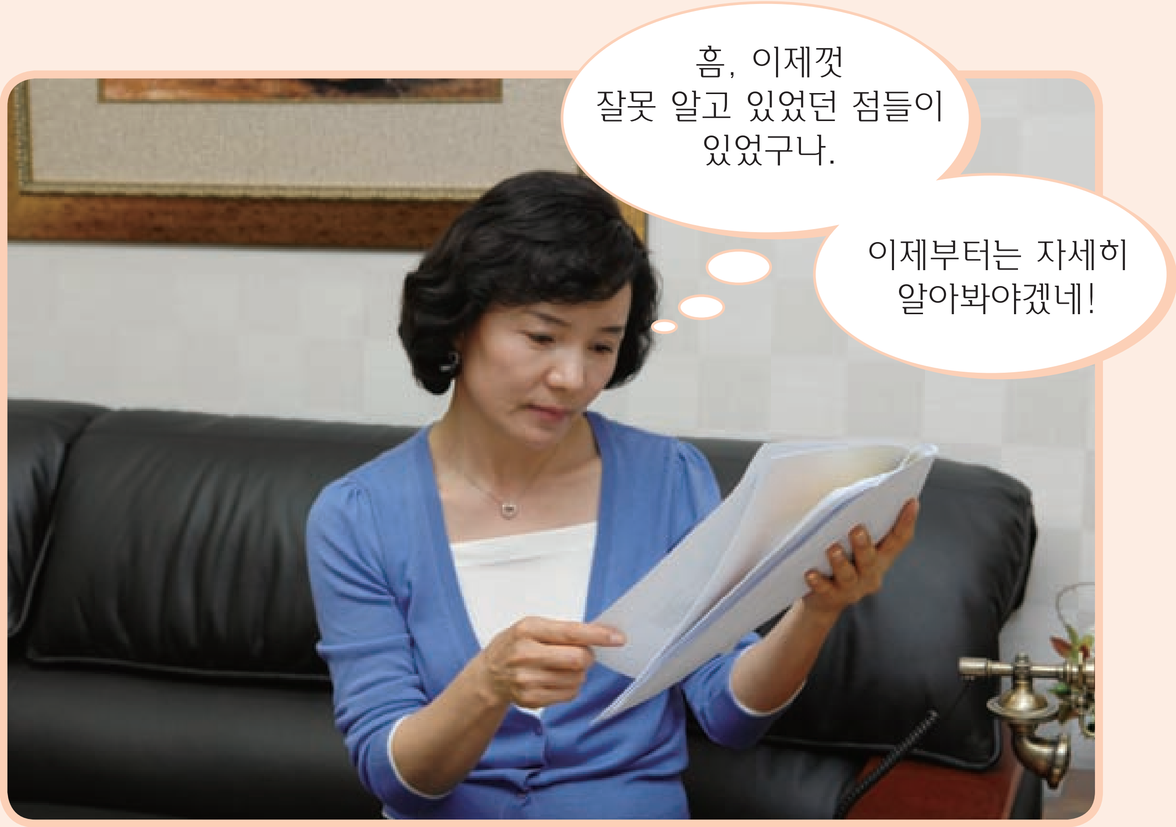

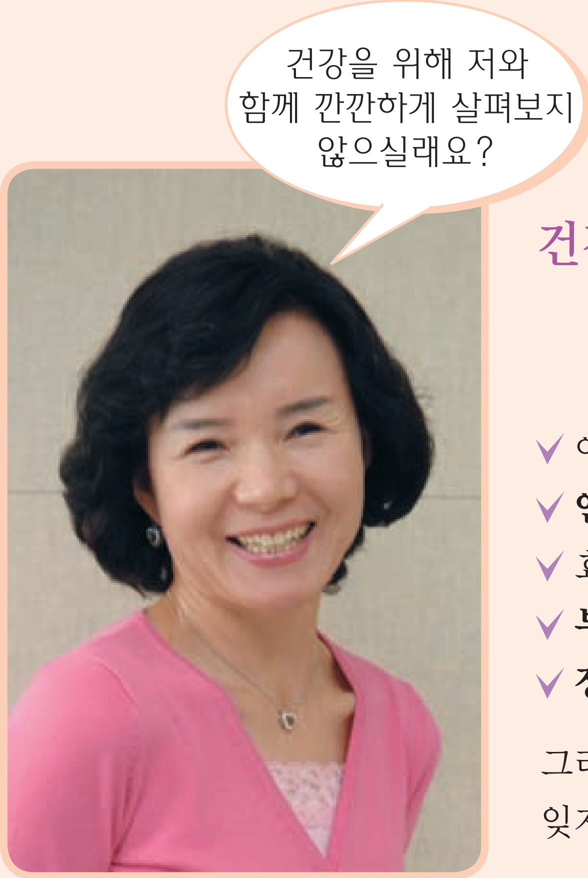

건강보조식품을 고려하실 때는,

- 이 제품이 **나에게 적합한지**
- **안전**하게 만들어졌는지
- 효과는 연구로 **입증** 되었는지
- **부작용**은 어떤 건지
- **장점과 단점**을 따져보는 것이 필요합니다.

그리고 의료진과 상의 하시는 것을 잊지 마세요.

건강보조식품에 대한 흔한 오해

건강보조식품에 대해 일반적으로 가지고 있는 오해에 대한 설명을 담았습니다.

천연의 자연 식물 원료는 안전하다?

천연의 자연 식물 원료라고 안전한 것은 아닙니다.

일부 식물원료라 하더라도 먹어도 되는 것과 안 되는 것이 있습니다. 또한 먹어도 되는 식물이라고 하더라도, 그 일부를 농축하거나 다려서 고농도로 먹게 되는 경우, 간기능이나 혈액수치, 면역상태에 영향을 줄 수 있습니다.

농축된 고농도 일수록 좋은 것이다?

고농도일수록 좋은 것이 아닙니다.

일반적으로 음식으로 영양을 섭취했을 때는 다양한 성분이 낮은 농도로 몸 속으로 들어와 균형되게 역할을 합니다. 그러나 고농도의 성분의 경우 치료중인 약물이나 암세포 등에 나쁜 영향을 줄 수도 있습니다.

옛날부터 전통적으로 사용되었던 것은 안전하다?

전통적인 보조식품이 암환자에게도 안전하다고 할 수는 없습니다.

암이라는 질환은 최근에 급속도로 증가된 병으로, 최근 30년 전부터 연구가 본격적으로 진행되었습니다. 따라서 예전부터 사용한 건강보조식품이라 하더라도 암환자에게 어떠한 영향을 줄지에 대해서는 구체적으로 연구가 안 된 것이 대부분입니다.

영양적으로 음식보다 건강보조식품을 통해 섭취하는 것이 더 효과적이다?

건강보조식품만으로는 완벽한 영양을 보충할 수 없습니다.

아무리 좋은 성분이더라도 집중적으로 섭취하면 전체적인 영양의 불균형이 오게 됩니다. 균형된 영양 섭취를 위해 가장 중요한 것은 여러 가지 음식을 골고루 잘 먹는 것입니다. (20쪽 참조)

건강보조식품 선택 시 고려해야 할 사항!

건강보조식품 선택 시 조심해야 할 광고 내용

– 모든 암을 완치한다고 확신하는 경우

– 모든 병에 다 좋다고 하는 경우

– 병원치료를 받지 못하게 하는 경우

– 부작용이 전혀 없고 효과만 있다고 하는 경우

– 단지 일부에서만 비밀리에 제공되는 경우

– 의료계나 과학계에서 논란이 있는 경우

옥석을 가리셔야 합니다. 꼼꼼히 따져보고 의료진과 논의하세요.

– 일부 건강보조식품의 경우는 암환자와 가족의 지푸라기라도 잡고 싶은 심정을 상업적으로 악용하여, 암환자들이 피해를 보는 사례가 종종 있습니다.

– 어떠한 요법이 암환자에게 도움이 된다고 말한다면, 나에게 도움이 될지 과학적 근거를 꼼꼼히 알아 보셔야 합니다. 건강보조식품에 대해 의료진과 이야기 해보세요.

건강보조식품 선택 시 꼼꼼히 따져보시고, 궁금한 사항은 주치의와 상담하세요.

식품의약품안전청 보도자료 (2009.6.16)

인터넷 등에서의 허위 · 과대광고 행위 적발

식품의약품안전청은 국내 · 외 인터넷 쇼핑몰과 일간지 등에서의 식품 허위, 과대광고 판매행위를 모니터링하여 304건의 **질병의 치료 등 의약품으로 오인 혼동할 우려**가 있는 내용으로 허위, 과대광고를 하거나 **미승인 원료 등을 사용한 제품**을 판매한 업체에 대해 행정처분을 요청하였다고 밝혔다. …… 식약청은 인터넷에서 소위 건강식품 등을 무분별하게 구입할 경우 **비위생적으로 제조되거나 인체에 위해한 성분이 포함**되어 있을 수 있으므로 인터넷 구매에 각별한 주의를 기울여 줄 것을 당부하였다. ……

※위반제품 예시(출처:http://www.kfda.go.kr)

제품명	주요 암 관련 위반 내용
AHCC	‘항암작용이 뛰어남 표고버섯균사체’라고 허위 과대 광고
Garlic factor (마늘성분)	‘…항암작용…해독작용 등이 있다’고 허위 과대 광고
로얄제리 캡슐	‘…항암성분 함유’라고 허위 과대 광고
코엔자임 Q10	‘…위암을 억제할 수 있으며 여러 질병과도 연관되어 있다’고 허위 과대 광고
활인차	‘항암효과, 당뇨,…등 효과가 있다’고 허위과대광고
영지버섯 과립차 외2건	‘항암작용, 혈압조절작용….등 효과가 있다’고 허위과대 광고
노루궁뎅이버섯 진액	‘항암효과, 위암, 간암에 탁월한 효과가 있다’고 허위 과대 광고
프로폴리스 캡슐형	‘항암, 살암, 조직재생, 면역부활,…’ 등 효과가 있다고 허위과대광고
고농축 프로폴리스	‘암-폐암, 직장암, 난소암, 대장암, 위암, 방광암, 자궁암, 유방암 …등의 질병 예방에 효과가 있다’고 허위과대광고

제품명	주요 암관련 위반 내용
아가리쿠스	‘위암, 자궁암, 당뇨병이 치료되었다’는 체험후기 등 허위과대광고
제피로스	‘독소와 숙변제거, 암,…등 갖가지 난치병에 탁월’ 등으로 허위과대광고
젓갈세트	‘항암 및 고혈압 치료에 탁월한 효과가 있다는 연구결과가 있다’는 허위 과대 광고
ACE5	‘암 치료 및 암 예방에 탁월한 세계 10대 슈퍼 푸드로 손꼽히는 천연 열대 과일들과 생로얄제리원액이 만난 천연 항암 건강식품’이라고 허위 과대 광고
상어연골	‘…암 예방, 면역조절효과’ 등의 내용으로 허위광고
블루베리	‘항암 작용…’등 효과가 있다고 허위과대광고
CLA	‘최근의 동물실험에서 유방암, 대장암, 전립선암, 피부암, 폐암세포가 억제되는 것으로 나타났으며…예방과 치료에 매우 좋다’고 허위 과대 광고
아마씨가루	‘항암 예방과 치료 후 회복증진…등에 효과가 있다’라고 허위 과대 광고
구아바 티백차	‘대장암,…등에 효과가 있다’고 허위광고
크랜베리 파우더	‘암…등에 효과가 있다’고 허위과대광고
게르마늄	‘임상적으로도 폐암, 방광암, 유방암,…백혈병,…등에 게르마늄이 놀라운 효과를 나타내고 있다’고 허위과대광고
울금	‘…암 치료의 보조제,…등에 효과가 있다’고 허위과대광고

※ 위와 관련된 자세한 사항은 식약청 홈페이지를 참조하세요(http://www.kfda.go.kr)
문의전화 식약청 식품관리과 T. 02-380-1633~4

– 이 내용은 단기간의 적발 사례로, 극히 일부에 지나지 않습니다. 이 외에도 다양한 제품들이 허위광고 되고 있습니다.

가장 중요한 식사 원칙!
좋은 영양상태를 유지하기 위해 “음식을 골고루 맛있게 먹는 것”입니다

● 건강하게 식사를 하는 요령은 다음과 같습니다.

– 아침, 점심, 저녁을 규칙적인 식사를 하며 반찬은 골고루 먹습니다.

– 매끼 단백질 반찬을 꼭, 충분히 섭취합니다.

  • 단백질이 풍부한 고기나 생선 또는 계란, 두부, 콩, 치즈 등을 드셔도 됩니다.

– 채소 반찬은 매끼 2가지 이상 충분히 먹습니다.

– 한 가지 이상의 과일을 하루 1~2번 정도 드시는 것이 좋습니다.

– 우유 및 유제품은 하루 한 컵(200ml) 이상 마십니다.

  • 우유가 잘 맞지 않는 경우에는 요구르트, 두유, 치즈 등을 대신 먹습니다.

– 양념과 조미료는 적당히 사용하여 너무 맵고 짜지 않게 해서 먹습니다.

– 씹기 힘든 경우나 삼키기 힘든 경우에는 다지거나 갈아서 먹습니다.

단, 면역력이 저하된 경우나 먹기 힘들 때에는 개별적으로 영양상담을 받으시기 바랍니다.

● 그래도 어떻게 무엇을 먹을지 고민이 되신다면 주저하지 말고 물어보세요.

– 암환자의 식사와 관련하여 고민이 되신다면, 의료진과 영양사 또는 국가암정보센터 (www.cancer.go.kr ☎1577–8899)로 문의하세요.

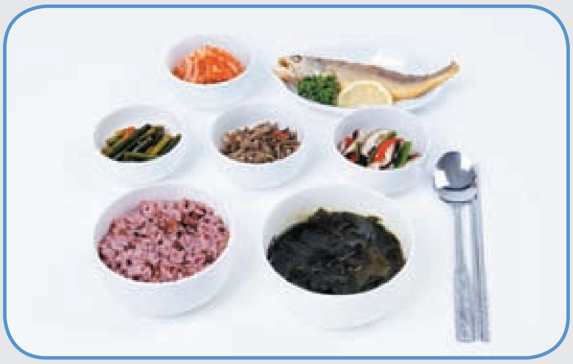

건강 밥상

영양상담은 암환자의 좋은 영양상태를 유지하는데 도움을 줄 것입니다.

자료 개발 자문 및 검토

조재일 (국립암센터 폐암센터)
김영우 (국립암센터 위암센터, 암예방검진센터)
오재환 (국립암센터 대장암센터)
정기욱 (국립암센터 유방암센터)
김석원 (국립암센터 유방암센터)
신동욱 (국립암센터 완화의료클리닉)
위경애 (국립암센터 임상영양실)
김정선 (국립암센터 암역학연구과)
심영목 (삼성서울병원 폐암센터)
김나연 (삼성서울병원 암교육센터)
이정언 (삼성서울병원 유방암센터)
김진용 (삼성서울병원 대장암센터)
김수연 (삼성서울병원 암교육센터)
김범석 (국립보건연구원 심혈관 · 희귀질환과)
서은영 (서울대학교 간호대학)

자료 개발에 도움을 주신 분들

등장인물	– 민호 엄마 : 임사라
	– 민호 아빠 : 서정민
	– 혜미 엄마 : 하수연
	– 영희 엄마 : 이재순
	– 의　사 : 김영우 (국립암센터 위암센터, 암예방검진센터)
촬영	김정민 (국립암센터 암정보교육과)
촬영 협조	국립암센터 암예방검진센터
	한식당 여자만

연구비지원	국립암센터 기관고유 연구사업
개발기관	국립암센터
개발자	장윤정 (국립암센터 암정보교육과, 암예방검진센터)
	조주희 (삼성서울병원 암교육센터)
	이수경 (국립암센터 암정보교육과)
개발연도	2009년 7월

이 교육자료는 국립암센터 기관고유연구사업 (Grant 0710190-3)연구 목적으로 개발되었습니다.

국민 암 예방 수칙

⊙ '국민 암 예방 수칙' 으로 암을 예방하세요.

- 담배를 피우지 말고, 남이 피우는 담배 연기도 피하기
- 채소와 과일을 충분하게 먹고, 다채로운 식단으로 균형 잡힌 식사하기
- 음식을 짜지 않게 먹고, 탄 음식을 먹지 않기
- 술은 하루 두 잔 이내로만 마시기
- 주 5회 이상, 하루 30분 이상, 땀이 날 정도로 걷거나 운동하기
- 자신의 체격에 맞는 건강 체중 유지하기
- 예방접종 지침에 따라 B형간염 예방접종 받기
- 성 매개 감염병에 걸리지 않도록 안전한 성생활 하기
- 발암성 물질에 노출되지 않도록 작업장에서 안전 보건 수칙 지키기
- 암조기검진 지침에 따라 검진을 빠짐없이 받기
